# A Comprehensive Review of Moroccan Medicinal Plants for Diabetes Management

**DOI:** 10.3390/diseases12100246

**Published:** 2024-10-09

**Authors:** Hanane Boutaj

**Affiliations:** 1 Laboratory of Life and Health Sciences, FMP, Abdelmalek Essaadi University, Tetouan 93000, Morocco; h.boutaj@uae.ac.ma; 2 Centre d’Agrobiotechnologie et de Bioingénierie, Unité de Recherche Labellisée CNRST (Centre AgroBiotech-URL-CNRST-05), Équipe “Physiologie des Stress Abiotiques”, Faculté de Sciences et Tecchniques, Université Cadi Ayyad, Marrakesh 40000, Morocco

**Keywords:** medicinal plants, ethnobotanical survey, *in vivo* and *in vitro* antidiabetic, Morocco

## Abstract

Moroccan flora, renowned for its diverse medicinal plant species, has long been used in traditional medicine to manage diabetes. This review synthesizes ethnobotanical surveys conducted during the last two decades. Among these plants, 10 prominent Moroccan medicinal plants are evaluated for their phytochemical composition and antidiabetic properties through both *in vitro* and *in vivo* studies. The review encompasses a comprehensive analysis of the bioactive compounds identified in these plants, including flavonoids, phenolic acids, terpenoids, and alkaloids. Phytochemical investigations revealed a broad spectrum of secondary metabolites contributing to their therapeutic efficacy. *In vitro* assays demonstrated the significant inhibition of key enzymes α-amylase and α-glucosidase, while *in vivo* studies highlighted their potential in reducing blood glucose levels and enhancing insulin secretion. Among the ten plants, notable examples include *Trigonella foenum-graecum*, *Nigella Sativa*, and *Artemisia herba-alba*, each showcasing distinct mechanisms of action, such as enzymatic inhibition and the modulation of glucose metabolism pathways. This review underscores the necessity for further chemical, pharmacological, and clinical research to validate the antidiabetic efficacy of these plants and their active compounds, with a view toward their potential integration into therapeutic practices.

## 1. Introduction

Diabetes is a chronic, multifactorial condition that is growing rapidly and affects millions of people worldwide [1]. In non-industrialized nations, 80% of cases are predicted to occur by 2025; this pandemic, which is mostly caused by type 2 diabetes (T2D), represents a disproportionately large social and economic cost [2]. It is estimated that the number of people with diabetes will reach 783 million by 2045 [3]. In Morocco, it represents a major public health problem, with an estimated prevalence of 6.6% and 10% of the population over 20 and 50 years, respectively [4]. The etiology of this metabolic disorder is either the insufficient pancreatic production of insulin or resistance to the effects of insulin [5]. According to the World Health Organization [6], common symptoms include excessive appetite (polyphagia), frequent urination (polyuria), thirst (polydipsia), weight loss, exhaustion, hazy eyesight, and sluggish wound healing.

Dietary and lifestyle factors, including obesity, physical inactivity and a diet high in glycemic index and low in fiber, are well-established contributors to the development of T2D [7]. Moreover, a number of problems, such as oxidative stress, activation of the polyol pathway, and an increased risk of cardiovascular disease, peripheral neuropathy, nephropathy, retinopathy, and other microvascular and macrovascular complications, can result from chronic hyperglycemia, a hallmark of uncontrolled diabetes [7,8].

Conventional treatment, such as dietary changes, insulin therapy, and oral hypoglycemic drugs, are commonly used in combination for diabetes management. However, alternative therapy options are being investigated, but oral hypoglycemic medications are expensive and can cause side effects such as skin rashes, nausea, liver issues, and heart failure [9]. Herbal medicine has emerged as a promising complementary or alternative therapy for diabetes management [10].

Phytotherapy is an integral part of Moroccan culture, where people have endogenous knowledge passed down from generation to generation. Traditional Moroccan medicine draws on its Islamic, Arab-Berber and European components, and is used to treat a wide range of illnesses. The use of medicinal plants to treat diabetes is common practice in different regions of Morocco, not least because of the prohibitive costs of modern treatments and the limited accessibility of modern medicines [11]. This review aims to identify and analyze the medicinal plants used in Morocco to treat diabetes, based on ethnopharmacological surveys carried out over the last twenty years, and to valorize this traditional knowledge for the potential production of improved medicines.

## 2. Methodology

Scientific databases of peer-reviewed academic literature, such as Scopus, Web of Science, Google Scholar, PubMed, Science-direct and Medline, were used to collect relevant research about Moroccan medicinal plants used in the treatment of diabetes published from January 2004 to July 2024. Different keywords were used such as “Ethnobotanical studies”, “Ethnobotanical survey”, “medicinal plants used in diabetes management”, “antidiabetic medicinal plants”, and “Moroccan medicinal plants and diabetes”. A literature search was conducted regarding the *in vitro* and *in vivo* assessment of the biological activity of Moroccan medicinal plants used in diabetes management. We reviewed collected data on the explored Moroccan regions (Fez, Meknes, Ksar Elkebir, Taza, Rabat-Salé-Kénitra, High Atlas Central, Tangier-Tetouan, Safi and Essaouira, Beni-Mellal-Khenifra, Casablanca-Settat, Errachidia, Al Haouz-Rhamn, Tan-Tan, Laayoune Boujdour Sakia El Hamra, Izarene, Middle atlas, Sidi Slimane, Chtouka Ait Baha and Tiznit, Moroccan Rif, Taroudant, Oriental Morocco (Oujda), Central Plateau, Guelmim, Agadir and Ouezzane). In this review, we screened a large volume of literature (824 articles) but focused on studies published between January 2004 and July 2024 that met the following inclusion criteria:

- Ethnobotanical surveys (38) of Moroccan medicinal plants used in diabetes management;
- Publications related specifically to *in vitro* (30) and *in vivo* (97), or both (8), studies of the 10 most widely used Moroccan antidiabetic medicinal plants;
- Studies published in peer-reviewed journals;
- Research works that included clear experimental methods and statistical analyses.

## 3. Results

### 3.1. Traditional Uses and Plant Sources

**Figure 1:**
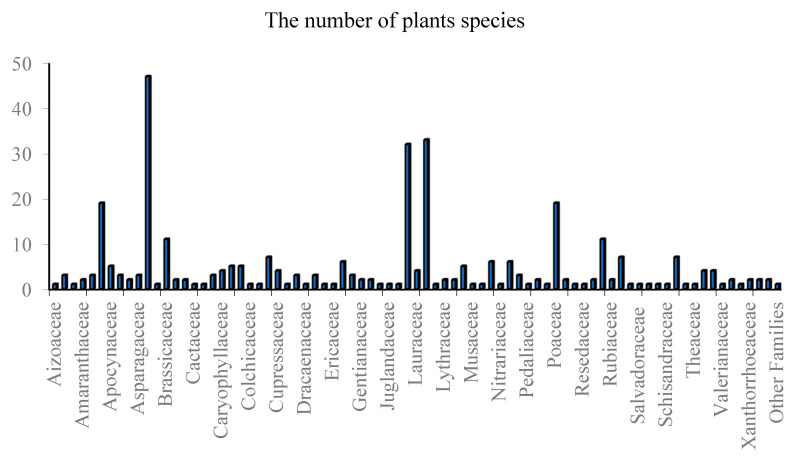
The botanical families used for diabetes management in Morocco.

Medicinal plants have traditionally been the main means of management of diabetes mellitus, which is the most common non-communicable disease. Moroccan local communities have developed a variety of herbal techniques used to manage diabetes. A total of 344 medicinal plants belonging to 79 families were highlighted in ethnobotanical surveys as traditional antidiabetic treatments in Morocco (Table 1, Figure 1). Among the families, Asteraceae, known as Compositae, showed the highest number of plants, followed by Leguminosae (Fabaceae), Lamiaceae, Poaceae (Graminaceae), Apiaceae, Brassicaceae, and Rosaceae (Figure 1). The Asteraceae family was the most frequently used in traditional Moroccan medicine, aligning with findings from other countries [12,13,14]. Asteraceae is recognized as the world’s largest flowering plant family, known for its medicinal properties [15]. Historical records document the traditional medicinal uses of various Asteraceae species, and several bioactive compounds within these plants have been studied for their potential health benefits [16].

**Figure 2:**
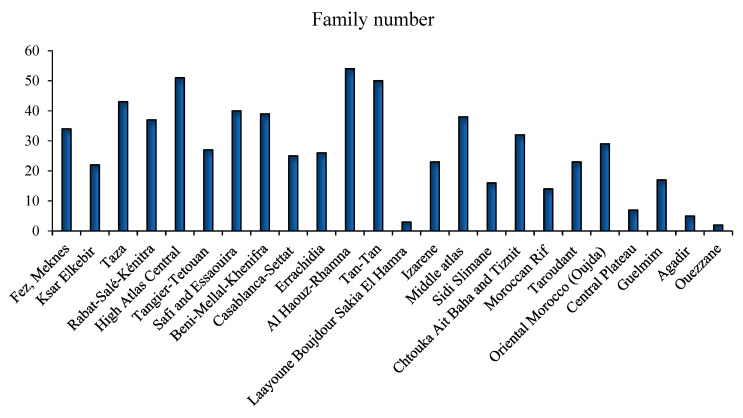
The distribution of plants species families per Moroccan regions.

**Figure 3:**
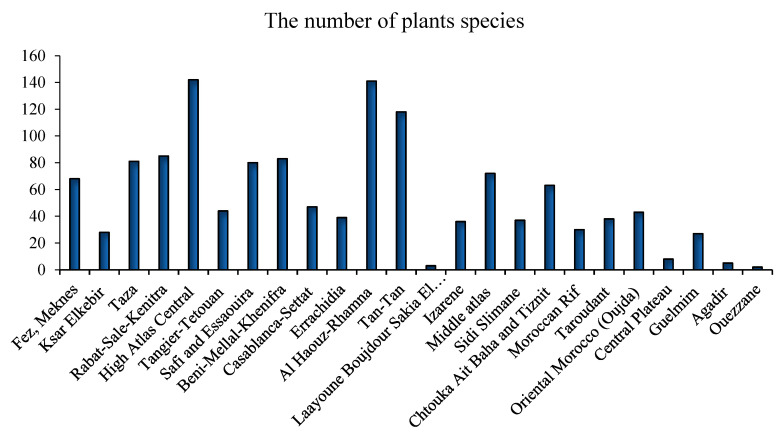
The distribution of plants species per Moroccan regions.

Some medicinal species have been reported for the first time as antidiabetic remedies in Morocco. The distribution of species used in diabetes management varies from one region to another (Figure 2) [17,18,19,20,21,22,23,24,25,26,27,28,29,30,31,32,33,34,35,36,37,38,39,40,41,42,43,44,45,46,47,48,49,50,51,52,53,54]. Al Haouz-Rhamna had the highest number of Moroccan medicinal plant species used in diabetes management, followed by the High Atlas Central region, Tan-Tan, Rabat-Sale-Kenitra, Beni Mellal-Khenifra, Taza, Safi and Essaouira, Fez-Meknes, Middle Atlas, and Chtouka ait Baha and Tiznit (Figure 3). Some plants species are concentrated only in the southern region, especially in Tan-Tan, such as *Opophytum theurkauffii*, *Searsia albida*, *Calotropis procera*, *Hyphaene thebaica*, *Artemisia reptans*, *Cichorium intybus*, *Saussurea costus*, *Nasturtium officinale*, *Capparis decidua*, *Maerua crassifolia*, *Silene vivianii*, *Atriplex halimus*, *Cynomorium coccinum*, *Cyperus rotundus*, *Ephedra alata*, *Ricinus communis*, *Acacia nilotica*, *Acacia Senegal*, *Arachis hypogaea*, *Ononis natrix*, *Ononis tournefortii*, *Vicia sativa*, *Vigna radiate*, *Musa paradisiaca*, *Eucalyptus camaldulensis*, *Limonium sinuatum*, *Cynodon dactylon*, *Panicum turgidum*, *Polypogon monspeliensis*, *Emex spinose*, *Chaenomeles sinensis*, *Rubia tinctorum*, *Datura stramonium*, and *Nardostachys jatamansi* [19,52].

**Table 1:** Moroccan medicinal plants used in the treatment of diabetes.

Family Name	Scientific Name	Local Name(s)	Region(s)	Used Part(s)	Mode(s) of Use	Citation Number	References
Aizoaceae	*Opophytum theurkauffii Maire* L.	âfzû	L	Leaves/Fruits	Dec/Pow	1	[19]
Alliaceae	*Allium cepa* L.	Bassla/Azalim	A-L, N-Q, T	Bulbs/Seeds/Roots	Pow/Raw	22	[17,18,19,20,21,22,23,24,25,26,27,28,29,30,31,32,33,34,35,36,37,51]
	*Allium sativum* L.	Touma/Tiskert	A-L, O-Q	Bulbs/Roots	Raw/Mac/Dec	19	[18,19,21,24,25,26,27,29,30,31,32,33,34,35,36,37,38,39,51]
	*Allium ampeloprasum var. porrum*	Borro/Leborrou	D	Bulbs/Stems	Raw/Ing with water	2	[28,32]
Aloeaceae	*Aloe vera* (L.) Burm. f.	Sebbar/Ssabra/Siber	D, F, H, K, L, T	Pulps/Leaves	Raw/Pow	7	[19,21,22,30,32,39,51]
Amaranthaceae	*Anabasis aretioides* Moq. & Coss. ex Bunge	Chajra ma yeharrekha rih/Salla	K, L	Aerial parts	Dec	2	[19,21]
	*Beta vulgaris* L.	Lbarba	R	Seeds	Inf	1	[45]
	*Spinacia oleracea* L.	Sabanikh	D	Leaves	Nd	1	[51]
Anacardiaceae	*Pistacia atlantica* Desf.	Btem/Igg/Drou	C, Q, H	Fruits	Inf/Dec	3	[25,41,44]
	*Pistacia lentiscus* L.	Trou/Tidekt/Drou	D, E, F, K, N, O	Leaves/Gums/Barks	Raw/Inf/Dec	6	[21,23,24,34,39,51]
	*Searsia albida* (*Schousb.*) Moffett	Zewaya/anaffis	L	Fruits	Pow	1	[19]
Apiaceae	*Ammodaucus leucotrichus* Coss.	Kamoun soufi	L, K, H, P	Seeds	Inf/Dec	4	[19,21,26,30]
	*Ammi majus* L.	Atrilal/Trilal/Rjel l’aghrabe	V	Whole plant	Inf	1	[49]
	*Ammi visnaga* (L.) Lam.	Bachnikha/Barghanisse	A, C-E, G, I-K, N-P, T	Inflorescences/Fruits/Seeds	Dec/Mac/Ing/Inf	15	[17,18,20,21,22,23,24,26,29,32,33,34,35,37,51]
	*Anethum foeniculum* L.	Shamrah/Fennel	C	Nd	Nd	1	[33]
	*Apium graveolens* L.	Krafess	A, C, D, H, P, W	Seeds/Aerial parts	Inf/Dec/Mac	6	[26,29,30,32,33,52]
	*Carum carvi* L.	Lkarwya	A, C-E, G-L, Q	Seeds	Dec/Pow/Inf	15	[17,18,19,21,25,27,29,30,32,33,34,35,37,41,51]
	*Coriandrum sativum* L.	Kosbor	A-E, G-K, O, P, T, W	Seeds/Leaves	Inf/Dec/Ing	16	[17,20,21,22,26,28,29,30,31,32,33,34,35,37,51,52]
	*Cuminum cyminum* L.	Kamoun	C, D, F, K, L	Seeds	Pow/Ing	6	[19,21,32,33,39,51]
	*Daucus carota* L.	Khizou	K, L, O	Roots	Jui/Puree	3	[19,21,24]
	*Eryngium ilicifolium Lam.*	Tasnant/Iglifin	Q	Stems and leaves	Dec/Pow	1	[25]
	*Ferula communis* L.	L-kelḫ/Uffāl/Taggwelt	G, R	Fruits/Roots/Flowers/Leaves	Dec/Pow/Inf	2	[35,45]
	*Foeniculum vulgare* Mill.	Nafaa/Hebet hlawa	A, C-E, G-L, P, Q, W, X	Seeds/Fruits	Dec/Inf	17	[17,18,19,21,25,26,27,29,30,32,34,35,37,41,51,52,53]
	*Pastinaca sativa* L.	Left lmahfour	H, I, Q	Roots	Raw	4	[25,27,30,37]
	*Petroselinum crispum* (Mill.) Fuss	Maadnouss	A-D, K, I, H, L, P, W	Seeds/Leaves	Inf/Dec/Raw	11	[19,21,26,27,29,30,31,32,33,37,52]
	*Petroselinum sativum* Hoffm	Mԑadnūs/Imẓi	G	Aerial parts/Whole plants	Jui/Dec	1	[35]
	*Pimpinella anisum* L.	Habbat hlawa	C-E, G-I, K, L, P, Q, T	Seeds	Dec/Inf/Pow/Ing	13	[19,21,22,25,26,27,28,30,32,33,34,35,37]
	*Ptychotis verticillata* Duby	Nounkha	O	Aerial parts	Inf	1	[24]
	*Ridolfia segetum* (L.) Moris	Tebch	E, K, R	Seeds	Pow	3	[21,34,45]
Apocynaceae	*Apteranthes europaea* (Guss.) Murb.	Oukan iddan	Q	Stems	Dec/Inf/Raw	1	[25]
	*Calotropis procera* (Aiton) Dryand.	Turja	L	Leaves	Pow	1	[19]
	*Caralluma europaea* (Guss.) N.E.Br.	Daghmous	A, B, D, E, K, H, P, S, V	Aerial parts/Leaves/Rackets/Roots	Mac/Jui/Pow/Dec/Inf/Per	10	[21,26,29,30,31,32,34,44,46,49]
	*Nerium oleander* L.	Defla/Alili	A, C-L, N, P, Q, S, T, W, Y	Leaves	Dec/Inf/Mac/Fum	23	[17,18,19,21,22,23,25,26,27,32,33,34,35,36,37,39,41,44,46,48,50,51,52]
	*Periploca laevigata* subsp. *Angustifolia* (Labill.) Markgr.	Asllif	Q, S	Fruits/Leaves	Dec	2	[25,46]
Arecaceae	*Chamaerops humilis* L.	Dum/Tiguezden	C-E, H, K, O, Y	Leaves/Fruits/Roots	Raw/Dec/Inf/Pow	7	[21,24,30,32,33,34,50]
	*Hyphaene thebaica* (L.) Mart.	Dum/karur	L	Fruits	Pow	1	[19]
	*Phoenix dactylifera* L.	Tmar/Nkhil	E-H, K, L, P, J	Fruits/Seeds/Leaves/Pulps/Roots	Raw/Dec/Pow/Inf/Vin	8	[17,19,21,26,30,34,35,39]
Aristolochiaceae	*Aristolochia baetica* L.	Tiswik nigrane/Berztem	S	Roots/Resins	Pow	1	[46]
	*Aristolochia longa* subsp. *Fontanesii* Boiss. & Reut.	Berztem	A, G, H, K, L, T	Seeds	Pow/Dec	6	[18,19,21,22,30,35]
Asparagaceae	*Agave americana* L.	Ssabra/Sayber	K	Leaves	Dec	1	[21]
	*Asparagus albus* L.	Sekkum/Azzu	E, O	Young sprouts/Roots	Raw/Dec	2	[24,34]
	*Asparagus officinalis* L.	Saklaim	V	Stems	Coo in steamer, or water	1	[49]
Asteraceae	*Achillea odorata* L.	Elqorte	E, K	Leaves and flowers	Inf	2	[21,34]
	*Achillea santolinoides Lag.*	Chouihiya, El-qorte	E	Capitulum	Inf	1	[34]
	*Anacyclus pyrethrum* (L.) Lag.	Iguntas/Tagundecht/Takntist	O	Roots/Leaves	Inf/Pow	1	[24]
	*Antennaria dioica* (L.) Gaertn	Ouden elfar	K	Leaves	Dec	1	[21]
	*Anvillea garcinii* subsp. *Radiata* (Coss. & Durieu)	Negd	L, T	Leaves/Roots	Pow/Dec	2	[19,47]
	*Artemisia abrotanum* L.	Chih	K	Aerial parts	Dec	1	[21]
	*Artemisia absinthium* L.	Chiba	A-F, H, I, K, J, O, N, P, V	Aerial parts/Stems/Leaves	Inf	17	[17,18,20,21,23,26,27,29,30,31,32,33,34,37,39,49,51]
	*Artemisia arborescens* (*Vaill.*) L.	Šība/Šība šmaymiya	F	Aerial parts/Leaves	Inf	1	[35]
	*Artemisia atlantica* Coss. & Durieu	Chih ourika	K	Aerial parts	Inf	1	[21]
	*Artemisia campestris* L.	Chihi khorayss	E	Whole plant	Inf	1	[34]
	*Artemisia herba-alba* Asso	Izri/Chih dwidi	A, C-E, G-L, N-Q, S, T, W	Stems/leaves/Roots	Dec/Inf/Pow	23	[17,18,19,20,21,22,23,25,26,27,28,29,30,32,33,34,35,37,41,46,48,51,52]
	*Artemisia herba alba Assac.*,	Chih	N	Aerial parts/Leaves	Dec/Inf/Pow	1	[23]
	*Artemisia mesatlantica Maire*	Chih elkhryassi	E, K	Whole plant/Aerial parts	Dec	2	[21,34,48]
	*Artemisia reptans* C. Sm. ex Link	Chihiya	L	Leaves	Dec	1	[19]
	*Atractylis gummifera Salzm. ex* L.	Addād/Ddād,	G	Roots	Inf	1	[35]
	*Calendula arvensis* Bieb.,	Jemra Azwiwel	C, R	Flowers/Stems	Inf/Dec	2	[41,45]
	*Centaurea maroccana* Bal	Bejjaae nhal/Nogguir	D, K	Flowers	Inf	2	[21,51]
	*Chamaemelum mixtum* (L.) Alloni	Hellala	D	Flowers	Inf	1	[32]
	*Chamaemelum nobile* (L.) All.	Babounj	A, D, E, H, K, T	Leaves/Flowering tops	Dec/Inf	6	[21,22,29,30,32,34]
	*Chrysanthemum coronarium* L.	Hmessou	E	Flowers	Inf	1	[34]
	*Cichorium intybus* L.	Buaggad	L	Roots	Inf	1	[19]
	*Cladanthus arabicus* (L.) Cass.	Taafs	E, K	Flowers	Inf	2	[21,34]
	*Cladanthus scariosus* (Ball) Oberpr. & Vogt	Arzgi/irzgi	S	Flowers	Dec	1	[46]
	*Cynara cardunculus* L.	Kharchouf/Taggua	A, D, E, K, H, J, P, T, L	Aerial parts/Stems	Pow/Dec/Inf	10	[17,18,19,21,22,26,30,32,34,47]
	*Cynara cardunculus* subsp. *scolymus* (L.)	Lqoq	D, E, Q, T	Roots/Inflorescences	Dec/Inf	4	[25,32,34,47]
	*Cynara humilis* L.	Ṭimṭa/Ḥekk/Ḫeršūf	G	Roots	Dec/Pou	1	[35]
	*Dittrichia viscosa* (L.) Greuter	Terehla/Bagraman	B-D, E, K, O, S	Leaves/Stems/Fruits	Dec/Inf	8	[21,24,31,33,34,41,46,51]
	*Echinops spinosissimus Turra*	Taskra	Q, S, T	Flowers	Dec	3	[22,25,46]
	*Helianthus annuus* L.	Nouaratchamess	R, H	Roots/Seeds	Pow/Inf	2	[44,45]
	*Inula conyza* (*Griess.*) *DC.*	Terrehla	K	Roots	Dec	1	[21]
	*Inula helenium* L.	Terrehla damnatiya	K	Leaves/Flower	Dec	1	[21]
	*Lactuca sativa* L.	Khes/Lkhoss	E, K, H, P, R	Leaves	Raw/Inf	5	[21,26,30,34,45]
	*Launaea arborescens* (Batt.) Murb.	Iferskel/Moulbna	K, Q, L	Stems/Leaves/Roots Flowers	Pow/Dec/Inf	3	[19,21,25]
	*Matricaria chamomilla* L.	Mansania/Lbabounj	C, E, K, H, I, N	Leaves/Flowers	Dec/Inf	7	[21,23,27,33,34,37,41]
	*Pallenis spinosa* (L.) Cass.	Nugd/Nouged	E, K	Aerial parts/Whole plant	Dec/Inf	2	[21,34]
	*Saussurea costus* (*Falc.*) *Lipschitz*	Qist Hindi	W	Stems	Pow	1	[52]
	*Scolymus hispanicus* L.	Gurnina/Taghdiut	D, E, K, O, S	Stems/Leaves/Roots	Raw/Dec/Inf	5	[21,24,34,46,51]
	*Scorzonera undulata* Vahl	Tamtla	Q	Flowers	Raw	1	[25]
	*Seriphidium herba-alba*	Chih	X	Nd	Nd	1	[53]
	*Sonchus arvensis* L.	Kettan elhench/Tifaf	E, H, T	Leaves	Inf/Dec	3	[22,30,34]
	*Sonchus asper* (L.) *Hill*	Tifaf	R	Whole plants	Dec	1	[45]
	*Sonchus tenerrimus* L.	Tifaf	L, R	Leaves	Dec	2	[19,45]
	*Stevia rebaudiana Willd.*	Stevia	D, F	Leaves	Inf/Pow	2	[39,51]
	*Silybum marianum* L.	Chouka	D	Leaves/Fruits	Nd	1	[51]
	*Tanacetum vulgare* L.	Lbalssam	E, K, R	Stems/Leaves	Inf	3	[21,34,45]
	*Taraxacum campylodes* G.E. Haglund	Lhandba/Chlada	C, K	Flowers/Roots/Leaves	Dec/Pow	2	[21,41]
	*Warionia saharae Benthem* ex Benth. & Coss.	Afssas	Q, L, J	Leaves	Inf/Pow	3	[19,25,38]
Berberidaceae	*Berberis vulgaris* subsp. *Australis* (Boiss.) Heywood	Arghis/Atizar	D, E, G, C, K	Leafy stem/Barks/Fruits	Dec	5	[21,33,34,35,51]
Brassicaceae	*Anastatica hierochuntica* L.	Chajarat Maryem/lkemcha	E, L, O, R, W	Stems/Leaves	Pow/Inf	5	[19,24,34,45,52]
	*Brassica napus* L.	Left	L, H	Rhizomes	Jui	2	[19,30]
	*Brassica nigra* (L.) K. Koch	Elkhardel	K	Flowers	Pow/Inf	1	[21]
	*Brassica oleracea* L.	Krunb mkawar/Melfuf	C-E, H, K, L, O, P, R	Aerial parts/Fruits	Raw/Mac/Pou	9	[19,21,24,26,30,32,33,34,45]
	*Brassica rapa* L.	Left beldi	D, E, K, O	Roots/Leaves	Dec/Inf	5	[21,24,34,48,51]
	*Diplotaxis pitardiana* Maire	Kerkaz/Elharra	K, L	Flowers	Pow	2	[19,21]
	*Eruca vesicaria* (L.) Cav.	Ljerjir/Al girjir	D, E, H, L	Aerial parts	Jui/Pow	3	[19,30,34,51]
	*Lepidium sativum* L.	Hab errechad	A-L, P, W	Seeds	Mac/Pow/Dec/Inf	18	[17,18,19,21,26,27,28,29,30,31,32,33,34,35,37,39,41,51,52]
	*Nasturtium officinale* R.Br.	Gernunes	L	Leaves/stems	Mac	1	[19]
	*Ptilotrichum spinosum* (L.) Boiss.	Aguerbaz	O	Leaves/stems	Dec	1	[24]
	*Raphanus raphanistrum* subsp. *sativus* (L.)	Lfjel	A, D, E, K, H, I, Q, L, P	Roots/Bulbs	Raw/Inf/Mac	10	[19,21,25,26,27,29,32,34,37,51]
Burseraceae	*Boswellia sacra* Flueck.	Louban Dakar/Salabane	D, E	Resins/Fruits	Inf/Ing/Dec	2	[32,34]
	*Commiphora myrrha* (*Nees*) *Engl.*	Lmorra	A	Resins	Dec	1	[29]
Buxaceae	*Buxus balearica Lam.*	Azazer/lbakous	K, O	Leaves	Dec	2	[21,24]
	*Buxus sempervirens* L.	Lbeks	A	Leaves	Dec	1	[18]
Cactaceae	*Opuntia ficus indica* (L.) *Mill.*	Lhndia/Aknari	A-D, F-H, J, K, L, O-Q, T	Stems/Roots/Flowers/Seeds/Fruits	Dec/Jui/Pow/Inf/Raw/Oil	18	[17,19,20,21,22,24,25,26,27,29,30,31,32,33,35,39,41,51]
Capparaceae	*Capparis decidua* (*Forssk.*) *Edgew.*	Ignin	L	Fruits	Pow	1	[19]
	*Capparis spinosa* L.	Kabar/Taylulut	A, C-E, G, K, J, L, N, O, S, W	Aerial parts/Fruits/Roots	Pow/Dec/Inf	12	[17,18,19,21,23,24,34,35,41,46,51,52]
	*Maerua crassifolia Forssk.*	Atil/Sedra lkhadra	L	Leaves	Pow/Dec	1	[19]
Caryophyllaceae	*Herniaria glabra var. hirsuta* (L.) *Kuntze*	Hrasset lehjer	G	Aerial parts	Dec/Pow	1	[35]
	*Paronychia argentea* Lam.	Tahidourt n’imksaoum	S	Leafy stems	Inf	1	[46]
	*Silene vivianii* Steud.	Gern lebzal	L	Stems	Raw	1	[19]
	*Corrigiola telephiifolia Pourr.*	Sergina/Tasergint/Bakur al barbar	C, K, H, O, V	Roots	Pow	5	[21,24,30,33,49]
Cannabaceae	*Cannabis sativa* L.	Al lkif	F	Seeds/Leaves/Flowers	Pow	1	[39]
Cistaceae	*Cistus albidus* L.	Boutour	O	Leaves	Dec	1	[24]
	*Cistus creticus* L.	Irgel	K, Q, S	Leaves	Dec/Pow	3	[21,25,46]
	*Cistus laurifolius* L.	Agullid	E, K, S	Seeds/Flowers	Pow	3	[21,34,46]
	*Cistus salviifolius* L.	Irgel/Tirgelt	D, K, Q	Leaves/Seeds	Dec/Pow	3	[21,25,51]
	*Cistus ladanifer* L.	Touzalt	E	Leaves	Dec	1	[34]
Chenopodiaceae	*Atriplex halimus* L.	Legtef	L	Leaves	Pow/Dec/Mac	1	[19]
	*Chenopodium ambrosioides* L.	Mkhinza	A-C, E, G-J, W	Leaves/Aerial parts	Inf/Mac	10	[27,29,30,31,35,37,38,41,42,52]
	*Hammada scoparia* (Pomel) Iljin	Assay/Rremt	Q, M	Seeds/Leaves	Dec	2	[25,54]
	*Salsola tetragona* Delile	Laarad	L, J	Leaves and fruits	Pow	2	[19,43]
	*Suaeda mollis* Dest.,	Adeghmous	J	Aerial parts	In meals	1	[43]
Colchicaceae	*Androcymbium gramineum* (Cav.) J.F. Macbr.	Temrate leghrab	K	Bulbs	Inf	1	[21]
Convolvulaceae	*Ipomoea batatas* (L.)	Batata hlouwa	A	Roots	Raw	1	[29]
Cucurbitaceae	*Bryonia dioica Jacq.*	Terbouna	E	Stems/Fruits	Dec	1	[34]
	*Citrullus colocynthis* (L.) Schrad.	Aferziz/lhdej	A, C-E, G, H, K, L, M, O-S	Seeds/Fruits	Dec/Cat/Pow/Ing	15	[18,19,21,24,25,26,28,30,32,33,34,35,45,46,54]
	*Citrullus vulgaris Schard.*	Dellah	E	Leaves	Inf/Mac	1	[34]
	*Cucumis sativus* L.	Lkhiar	A, B, D, E, G-I, K, L, O-Q	Fruits	Raw/Mac/Pow/Jui	13	[19,21,24,25,26,27,29,30,31,32,34,35,37]
	*Cucumis melo var. flexuosus* L.	Feqous	A	Fruits	Raw	1	[29]
	*Cucurbita maxima Duchesne*	Garaa lhamra	E, H, L	Leaves/Seeds	Dec/Pow	3	[19,30,34]
	*Cucurbita pepo* L.	Takhsait/curjt	D, F, K, H, L, O, N, Q, R	Fruits	Raw/Dec/Coo	10	[19,21,23,24,27,30,32,39,45,51]
Cupressaceae	*Juniperus phoenicea* L.	Araar finiqui	A, D, E, K, L, O, R	Leaves/Aerial parts/Fruits/Barks	Pow/Dec Mac	8	[18,19,21,24,32,34,45,51]
	*Juniperus thurifera L*	Tawayt	O	Leaves	Dec	1	[24]
	*Juniperus oxycedrus* L.	L arâar chrini	E	Leaves	Mac	1	[34]
	*Tetraclinis articulata* (Vahl) Mast.	Araar	C, F, K, G-I, K, N, P, T, V, W	Leaves/Aerial parts/Fruits	Inf/Mac/Pow/Dec	13	[21,22,23,26,27,30,33,35,37,39,41,49,52]
Cynomoriaceae	*Cynomorium coccineum* L.	Tertut	L	Stems	Pow	1	[19]
Cyperaceae	*Bolboschoenus maritimus* (L.) *Palla*	Ssmar	K	Seeds	Dec	1	[21]
	*Cyperus longus* L.	Arouk, esaad	E	Roots	Mac	1	[34]
	*Cyperus rotundus* L.	Tara	L	Leaves	Pow	1	[19]
Dracaenaceae	*Dracaena draco* subsp. *ajgal* Benabid *&* Cuzin	Ajgal	Q	Stems/Leaves	Dec	1	[25]
Ephedraceae	*Ephedra alata* Decne.	Chdida	L	Leafy stem	Dec/Pow	1	[19]
	*Ephedra altissima* Desf.	Tougel argan	H, Q	Stems/Leaves/whole plant	Dec	2	[25,27]
	*Ephedra fragilis* Desf.	Amater	S	Leafy stem	Dec	1	[46]
Equisetaceae	*Equisetum ramosissimum Desf*	Dayl laawd	E	Stems	Dec	1	[34]
Ericaceae	*Arbutus unedo* L.	Sasnu/Barnnou	C-E, G, H, N, O	Leaves/Roots/Fruits	Dec/Inf	6	[23,24,27,34,35,41,51]
	*Vaccinium myrtillus* L.	Oleik	D	Fruits	Nd	1	[51]
Euphorbiaceae	*Euphorbia officinarum* subsp. *echinus* (Hook. f. & Coss.) Vindt	Tikiout/zakoum	E, K, L, O, Q	Fruits/Stems/Leaves	Mac/Dec/Pow/Jui	5	[19,20,21,25,34]
	*Euphorbia officinarum* L.	Tikiout/Daghmouss	D, H, Q, W	Stems/Leaves	Pow		[25,30,51,52]
	*Euphorbia peplis* L.	Hlliba	E, R	Whole plant	Inf	2	[34,45]
	*Euphorbia resinifera* O. Berg	Tikiwt	A, C, E, H, O, S	Leaves	A drop latex in a glass of water	7	[18,24,27,33,34,41,46]
	*Mercurialis annua* L.	Hurriga elmalssa	D, E, K, L	Leafy stem/Whole plant	Inf/Dec/Jui	4	[19,21,32,34]
	*Ricinus communis* L.	Awriwer/Lkharwaa	L	Seeds	Pou	1	[19]
Fagaceae	*Quercus coccifera* L.	Elqermez	K	Leaves	Dec	1	[21]
	*Quercus suber* L.	Belloute	A, B, D	Fruits	Dec/Raw	3	[29,31,32]
	*Quercus ilex* L.	Bellout, Kerrouch	C, E	Barks/Leaves	Dec	2	[33,34]
Gentianaceae	*Centaurium erythraea* Rafn	Qusset elhayya/Ahchlaf ntawrra	C, D, G, K, N, O	Flowering/Aerial parts	Inf/Dec/Pow	7	[21,23,24,33,35,41,51]
	*Centaurium spicatum* (L.) *Fritsch*	Gosset lhayya	E	Stems/Flowers	Inf	1	[34]
Geraniaceae	*Pelargonium odoratissimum* L.	M’atarcha	X	Leaves	Dec	1	[53]
	*Pelargonium roseum Willd.*	Laattercha	E	Leaves	Inf	1	[34]
Iridaceae	*Crocus sativus* L.	Zaafran lhor	D, E, G, H, L	Stigmas/Flowers	Inf/Dec/Mac	5	[19,30,32,34,35]
Juglandaceae	*Juglans regia* L.	Swak/Gargaa	C, D, E, G, K, L, O, S	Leaves/Barks/Seeds/Flowers	Inf/Dec/Raw	8	[19,21,24,32,33,34,35,46]
Juncaceae	*Juncus maritimus* Lam.	Ssemar	K, L	Fruits/Stems	Dec	2	[19,21]
Lamiaceae	*Ajuga iva* (L.) *Schreb.*	Timerna nzenkhad/Chndkoura	A, C-E, G-I, K, L, N, P, Q, S, T	Stems/Leaves/Whole plant	Pow/Dec/Inf	15	[18,19,21,22,23,25,26,27,33,34,35,37,40,41,46]
	*Ballota hirsuta Benth*	Merrou elhrami/Merrou	E, K	Leafy stem	Dec/Inf	2	[21,34]
	*Calamintha officinalis Moench.*	Manta	A, C, E, F, I	Aerial plants/Whole plant/Leaves/Stems/Flowers	Dec/Inf	5	[29,34,37,39,41]
	*Calamintha nepeta* subsp. *Spruneri* (*Boiss.*) *Nyman*	Nd	C	Nd	Nd	1	[33]
	*Calamintha alpina* L.	Fliyyo dial berr	D	Leaves	Dec	1	[28]
	*Clinopodium alpinum* (L.) *Kuntze*	Ziitra	D, L	Leaves	Dec	2	[19,28]
	*Clinopodium nepeta* subsp. *glandulosum* (*Req.*) *Govaerts*	Manta	N, T	Aerial parts	Inf/Dec	2	[22,23]
	*Lavandula angustifolia Mill*	Elkhzama zerqa/Elkhzama Fassiya	D, G, H, K, W	Aerial parts/Leafy stem	Inf/Dec/Pow	6	[21,30,32,35,51,52]
	*Lavandula dentata* L.	Timzeria/Lakhzama/Jaada	E, G, K, N, Q	Stems/Leaves/Whole plant	Dec/Pow/Inf/Raw/Pou	5	[21,23,25,34,35]
	*Lavandula maroccana Murb.*	Igazioen	E, Q, S	Stems/Leaves/Flowers	Dec/Inf	3	[25,34,46]
	*Lavandula multifida L*	Khilt lkheyl/Kohayla	E, G, L	Leaves/Inflorescence/Stems	Dec/Inf	3	[19,34,35]
	*Lavandula stoechas* L.	Imzeria/Tikenkert/Lhalhal	A, C, E, F, G, K, L, O, P, Q	Leaves/Flowers	Dec/Inf	10	[19,21,24,25,26,29,33,34,35,39]
	*Marrubium vulgare* L.	Mriwt/Ifzi	A, C, D, G-I, K, L, N-R, T, W	Leaves/Aerial parts	Dec/Inf/Pow	21	[18,19,20,21,22,23,24,25,26,27,28,29,30,32,33,35,37,41,45,51,52]
	*Mentha pulegium* L.	Fliou	A, C, D, F, G, K, L, O, Q, T	Leaves/Aerial parts	Dec/Inf	12	[18,19,21,22,24,25,28,29,32,33,35,39]
	*Mentha piperita* L.	Naanaa	D	Leaves/Aerial parts	Nd	1	[51]
	*Melissa officinalis* L.	Naanaa trunj	E	Leaves	Inf	1	[34]
	*Mentha spicata* L.	Nanaa/Liqama	D, E, K, L	Leaves/Leafy stem	Inf/Dec	4	[19,21,32,34]
	*Mentha suaveolens Ehrh.*	Mersita Timijja	D, E	Leaves/Whole plant	Inf	3	[28,32,34]
	*Ocimum basilicum* L.	Lahbaq	D, E, G, H, K, O	Stems/Whole plant/Leaves	Inf	6	[21,24,30,34,35,51]
	*Origanum compactum Benth.*	Azukenni/Zaater/Zaatar tadlawi	A-D, E, F, H, I, K, L, N, O, T	Stems/Leaves/Aerial parts	Dec/Inf/Pow/Mac	13	[19,21,22,23,24,29,30,31,33,34,37,39,51]
	*Origanum elongatum* (*Bonnet*) *Emb. &Maire*	Zaater	D, G	Leaves/Aerial plants	Inf	3	[28,32,35]
	*Origanum majorana* L.	Berdedouch	D, H, L	Leaves	Pow/Inf	4	[19,30,32,51]
	*Origanum vulgare* L.	Zaatar	C, P	Leaves	Inf	2	[26,33]
	*Rosmarinus officinalis* L.	Azir	A-I, K, L, N, O, Q, R, T, V, W	Leaves/Stems/Aerial plants	Pow/Dec/Inf/Mac	22	[18,19,21,22,23,24,25,28,29,30,31,32,33,34,35,37,39,41,45,49,51,52]
	*Salvia officinalis* L.	Salmia	A, C-E, G-I, K, L, O-T, V-X	Leaves/Aerial parts	Dec/Inf/Mac	24	[18,19,20,21,22,24,25,26,27,28,29,30,32,33,34,35,37,41,45,46,49,51,52,53]
	*Salvia hispanica* L.	Chia	D	Seeds	Nd	1	[51]
	*Teucrium polium* L.	Tawerart/Flyou lbour/jaaidia	A, E, H, Q, S	Leaves/Whole plant	Dec/Pow	5	[18,25,30,34,46]
	*Thymus broussonetii Boiss.*	Zietra	C, D, E	Stems/Leaves/Flowers	Inf/Mac/Dec	3	[28,34,41]
	*Thymus algeriensis Boiss. & Reut.*	Aduchen/Azukni/Zaitra	G, O	Stems/Leaves	Dec/Inf	2	[24,35]
	*Thymus maroccanus Ball.*	Tazoukennit	E, W	Leaves/Flowers	Inf/Mac	2	[34,52]
	*Thymus munbyanus Boiss. & Reut*	Aduchen/Azukni/Zaitra	O	Stems/Leaves	Dec/Inf	1	[24]
	*Thymus satureioides Coss.*	Asserkna/Ziitra	D, E, K, Q	Leaves	Inf/Dec/Pow/Mac	4	[21,25,32,34]
	*Thymus vulgaris* L.	Aduchen/Azukni/Zaitra	A, D-G, K, O, Q	Leaves/Aerial plants	Dec/Inf	8	[21,24,25,29,34,35,39,51]
	*Thymus zygis* L.	Aduchen/Azukni/Zaitra	G, O	Stems/Leaves	Dec/Inf	2	[24,35]
Lauraceae	*Cinnamomum cassia* (L.) *J. Presl*	Qarfa	A, C-E, H, K, O, T	Barks	Dec/Inf	8	[18,21,22,24,30,33,34,51]
	*Cinnamomum verum J. Presl*	Dar essini/Karfa	A, B, D, G, I, K, L, W	Barks	Mac/Inf/Dec/Pow	9	[19,21,28,29,31,32,35,37,52]
	*Laurus nobilis* L.	Ourak sidna moussa/Rand	B, D, E, F, I, H, K, P	Leaves	Inf/Dec	8	[21,26,30,31,34,37,39,51]
	*Persea americana Mill.*	Lavoca	A, D, H, L, O	Seeds/Fruits/Leaves	Pow/Ing/Raw	7	[18,19,20,28,30,32,51]
Leguminosae	*Acacia gummifera Willd.*	Telh	E	Roots	Dec	1	[34]
	*Acacia nilotica* (L.) Delile	Amur/Sllaha	L	Fruits	Pow	1	[19]
	*Acacia senegal* (L.) Willd.	Laalek	L	Gums	Pow	1	[19]
	*Acacia tortilis* (*Forssk.*) *Hayne*	Telh/Tadoute/Amrād	G, K, L, M	Roots/Fruits/Leaves	Dec/Pow	4	[19,21,35,54]
	*Acacia albida Delile*	Chok Telh	K, R	Roots	Dec	2	[21,45]
	*Anagyris foetida* L.	Ful gnawa	E, L	Seeds/Leaves	Pow/Inf	2	[19,34]
	*Arachis hypogaea* L.	Lgerta/Kawkaw	D, L	Seeds	Pow	2	[19,51]
	*Cassia absus* L.	El habba sawdae	E	Seeds	Pow	1	[34]
	*Cassia fistula* L.	ḫyār šambâr	G	Fruits	Dec	1	[35]
	*Ceratonia siliqua* L.	Tikida/Lkharoub	A, C-E, G-I, K, L, P, Q	Leaves/Seeds/Fruits	Dec/Inf/Pow/Raw	14	[19,21,25,26,27,28,29,32,33,34,35,37,41,51]
	*Cicer arietinum* L.	Lhemmes	A, D, E, H, L	Seeds	Dec/Pow/Inf	4	[19,27,29,34,51]
	*Cytisus battandieri Maire*	Akhamelel	C	Leaves	Dec	1	[41]
	*Glycine max* (L.) *Merr.*	Soja	A, C-H J, P, Q, S, W	Seeds	Mac/Raw/Inf/Dec/Pow	14	[17,25,26,27,29,30,32,34,35,39,41,46,51,52]
	*Glycyrrhiza glabra L*	Ark souss	D, E, F, I	Barks/Roots/Stems	Inf/Pow /Raw	6	[28,32,34,37,39,51]
	*Lupinus albus* L.	Tirms/Foul gnawa	A, C-E, G, H, K, L, O	Seeds	Pow/Inf/Dec	12	[18,19,20,21,27,29,32,33,34,35,41,51]
	*Lupinus angustifolius* L.	Ibawn dekouk	G, K, Q, S	Seeds	Pow /Dec	4	[21,25,35,46]
	*Lupinus luteus* L.	Kikel/Semqala	E, K	Seeds	Dec	2	[21,34]
	*Lupinus pilosus* L.	Rjel Djaja	R	Seeds	Inf	1	[45]
	*Medicago sativa* L.	Fassa	B, D, E, K, H, I, L, O, P	Aerial parts/Seeds/Leaves	Inf/Mac/Coo/Pow	9	[19,21,24,26,27,31,34,37,51]
	*Ononis natrix* L.	Hennet reg	L	Leaves	Dec	1	[19]
	*Ononis tournefortii Coss.*	Afezdad	L	Leaves	Dec	1	[19]
	*Phaseolus aureus Roxb.*	Soja	R	Seeds	Dec	1	[45]
	*Phaseolus vulgaris* L.	Lubya	D, E, K, L, O, R	Fruits/Seeds	Dec/Pow/Jui/Raw/Ing	7	[19,20,21,24,32,34,45]
	*Retama monosperma* (L.) *Boiss.*	Rtam	E	Roots/Leaves	Dec/Inf	1	[34]
	*Retama raetam* (*Forssk.*) *Webb*	Rtam/Allug	G, K	Roots/Leaves/Aerial plants	Dec/Pow	2	[21,35]
	*Retama sphaerocarpa* (L.) *Boiss.*	Rtem	J	Roots	Dec	1	[17]
	*Senna alexandrina Mill.*	Senameki	D	Leaves	Nd	1	[51]
	*Trigonella foenum-graecum* L.	Lhelba/Tifidas	A-L, N, O, P, Q, S, T, W	Seeds	Dec/Inf/Mac/Pow	25	[17,18,19,20,21,22,23,24,25,26,27,28,29,30,31,32,33,34,35,37,39,41,46,51,52]
	*Vicia faba* L.	Ful/Foul	A, D, L	Seeds	Pow	3	[19,29,32]
	*Vicia sativa* L.	Ayn larnab	L	Seeds	Pow	1	[19]
	*Vigna radiata* (L.) *R.Wilczek*	Soja	L	Seeds	Pow	1	[19]
	*Vigna unguiculata* (L.) *Walp*	Ful gnawa	G, K	Seeds	Dec/Pow/Mac	2	[21,35]
	*Urginea maritima* (L.) *Baker*	Bssallansal	C	Leaves	Dec	1	[41]
Linaceae	*Linum usitatissimum* L.	Zariat elkattan	A-I, K, L, O, Q, R, T	Seeds	Dec/Pow/Inf	17	[19,21,22,24,25,28,29,30,31,32,33,34,35,37,39,45,51]
Lythraceae	*Lawsonia inermis* L.	Lhenna	F, K, G	Leaves	Dec/Cat/Pow/Inf	3	[21,35,39]
	*Punica granatum* L.	Rman	A-G, I-L, O, Q, T	Pericarps/Barks/Fruits/Leaves	Dec/Inf/Pow	16	[17,18,19,21,22,24,25,29,31,32,33,34,35,37,39,51]
Malvaceae	*Abelmoschus esculentus* (L.) *Moench*	Lmloukhia	B, D, E, O	Fruits/Flowers	Mac/Inf/Raw	5	[24,28,31,32,34]
	*Hibiscus sabdariffa* L.	Karkadi/Bissam	C-E, K, L, S	Calyces/Leaves/Flowers	Inf	6	[19,21,33,34,46,51]
Moraceae	*Ficus abelii Miq*	Karmous, Chriha	R	Leaves	Dec	1	[45]
	*Ficus carica* L.	Tazart/Lkarmous/Karma/chriha/Elbakur	A-K, O, Q, R, T	Fruits/Leaves	Dec/Inf/Raw/Mac	18	[17,21,22,24,25,27,29,30,31,32,33,34,35,37,39,41,45,51]
	*Ficus dottata Gasp.*	Karmous, Chriha	R	Fruits	Other	1	[45]
	*Morus alba* L.	Tut lbari	A, D, G, K, O, R	Leaves	Inf	6	[18,21,24,35,45,51]
	*Morus nigra* L.	Šejrat t-tūt	G	Leaves	Inf	1	[35]
Moringaceae	*Moringa oleifera Lam.*	Moringa	D	Leaves	Nd	1	[51]
Musaceae	*Musa paradisiaca* L.	Banan	L	Leaves	Dec	1	[19]
Myristicaceae	*Myristica fragrans Houtt.*	Lgouza	C, Q	Seeds	Pow	2	[25,41]
Myrtaceae	*Eucalyptus camaldulensis Dehnh.*	Calitus	L	Leaves	Dec	1	[19]
	*Eucalyptus globulus Labill.*	Calitus	A, C-E-I, K, N, O, T	Leaves/Fruits/Stems	Dec/Inf/Pow	13	[21,22,23,24,27,29,33,34,35,37,39,41,51]
	*Eugenia caryophyllata Thunb*	Qronfel	C-E	Cloves/Leaves/Flowers	Mac/Inf/Pow/Dec	4	[33,34,41,51]
	*Jasminum fruticans* L.	Yasmin	E	Leaves/Flowers	Mac/Inf	1	[34]
	*Myrtus communis* L.	Rihane	A, C-K, N, O	Leaves/Fruits/Flowers	Dec/Inf/Mas/Pow	14	[17,21,23,24,27,29,30,32,33,34,35,37,39,41]
	*Syzygium aromaticum* (L.) *Merr. &* L.* M. Perry*	Kranfal	A, D, K, H, I, L, N, Q	Fruits/Cloves/Seeds	Inf/Dec/Pow/Mac	9	[18,19,21,23,25,27,28,32,37]
Nitrariaceae	*Peganum harmala* L.	Lharmel	C, E, G, I, H, J, K, O, T	Seeds	Inf/Pow/Mac	9	[17,21,22,24,30,34,35,37,41]
Oleaceae	*Fraxinus angustifolia Vahl*	Touzalt	O	Leaves	Inf	1	[24]
	*Fraxinus excelsior var.acuminata Schur*	Lsān Eṭ-Ṭîr/Lsān L’uṣfūr/Ḥebb Derdār	G	Fruits/Stems/Barks	Dec/Inf/Pow	1	[35]
	*Olea europaea* L.	Jbouj/Azmour/Zitoun	A-H, J, K, L, O, P, Q, S, T, W, X	Leaves/Fruits/Flowers	Dec/Inf/Mac/Pow/Oil	24	[17,18,19,20,21,22,24,25,26,27,28,29,30,31,32,33,34,35,39,40,46,48,51,52,53]
	*Olea europaea* subsp. *maroccana* (*Greuter & Burdet*)	Zitūn/Zebbūj	G	Leaves/Fruits	Dec/Oil	1	[35]
	*Olea europea* subsp. *europaea var. sylvestris* (*Mill*) *Lehr*,	Jebbouj	I	Leaves	Dec	1	[37]
	*Olea oleaster Hoffm.& Link.*	Zabbouj	E	Leaves/Flowers	Inf	1	[34]
Papaveraceae	*Fumaria officinalis* L.	Hachichat assebyane	E, K, R	Roots/Leaves	Dec/Inf	3	[21,34,45]
	*Papaver rhoeas* L.	Belaaman	A, C, H, I, Q, S	Seeds	Pow	6	[25,27,29,37,41,46]
	*Plantago ovata Forssk.*	Katouna	C, D	Seeds	Inf	2	[41,51]
Pedaliaceae	*Sesamum indicum* L.	Janjlan	A, D-J, L, N, Q, W	Seeds	Pow/Inf/Dec	12	[17,19,23,25,27,29,32,34,35,37,39,52]
Plantaginaceae	*Globularia alypum* L.	Ayen lerneb/Taselgha	A, C, E-H, K, L, O, S, T	Flowers/Leaves/Stems	Inf/Dec/Pou	12	[18,19,20,21,22,24,30,33,34,35,39,46]
	*Globularia repens Lam.*	Ain lernab	P	Leaves	Dec	1	[26]
Plumbaginaceae	*Limonium sinuatum* (L.) *Mill.*	Lgarsa	L	Leaves	Dec	1	[19]
Poaceae	*Avena sativa* L.	Khortal	D, E, K, O	Seeds	Pow/Inf/Dec	5	[21,24,32,34,51]
	*Avena sterilis* L.	Waskone/Khortal	E, S	Seeds	Pow/Dec	2	[34,46]
	*Castellia tuberculosa* (*Moris*) *Bor*	Zwan lmkarkeb	E, K	Seeds	Dec	2	[21,34]
	*Cynodon dactylon* (L.) *Pers.*	Njem	L	Roots	Dec	1	[19]
	*Hordeum vulgare* L.	Chair/Zraa	D-F, K, L, Q	Aerial parts/Seeds/Whole plant	Inf/Pow /Mac/Dec	7	[19,21,25,32,34,39,51]
	*Lolium perenne* L.	Eziwane/Zouane	D, E, S, W	Seeds	Dec/Inf	4	[34,46,51,52]
	*Lolium multiflorum Lam.*	Zwane	A	Seeds	Pow	1	[29]
	*Lolium rigidum Gaudin*	Zwan	D	Seeds	Inf/Ing	1	[32]
	*Panicum miliaceum* L.	Tafssout	E, K	Seeds	Dec	2	[21,34]
	*Panicum turgidum Forssk.*	Umm rekba	L	Stems	Dec/Pow	1	[19]
	*Pennisetum glaucum* (L.) *R.Br.*	Illan	D, K, L, Q	Seeds	Inf/Pow	4	[19,21,25,51]
	*Phalaris canariensis* L.	Zouan	E, K, H, N, O, Q	Seeds/Fruits	Pow/Inf/Dec	7	[20,21,23,24,25,27,34]
	*Phalaris paradoxa* L.	Zwan/Senbūlt l-fār/Tigurramin	G	Seeds	Pow/Dec	1	[35]
	*Polypogon monspeliensis* (L.) *Desf*	Tugga	L	Fruits	Raw	1	[19]
	*Sorghum bicolor* (L.) *Moench*	Bachna	O, T	Seeds	Inf/Dec	2	[22,24]
	*Triticum durum Desf.*	Zraa/Lkamh	D, E, F, K	Seeds	Dec/Inf	4	[21,34,39,51]
	*Triticum aestivum* L.	Zraa	D, F	Seeds	Mac	2	[32,39]
	*Triticum turgidum* L.	Zraa	C	Nd	Nd	1	[33]
	*Zea mays* L.	Lahyat Adra	C, H, N, S	Stigmas	Pow	4	[23,27,33,46]
Polygonaceae	*Emex spinosa* (L.) *Campd.*	Lhenzab	L	Leaves/Bulbs	Pow	1	[19]
	*Portulaca oleracea* L.	Rejla	E, K, Q, R, S	Aerial parts/Whole plant	Dec/Coo	5	[21,25,34,45,46]
Ranunculaceae	*Nigella Sativa* L.	Sanouj	A-L, N, O, Q, S, T, W	Seeds/Fruits	Inf/Dec/Pow/Ing	40	[17,18,19,20,21,22,23,24,25,27,28,29,30,31,32,33,34,35,37,39,41,46,51,52]
Resedaceae	*Reseda lanceolata Lag.*	Rġūwa/L-Ḫrūf/Islīḫ	G	Seeds/Leaves	Dec/Pow/Inf	1	[35]
Rhamnaceae	*Ziziphus lotus* (L.) *Lam.*	Nbeg/Azouggar/ssdra	A-D, E, G-L, Q, S, T	Leaves/Fruits/Roots	Dec/Pow/Inf	17	[17,18,19,21,22,25,27,29,30,31,33,34,35,37,41,46,51]
	*Ziziphus jujube Mill*	Zafzouf	C	Leaves	Dec	1	[41]
Rosaceae	*Cydonia oblonga Mill.*	Sferjel	J	Fruits	Raw	1	[17]
	*Chaenomeles sinensis* (*Dum.Cours.*) *Koehne*	Sferjel	L	Roots	Dec	1	[19]
	*Crataegus monogyna Jacq.*	Za’zûr/Zu’rûr	C	Nd	Nd	1	[33]
	*Eriobotrya japonica* (*Thunb.*) *Lindl.*	Mzah	D, F, H, O, T	Leaves/Fruits	Inf/Dec/Raw/Jui	5	[22,24,30,32,39]
	*Fragaria vesca* L.	Fraiz berri	C	Fruits	Raw	1	[33]
	*Malus communis* (L.) *Poir.*	Etefah	D, E, G, S, R	Fruits	Jui/Raw/Vin	4	[32,35,45,46,48]
	*Prunus armeniaca* L.	Luz elhar	E, K	Seeds	Dec	2	[21,34]
	*Prunus dulcis* (*Mill.*) *D.A. Webb*	Louz imrzig/Louz morr	A-G, J, K, L, N, Q, S, T	Seeds/Leaves/Fruits	Raw/Dec/Pow	16	[17,19,21,22,23,25,28,29,31,32,33,34,35,39,41,46,51]
	*Prunus cerasus* L.	Red cherry	D, F	Seeds/Fruits	Jui/Raw	2	[39,51]
	*Rubus fruticosus var. vulgaris* (*Weihe & Nees*	Laalig/Toute	D, K	Leaves	Pow/Inf	2	[21,32]
	*Rubus fruticosus var. ulmifolius*, (*Schott*)	Laallik/Tabgha	E	Leaves/Fruits	Inf	1	[34]
Rubiaceae	*Rubia tinctorum* L.	Fowwa	L	Roots	Pow	1	[19]
	*Coffea arabica* L.	Qahwa	D, C	Seeds	Inf/Dec	3	[32,33,51]
Rutaceae	*Citrus medica var. limon* L.	Lhamed beldî	D, E, G, K	Fruits/Flowers/Leaves	Jui/Inf/Mac/Raw/Dec	5	[21,32,34,35,51]
	*Citrus paradisi Macfad.*	Pamblamus/Renj	D-F, H, K	Fruits	Jui/Raw	5	[21,30,32,34,39]
	*Citrus sinensis* (L.) *Osbeck*	Limun	F, L, P	Fruits	Raw /Jui	3	[19,26,39]
	*Citrus aurantium* L.	Larenj/Zenbue/trunj	A, C, E, J, H, K, L, N, O	Leaves/Fruits/Flowers	Jui/Inf/Dec	9	[17,18,19,20,21,23,30,34,41]
	*Ruta graveolens* L.	Lfijel	E, K, L	Roots	Dec/Inf	3	[19,21,34]
	*Ruta chalepensis* L.	Fjīla/L-Fījel/Āwermi	G	Aerial parts	Dec/Pow	1	[35]
	*Ruta montana* L.	Lfijel/Iwermi	A, E, J, K, N, O, T	Stems/Leaves	Dec/Inf/Pow	7	[17,18,21,22,23,24,34]
Salicaceae	*Salix alba* L.	Salef lma	D, E, J	Leaves	Dec	3	[17,48,51]
Salvadoraceae	*Salvadora persica* L.	Siwak	D	Barks	Mac	1	[32]
Santalaceae	*Viscum album L*	Lenjbar	T	Seeds	Inf	1	[22]
Sapotaceae	*Argania spinosa* (L.) *Skeels*	Argan	B-D, F-H, K, L, O, Q, S, T	Seeds/Fruits/Leaves	Raw /Pow/Ing/Oil	15	[19,20,21,22,24,25,28,30,31,32,33,35,39,46,51]
Schisandraceae	*Illicium verum Hook. f.*	Badiana	K	Fruits	Dec	1	[21]
Solanaceae	*Capsicum annuum* L.	Felfel Hârr/soudania	C, E, L, N, O	Fruits	Raw	5	[19,23,24,33,34]
	*Datura stramonium* L.	Sdag jmel/Metal	L	Seeds	Dec	1	[19]
	*Lycopersicon esculentum Mill.*	Maticha	E, K, L	Fruits	Raw	3	[19,21,34]
	*Nicotiana tabacum* L.	Nefha	N	Leaves	Dec	1	[23]
	*Solanum melongena* L.	Bdenjal	D	Fruits	Raw/Dec/Inf	1	[32]
	*Withania frutescens* (L.) *Pauquy*	Tirnet	E	Leaves	Inf	1	[34]
Taxaceae	*Taxus baccata* L.	Guelguem/Aguelguimt	E, K	Roots	Dec	2	[21,34]
Theaceae	*Camellia sinensis* (L.) *Kuntze*	Attay	D, E, G-I, K, L, P, Q, T	Leaves/Seeds	Inf/Dec	11	[19,21,22,25,26,27,32,34,35,37,51]
Thymelaeaceae	*Thymelaea hirsuta* (L.) *Endl.*	Metnan	E, G, K	Leafy stem/Leaves	Pow/Inf	3	[21,34,35]
	*Thymelaea tartonraira* (L.) *All.*	Talazazt	J	Leaves	Dec	1	[17]
	*Thymelaea virgata* (*Desf.*) *Endl.*	Metnan	E, K	Leafy stem	Dec	2	[21,34]
	*Aquilaria malaccensis Lam*	Taghriste	D, W	Barks	Inf/Dec/Mac	2	[32,52]
Urticaceae	*Urtica dioica* L.	Taznagt/Tigzenin/Lhriga	C, D, G, H, J, K, N, Q, S, T	Stems/Leaves	Dec/Inf	11	[17,21,22,23,25,27,30,35,41,46,51]
	*Urtica pilulifera* L.	Hurriga/Tisrakmaz	O	Leaves	Dec	1	[24]
	*Urtica urens* L.	Tikzint	E, I	Leaves/Stems	Pow/Dec	2	[34,37]
	*Urtica membranacea Poir. ex Savigny*	Ḥurrayga/Malssā	G	Leaves/Aerial parts	Pou/Dec	1	[35]
Valerianaceae	*Nardostachys jatamansi* (*D. Don*) *DC.*	Underground part	W	Underground parts	Inf	1	[52]
Verbenaceae	*Aloysia citriodora Palau*	Alwiza/Louiza	E, D, L, N, O, T	Leaves	Dec/Inf	6	[19,20,22,23,32,34]
	*Verbena officinalis* L.	Alwiza	B, D, I, H	Leaves	Dec/Inf	4	[28,30,31,37]
Vitaceae	*Vitis vinifera* L.	Dalya/Zbib/Kerma/Adilite	E, J, K, L	Leaves	Dec	4	[17,19,21,34]
Xanthorrhoeaceae	*Asphodelus microcarpus Salzm. & Viv.*	Lberwag/blaluz/Tazia	E, K, L	Tubers	Raw/Dec	3	[19,21,34]
	*Asphodelus tenuifolius Cav.*	Lehyat al aatrus/Tazya/Lberiwiga	K	Leaves	Dec	1	[21]
Zingiberaceae	*Zingiber officinale Roscoe.*	Sekinjbir	A, C-E, H-J, L, N, T	Rhizomes	Dec/Inf/Pow /Mac	12	[17,19,22,23,28,29,30,32,33,34,37,51]
	*Curcuma longa* L.	Kharqum	D, I	Stems/Rhizomes	Inf	4	[28,32,37,51]
Zygophyllaceae	*Tetraena gaetula* (*Emb. & Maire*) *Beier & Thulin*	Aagaia	A, J, K, L, N, O, Q	Leaves/Roots/Seeds	Pow/Inf/Dec	7	[17,18,19,21,23,24,25]
	*Zygophyllum gaetulum Emb. &Maire*	Aagaya	A, G	Aerial parts/Leaves	Dec/Inf	2	[29,35]

**Regions**: **A**, Fez; Meknes. **B**, Ksar Elkebir. **C**, Taza. **D**, Rabat-Sale-Kenitra. **E**, High Atlas Central. **F**, Tangier-Tetouan. **G**, Safi and Essaouira. **H**, Beni-Mellal-Khenifra. **I**, Casablanca-Settat. **J**, Errachidia. **K**, Al Haouz-Rhamna. **L**, Tan-Tan. **M**, Laayoune Boujdour Sakia El Hamra. **N**, Izarene. **O**, Middle Atlas. P, Sidi Slimane. Q, Chtouka Ait Baha and Tiznit. R, Moroccan Rif. S, Taroudant. T, Oriental Morocco (Oujda). V, Central Plateau. W, Guelmim. X, Agadir. Y, Ouezzane. **Mode(s) of use**: **Dec**: Decoction. **Pow**: Powder. **Mac**: Maceration. **Inf**: Infusion. **Ing**: Ingestion. **Jui**: Juice. **Fum**: Fumigation. **Coo**: Cooking/Cooked. **Per**: Perfusion. **Pou**: Poultice. **Cat**: Cataplasm. **Mas**: Mastication. **Vin**: Vinegar.

**Figure 4:**
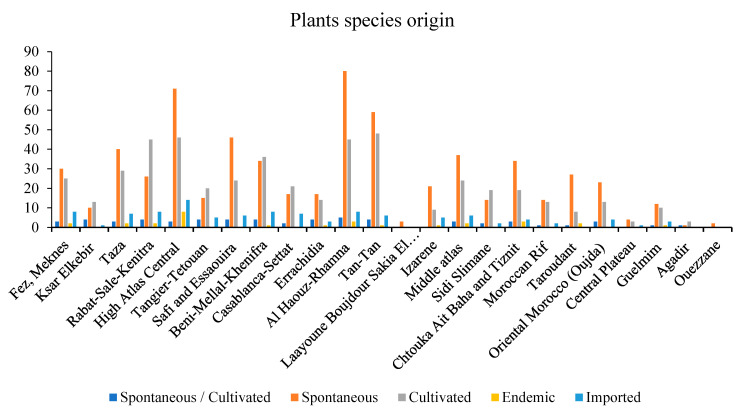
The distribution of plants species origin per Moroccan regions.

The majority of Moroccan medicinal plants reported during the last two centuries to treat diabetes grow spontaneously (56%), while a significant portion are cultivated (34%), some are imported (5%), some are endemic and some are either spontaneous or cultivated (3%) (Table 2, Figure 4).

**Figure 5:**
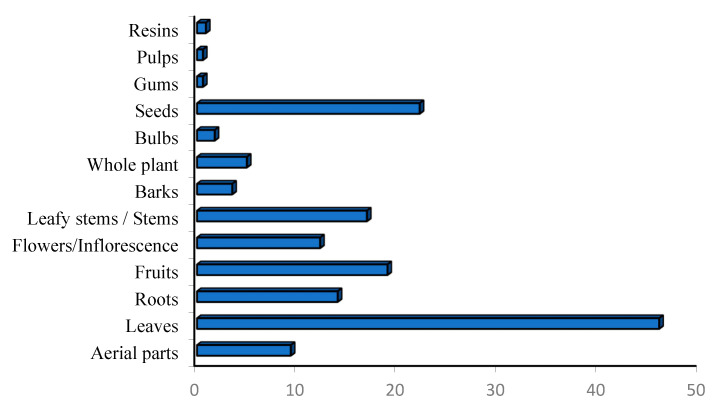
The distribution of the percentage of different parts used for diabetes management in Morocco.

**Figure 6:**
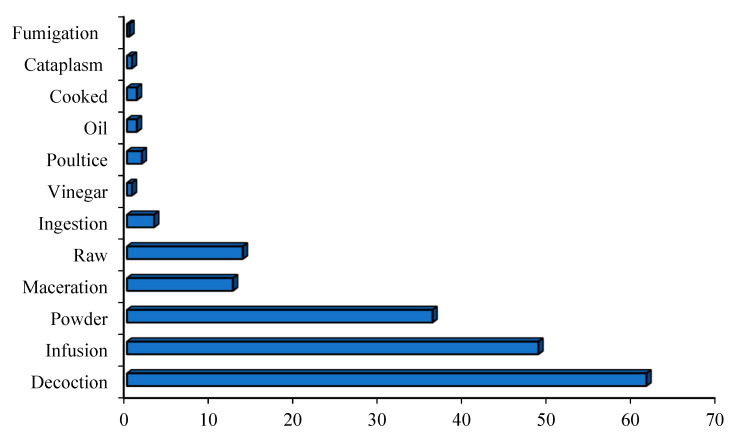
The distribution of the percentage of different preparation methods used for diabetes management in Morocco.

The survey of the ethnobotanical literature showed that different plant parts are used to treat diabetes in Morocco, such as aerial parts (10%), leaves (47%), roots (14%), fruits (19%), flowers/inflorescence (12%), leafy stems/stems (17%), barks (4%), whole plant (5%), bulbs (2%), seeds (22%), resins (1%) and gums (0.6%) (Figure 5). Moreover, different preparation methods are used to treat diabetes in Morocco, such as decoction (62%), infusion (49%), powder (36%), maceration (13%), raw (14%), ingestion (3%), vinegar (0.6%), poultice (2%), oil (1%), cooked (1%), cataplasm (0.6%), fumigation (0.3%), etc. (Figure 6).

Moroccan traditional medicine incorporates a wide array of plant species for managing diabetes. While some plants are well-documented in the scientific literature, others remain under-studied or unknown. This categorization helps highlight the need for further research, especially on lesser-known and unknown species, to ensure their safe and effective use in diabetes management.

#### 3.1.1. Antidiabetic Plants Well-Known in Pharmacological Literature of Diabetes

Several plant species have been extensively studied for their antidiabetic properties. They are frequently used in traditional medicine and supported by scientific studies. Among 344 plants species, 100 species belonging to 45 families are considered well-known antidiabetic plants. The most represented families are Lamiaceae, Asteraceae, Leguminosae, and Poaceae. The Lamiaceae family is the most frequently used in traditional Moroccan medicine. Fourteen species were reported as used in traditional antidiabetic treatment in the literature, including *Ajuga iva*, *Marrubium vulgare*, *Mentha piperita*, *Melissa officinalis*, *Mentha spicata*, *Ocimum basilicum*, *Origanum majorana*, *Rosmarinus officinalis*, *Salvia officinalis*, *Salvia hispanica*, *Teucrium polium*, *Thymus satureioides*, *Thymus vulgaris*, and *Thymus zygis.* The leaves of these medicinal plants are the most commonly used parts to treat diabetes in Morocco. The modes of use vary by region, but infusion and decoction are the most common forms [18,19,20,21,22,23,24,25,26,27,28,29,30,31,32,33,34,35,37,39,40,41,45,46,49,51,52,53].

The Leguminosae family has been reported as the second most rich source of Moroccan traditional species used for diabetes management. This family includes thirteen medicinal species, such as *Acacia nilotica*, *Acacia albida*, *Anagyris foetida*, *Cassia fistula*, *Cicer arietinum*, *Glycine max*, *Glycyrrhiza glabra*, *Lupinus albus*, *Medicago sativa*, *Phaseolus vulgaris*, *Trigonella foenum-graecum*, *Vigna radiata*, and *Vigna unguiculata*. Different parts of these plants, such as seeds, roots, fruits, leaves, stems, aerial parts and barks, are used. The mode of preparation differs by region, but most patients use species from this family after decoction, infusion, maceration, or as a powder [17,18,19,20,21,22,23,24,25,26,27,28,29,30,31,32,33,34,35,37,39,41,45,46,51,52]. Medicinal plants belonging to the Asteraceae family have also been highlighted as a rich source of remedies used for diabetes management. Seven well-known antidiabetic plants belonging to this family are reported, including *Phoenix dactylifera*, *Artemisia herba-alba Asso*, *Cichorium intybus*, *Helianthus annuus*, *Matricaria chamomilla*, *Stevia rebaudiana*, and *Silybum marianum*. The parts used are mainly leaves and roots, prepared by infusion or decoction, or consumed as a powder [17,18,19,20,21,22,23,25,26,27,28,29,30,32,33,34,35,37,39,41,44,45,46,48,51,52].

Another rich family, Poaceae, is reported as having antidiabetic agents in different Moroccan regions. Five species are included in this family, such as *Cynodon dactylon*, *Hordeum vulgare*, *Pennisetum glaucum*, *Sorghum bicolor*, and *Triticum aestivum.* The seeds of the three last species are prepared by infusion, decoction, and maceration, or taken as a powder. Meanwhile, different parts (aerial parts, seeds, and the whole plant) of *Hordeum vulgare* are prepared using different methods, whereas the roots of *Cynodon dactylon* are used after decoction [19,21,22,24,25,32,34,39,51]. The Cucurbitaceae, Lauraceae, and Myrtaceae families (four species each) have been reported as antidiabetic medicinal plants by Moroccan patients. Plants belonging to the Cucurbitaceae family include *Citrullus colocynthis*, *Cucumis sativus*, *Cucurbita maxima*, and *Cucurbita pepo*. Their fruits are prepared using various methods such as raw, decoction, powder, juice, ingestion, maceration, cooking or cataplasm [18,19,21,23,24,25,26,27,28,29,30,31,32,33,34,35,37,39,45,46,51,54]. Four species have also been reported in the Lauraceae family as well-known antidiabetic plants, including *Cinnamomum cassia*, *Cinnamomum verum*, *Laurus nobilis*, and *Persea americana*. Different parts of these species, such as barks, leaves, seeds and fruits, are used by diabetic patients. The preparation method most commonly used by these patients is infusion [18,19,20,21,22,24,26,28,29,30,31,32,33,34,35,37,39,51,52]. Four species, including *Eucalyptus camaldulensis*, *Eucalyptus globulus*, *Myrtus communis*, and *Syzygium aromaticum*, have also been reported in the Myrtaceae family as well-known antidiabetic species used by Moroccan patients from different regions. The leaves of these species are prepared using different methods to treat diabetes [17,18,19,20,21,22,23,24,25,27,28,29,30,32,33,34,35,37,39,41,51]. Plants from the Brassicaceae family have been reported in the treatment of diabetes in Morocco for a long time. The plants used are *Brassica oleracea*, *Brassica rapa*, and *Lepidium sativum*. The aerial parts, fruits, roots, leaves and seeds of these species are prepared in various ways by Moroccan diabetic patients [17,18,19,21,24,26,27,28,29,30,31,32,33,34,35,37,39,41,45,48,51,52].

Ten families, each presented by two plant species, are pillars of traditional Moroccan medicine in the management of diabetes. *Allium cepa* and *Allium sativum* from the Alliaceae family are globally recognized for their antidiabetic properties. The bulbs are typically consumed raw or cooked. Additionally, they are prepared via decoction or maceration for medicinal use. Garlic can also be consumed as a powder or supplement in the form of capsules [17,18,19,20,21,22,23,24,25,26,27,28,29,30,31,32,33,34,35,36,37,39,51]. The leaves of *Calotropis procera* and *Nerium oleander* (Apocynaceae) are used. Traditionally, *N. oleander* leaves are prepared as a decoction, though careful dosage is necessary due to the plant’s toxicity. *Calotropis procera* is used as a powder for its antidiabetic properties [17,18,19,21,22,23,25,26,27,32,33,34,35,36,37,39,41,44,46,48,50,51,52]. The Capparaceae family is also represented by two species, *Capparis decoctionidua* and *Capparis spinosa*. The fruits of the first species are consumed as a powder, whereas different parts of the second species are prepared after decoction and infusion, or as a powder [17,18,19,21,23,24,34,35,41,46,51,52]. The Ericaceae family, with *Arbutus unedo* and *Vaccinium myrtillus*, offers its leaves and fruits, used in infusion or decoction [23,24,27,34,35,41,51]. The Lythraceae family includes *Lawsonia inermis* and *Punica granatum*, with leaves and fruit rinds used via different methods [17,18,19,21,22,24,25,29,31,32,33,34,35,37,39,51]. In the Malvaceae family, *Hibiscus sabdariffa* calyces are consumed as a tea, and *Abelmoschus esculentus* fruits and flowers are used in infusion and maceration or as a powder [19,21,24,28,31,32,33,34,46,51]. The Moraceae family, with *Ficus carica* and *Morus alba*, provides its fruits and leaves, which are used in infusion [17,18,21,22,24,25,27,29,30,31,32,33,34,35,37,39,41,45,51]. The Rosaceae family includes *Cydonia oblonga* fruits and *Eriobotrya japonica* leaves and fruits, typically used raw or prepared by infusion or decoction [17,22,24,30,32,39]. The Rutaceae family, represented by *Citrus sinensis* and *Citrus aurantium*, is used in combination with lemon juice, and *C. aurantium* leaves are used in infusion or decoction [17,18,19,20,21,23,26,30,34,39,41]. Finally, the Zingiberaceae family, featuring *Zingiber officinale* and *Curcuma longa*, provides its rhizomes used in powder or decoction, often added to food for their antidiabetic effects [17,19,22,23,28,29,30,32,33,34,37,51].

Several plant families are represented by a single species traditionally known for its antidiabetic properties. The Aloeaceae family is represented by *Aloe vera*; the gel from leaves is consumed raw or as a powder [19,21,22,30,32,39,51]. In the Anacardiaceae family, *Pistacia atlantica* fruits are used in infusion or decoction [25,41,44]. The Apiaceae family includes *Foeniculum vulgare*, with seeds and fruits consumed as an infusion or incorporated into meals [17,18,19,21,25,26,27,29,30,32,34,35,37,41,51,52,53]. The **Cactaceae** family is represented by *Opuntia ficus-indica*, where the stems, roots, flowers, seeds and fruit are consumed raw or prepared by decoction and infusion or as an oil [17,19,20,21,22,24,25,26,27,29,30,31,32,33,35,39,41,51]. In the Cannabaceae family, *Cannabis sativa* seeds and leaves are consumed as a powder by diabetic patients from Tangier and Tetouan regions [39]. The Convolvulaceae family includes *Ipomoea batatas*; its roots are used in dietary preparations [29]. In the Cyperaceae family, *Cyperus rotundus* leaves are consumed by diabetic patients from Tan-Tan as a powder [19]. The Euphorbiaceae family is represented by *Ricinus communis*, with the seeds prepared in a poultice [19]. The Gentianaceae family includes *Centaurium erythraea*, the whole plant of whch is used in decoction or infusion [21,23,24,33,35,41,51]. In the Iridaceae family, *Crocus sativus* stigmas and flowers are prepared as an infusion, or via decoction or maceration [19,30,32,34,35]. The Juglandaceae family is represented by *Juglans regia*, with leaves used in decoction [19,21,24,32,33,34,35,46]. The Linaceae family includes *Linum usitatissimum*, the seeds of which are consumed via infusion and decoction, or in food [19,21,22,24,25,28,29,30,31,32,33,34,35,37,39,45,51]. The Moringaceae family is represented by *Moringa oleifera*, with leaves prepared as teas or powder [51]. The leaves of *Musa paradisiaca* (Musaceae) are used in decoction or cooked dishes [19]. In the Myristicaceae family, *Myristica fragrans* seeds are consumed in powdered form [25,41]. The seeds of *Peganum harmala* (Nitrariaceae) are prepared using various methods [17,21,22,24,30,34,35,37,41]. The Oleaceae family includes *Olea europaea*, with leaves prepared via infusion [17,18,19,20,21,22,24,25,26,27,28,29,30,31,32,33,34,35,39,40,46,48,51,52,53]. The Polygonaceae family is represented by *Portulaca oleracea*, the whole plants of which are used in decoction [21,25,34,45,46]. In the Ranunculaceae family, *Nigella sativa* seeds are consumed powdered, after decoction via ingestion or in infusions [17,18,19,20,21,22,23,24,25,27,28,29,30,31,32,33,34,35,37,39,41,46,51,52]. The roasted seeds of *Coffea arabica* (Rubiaceae) are used in infusion or decoction [32,33,51]. The seeds of *Viscum album* (Santalaceae) are also used in infusion [22]. The Sapotaceae family includes *Argania spinosa*, with seeds, fruits and leaves used crude, after ingestion, or as an oil in cooking [19,20,21,22,24,25,28,30,31,32,33,35,39,46,51]. In the Solanaceae family, *Datura stramonium* seeds are used in decoction [19]. The Theaceae family includes *Camellia sinensis*, with leaves and seeds prepared as infusions or decoctions [19,21,22,25,26,27,32,34,35,37,51]. The Urticaceae family is represented by *Urtica dioica*, the leaves of which are prepared by decoction or infusion [17,21,22,23,25,27,30,35,41,46,51]. Finally, the leaves of *Vitis vinifera* (Vitaceae) are used in decoction [17,19,21,34].

#### 3.1.2. Antidiabetic Plants Little Known in Pharmacological Literature on Diabetes

The exploration of little-known antidiabetic plants is gaining momentum as traditional herbal remedies are being increasingly recognized for their potential benefits in managing diabetes. This category encompasses 124 species, and their uses are recognized in Moroccan traditional medicine.

The Asteraceae family is notable for its diverse members. Among its eighteen species are *Anacyclus pyrethrum*, *Artemisia absinthium*, *Artemisia arborescens*, *Artemisia campestris*, *Artemisia mesatlantica*, *Atractylis gummifera*, *Calendula arvensis*, *Chamaemelum nobile*, *Chrysanthemum coronarium*, *Cynara cardunculus*, *Dittrichia viscosa*, *Lactuca sativa*, *Pallenis spinose*, *Saussurea costus*, *Scorzonera undulata*, *Sonchus arvensis*, *Sonchus asper*, and *Warionia saharae.* Different parts of these plants are used via infusion or decoction by diabetic patients from different Moroccan regions [17,18,19,20,21,22,23,24,25,26,27,29,30,31,32,33,34,35,37,38,39,41,45,46,47,48,49,51,52].

Within the Apiaceae family, fourteen species present significant antidiabetic potential. This family includes *Ammodaucus leucotrichus*, *Ammi visnaga*, *Apium graveolens*, *Carum carvi*, *Coriandrum sativum*, *Cuminum cyminum*, *Daucus carota*, *Ferula communis*, *Pastinaca sativa*, *Petroselinum crispum*, *Petroselinum sativum*, *Pimpinella anisum*, *Ptychotis verticillata*, and *Ridolfia segetum*. The seeds of these species are mainly employed in infusion to enhance digestion and blood sugar levels [17,18,19,20,21,22,23,24,25,26,27,28,29,30,31,32,33,34,35,37,39,41,45,51,52].

The Leguminosae family encompasses a wide variety of plants renowned for their medicinal properties and nutritional value. Among these, nine noteworthy species are utilized in traditional medicine, particularly in the treatment of various ailments, including diabetes. These species include *Acacia Senegal*, *Acacia tortilis*, *Arachis hypogaea*, *Ceratonia siliqua*, *Ononis natrix*, *Phaseolus aureus*, *Retama raetam*, *Senna alexandrina*, and *Vicia faba*. Their different parts—gums, roots, fruits, leaves, seeds, and aerial parts—are used in various preparations such as decoctions, infusions, powders, and raw forms. These traditional practices not only highlight the versatility of these plants, but also their importance in herbal medicine and nutrition [19,21,25,26,27,28,29,32,33,34,35,37,41,45,51,54]. The Lamiaceae family contains eight species, *Calamintha officinalis*, *Lavandula multifida*, *Lavandula stoechas*, *Mentha pulegium*, *Mentha suaveolens*, *Origanum compactum*, *Origanum vulgare*, and *Thymus algeriensis*. Their leaves are mainly prepared by decoction or infusion [18,19,21,22,23,24,25,26,28,29,30,31,32,33,34,35,37,39,41,51].

The Rosaceae family is a diverse group of flowering plants that includes many well-known fruit-bearing species, some of which have significant medicinal applications. Among these, *Chaenomeles sinensis*, *Crataegus monogyna*, *Malus communis*, *Prunus armeniaca*, *Prunus dulcis*, and *Prunus cerasus* stand out for their health benefits, various parts of which are used in traditional preparations. Their fruits are often consumed raw or juiced to aid digestion and provide a rich source of vitamins. Each species offers unique health benefits by use of its various parts—roots, fruits, seeds, and leaves—prepared in forms such as juices, raw, wines, decoction, and powder [17,19,21,22,23,25,28,29,31,32,33,34,35,39,41,45,46,48,51]. The Poaceae and Solanaceae families each include five species of interest. The Poaceae family includes Avena *sativa*, *Panicum miliaceum*, *Phalaris canariensis*, *Triticum turgidum*, and Zea mays. The seeds of these species are consumed after decoction or infusion, or as a powder. *Z. mays* kernels are used in various dishes, while *A. sativa* is commonly consumed as a porridge, helping to regulate blood sugar levels [20,21,23,24,25,27,32,33,34,46,51]. From the Solanaceae family, *Capsicum annuum*, *Lycopersicon esculentum*, *Nicotiana tabacum*, *Solanum melongena*, and *Withania frutescens* are little-known antidiabetic plants. *S. melongena* is valued for its low carbohydrate content and is used in various culinary preparations. The fruits and leaves of species belonging to this family are consumed raw, or after decoction or infusion [19,21,23,24,33,34].

Among the Brassicaceae, Cistaceae, and Rutaceae families, four species are noteworthy. Brassicaceae is represented by *Brassica napus*, *Brassica nigra*, *Eruca vesicaria*, and *Nasturtium officinale*, which are used in five Moroccan regions as antidiabetic agents. Rhizomes of B. napus are consumed as a juice [19,30], whereas flowers of *B. nigra* are commonly used after infusion or as a powder [21]. The aerial parts of *E. vesicaria* are consumed as a juice or powdered [19,30,34,51]. Leaves and/or stems of *N. officinale* are used after maceration by diabetic patients of the Tan-Tan region [19]. The plants of the Cistaceae family include *Cistus creticus*, *Cistus laurifolius*, *Cistus salviifolius*, and *Cistus ladanifer.* The leaves of these species are used by Moroccan patients after decoction or as a powder [21,25,34,46,51]. The Rutaceae family is represented by *Citrus paradisi*, *Ruta graveolens*, *Ruta chalepensis*, and *Ruta montana*. Different parts of these species, such as leaves, stems and aerial parts, are commonly prepared by decoction or infusion, or as a powder [17,18,19,21,22,23,24,34,35]. Aditionnally, fruits of *C. paradisi* are consumed raw or as a juice [21,30,32,34,39].

In the Amaranthaceae, Cucurbitaceae, Cupressaceae, and Fagaceae families, three species are rarely discussed in the pharmacological literature on diabetes, but are widely used by different Moroccan regions. Plants from the Amaranthaceae family are represented by *Anabasis aretioides*, *Beta vulgaris* and *Spinacia oleracea*. The aerial parts and seeds of these species are used after decoction and infusion, respectively [19,21,45,51]. *Bryonia dioica*, *Citrullus vulgaris*, and *Cucumis melo var. flexuosus* are part of the Cucurbitaceae family. Fruits of these species are commonly consumed raw or after decoction, whereas their leaves are prepared by infusion or macertaion [29,34]. The Cupressaceae family includes *Juniperus phoenicea*, *Juniperus oxycedrus*, and *Tetraclinis articulata*. Their leaves are mainly used after decoction or infusion, or as macerations or powders [18,19,21,22,23,24,26,27,30,32,33,34,35,37,39,41,45,49,51,52]. Furthermore, the Fagaceae family is represented by *Quercus coccifera*, *Quercus suber*, and *Quercus ilex*. Their leaves, fruits and barks are commonly prepared by decoction [21,29,31,32,33,34].

Several families, including Arecaceae, Asparagaceae, Burseraceae, Caryophyllaceae, Chenopodiaceae, Oleaceae, Papaveraceae, Rhamnaceae, and Thymelaeaceae, feature two antidiabetic species little known in the pharmacological context of diabetes. Plants belonging to Arecaceae include *Chamaerops humilis* and *Hyphaene thebaica*. Different parts of *C. humilis* are used raw, powdered, or after decoction or infusion [21,24,30,32,33,34,50], whereas fruits of *H. thebaica* are used as a powder by diabetic patients in the Tan-Tan region [19]. The Asparagaceae family includes *Agave americana* and *Asparagus officinalis*. The leaves of the first species are consumed after decoction by diabetic patients in the Al Haouz-Rhamna region [21], whereas stems of *A. officinalis* are used by patients from Central Plateau regions after cooling in a steamer, or in water [49]. The Burseraceae family includes *Boswellia sacra* and *Commiphora myrrha* species that are known for their resins and fruits, used mostly after decoction or infusion and via ingestion [29,32,34]. Plants of the Caryophyllaceae family include *Paronychia argentea* and *Corrigiola telephiifolia*. The leafy stem of the first species is prepared by infusion, whereas the second’s roots are used as a powder [21,24,30,33,46,49]. *Atriplex halimus* and *Chenopodium ambrosioides* are two antidiabetic species belonging to the Chenopodiaceae family, cited as little-known in the pharmacological context of diabetes. The leaves of these species are commonly used as macerations [19,27,29,30,31,35,37,38,41,42,52]. Moreover, the leaves of the species *Fraxinus angustifolia* and *Olea oleaster*, belonging to the Oleaceae family, are commonly prepared by infusion [24,34]. Two antidiabetic species belonging to the Papaveraceae family, *Fumaria officinalis* and *Plantago ovata*, are used after the infusion or decoction of their leaves, seeds or roots [21,34,41,45,51]. Another important antidiabetic family, Rhamnaceae, is represented by *Ziziphus lotus* and *Ziziphus jujube* species. Their leaves, fruits, and roots are commonly used after decoction or infusion or as a powder [17,18,19,21,22,25,27,29,30,31,33,34,35,37,41,46,51]. Leaves of *Thymelaea hirsute* and *Thymelaea tartonraira* (Thymelaeaceae) are used after decoction by diabetic patients from the Errachidia region, whereas in other regions (Al Haouz-Rhamna, High Atlas Central, and Safi-Essaouira regions), patients use them after infusion or as a powder [17,21,34,35].

Seventeen plant families are represented by only one antidiabetic species that is little known in the pharmacological context of diabetes, but traditionally known for its antidiabetic properties. The Anacardiaceae family is represented by *Pistacia atlantica*; different parts of the plant are used after decoction or infusion, or raw [21,23,24,34,39,51]. In the Apocynaceae family, different parts of *Caralluma europaea* are also employed by diabetic patients through different methods [21,26,29,30,31,32,34,44,46,49]. The Buxaceae family includes *Buxus sempervirens*, with leaves consumed after decoction [18]. Ephedraceae are represented by *Ephedra alata*, the leafy stem of which is prepared by decoction or consumed as a powder [19]. In the Euphorbiaceae family, *Euphorbia resinifera* leaves are consumed by dropping latex in a glass of water [18,24,27,33,34,41,46]. The Gentianaceae family includes *Centaurium spicatum*, the stems and flowers of which are used after infusion [34]. In the Geraniaceae family, the leaves of *Pelargonium odoratissimum* are commonly used after decoction by diabetic patients from the Agadir region [53]. The Myrtaceae family is represented by *Eugenia caryophyllata.* Different parts of this species are prepared using various methods, such as decoction, infusion, maceration, or powdering [33,34,41,51]. Different Moroccan regions use *Sesamum indicum* species (Pedaliaceae) to treat diabetes, especially after decoction, infusion, or powdering [17,19,23,25,27,29,32,34,35,37,39,52]. The Plantaginaceae family is also an important family used in different Moroccan regions as an antidiabetic. *Globularia alypum* is little discussed in the literature as anantidiabetic agent; however, different parts of this species are used after decoction or infusion, or as a poultice [18,19,20,21,22,24,30,33,34,35,39,46]. In the Polygonaceae family, *Emex spinosa* leaves and bulbs are used mainly as a powder [19]. Barks of the *Salvadora persica* species (Salvadoraceae) are used after maceration by diabetic patients from the Rabat-Sale-Kenitra region [32]. In the Schisandraceae family, *Illicium verum* fruits are prepared by decoction by patients in the Al Haouz-Rhamna region [21]. These patients also consume the leaves of *Asphodelus tenuifolius* (Xanthorrhoeaceae) after decoction. Moreover, the underground parts of the *Nardostachys jatamansi* species (Valerianaceae) are commonly consumed after infusion by diabetic patients from Guelmim [52]. In the Verbenaceae family, *Verbena officinalis* leaves are consumed in different Moroccan regions after decoction and infusion [28,30,31,37]. The family Zygophyllaceae includes *Zygophyllum gaetulum*, the aerial parts and leaves of which are prepared by decoction and infusion [29,35].

These lesser-known antidiabetic plants demonstrate the richness of traditional herbal medicine. Their unique properties, parts used, and preparation methods reveal their potential use in supporting blood sugar management. As interest in herbal remedies continues to grow, further research is warranted to validate their traditional uses and explore their roles in modern diabetes management.

#### 3.1.3. Antidiabetic Plants Unknown in Pharmacological Literature of Diabetes

This part presents a selection of 120 plants traditionally used by Moroccan diabetic patients over the last two decades, but which remain unrecognized in the pharmacological literature. The Asteraceae family is one of the largest plant families, and several species are traditionally used for managing diabetes by Moroccan patients. Twenty-three plants species are reported in Moroccan folklore, including *Achillea odorata*, *Achillea santolinoides*, *Antennaria dioica*, *Anvillea garcinii* subsp. *Radiata*, *Artemisia abrotanum*, *Artemisia atlantica*, *Artemisia herba alba Assac*, *Artemisia reptans*, *Centaurea maroccana*, *Chamaemelum mixtum*, *Cladanthus arabicus*, *Cladanthus scariosus*, *Cynara cardunculus* subsp. *scolymus*, *Cynara humilis*, *Echinops spinosissimus*, *Inula conyza*, *Inula helenium*, *Launaea arborescens*, *Scolymus hispanicus*, *Seriphidium herba-alba*, *Sonchus tenerrimus*, *Tanacetum vulgare*, and *Taraxacum campylodes*. Different parts of these species are commonly used to treat diabetes after decoction or infusion [19,21,22,23,24,25,32,34,35,41,45,46,47,51,53]. Aromatic herbs from the Lamiaceae family are often used in Moroccan herbal medicine for treating diabetes. This family is represented by *Ballota hirsuta*, *Calamintha nepeta* subsp. *Spruneri*, *Calamintha alpina*, *Clinopodium alpinum*, *Kuntze Clinopodium nepeta* subsp. *glandulosum*, *Lavandula angustifolia*, *Lavandula dentata*, *Lavandula maroccana*, *Origanum elongatum*, *Thymus broussonetii*, *Thymus maroccanus*, and *Thymus munbyanus*. Different parts of these plants are used, especially the leaves, stems, aerial parts and flowers, prepared mainly by decoction or infusion [19,21,22,23,24,25,28,30,32,33,34,35,41,46,51,52]. Leguminous plants are often included in the diet and traditional medicinal practices of Morocco, contributing to blood sugar control. Eleven species have been reported as antidiabetic plants, including *Acacia gummifera*, *Cassia absus*, *Cytisus battandieri*, *Lupinus angustifolius*, *Lupinus luteus*, *Lupinus pilosus*, *Ononis tournefortii*, *Retama monosperma*, *Retama sphaerocarpa*, *Vicia sativa*, and *Urginea maritima*. The parts used are seeds, leaves and roots, prepared by decoction or infusion or consumed as a powder [17,19,21,25,34,35,41,45,46].

Grasses, widely used as food sources, are also used in traditional medicine for their potential to help regulate blood sugar. Poaceae is also considered a rich family, including *Avena sterilis*, *Castellia tuberculosa*, *Lolium perenne*, *Lolium multiflorum*, *Lolium rigidum*, *Panicum turgidum*, *Phalaris paradoxa*, *Polypogon monspeliensis*, and *Triticum durum*. Their seeds are used through various methods, such as decoction, infusion or ingestion, or consumed raw by diabetic patients [19,21,29,32,34,35,39,46,51,52]. Brassicaceae and Euphorbiaceae families are represented by four species each. These families are known for both edible and medicinal plants, several of which are used traditionally by Moroccan diabetics. In the Brassicaceae family, leaves and stems of *Anastatica hierochuntica* and *Ptilotrichum spinosum* species are prepared by decoction, infusion or powdering in different Moroccan regions [19,24,34,45,52], whereas the flowers of *Diplotaxis pitardiana* are consumed as a powder by diabetic patients from Al Haouz-Rhamna and Tan-Tan [19,21]. Aditionnally, diabetic patients from different Moroccan regions use the bulbs and roots of *Raphanus raphanistrum* subsp. *sativus*, prepared by infusion, maceration, or consumed raw [19,21,25,26,27,29,32,34,37,51]. The Euphorbiaceae family is represented by *Euphorbia officinarum* subsp. *echinus*, *Euphorbia officinarum*, *Euphorbia peplis*, and *Mercurialis annua.* The stems and leaves of these plants are commonly used after decoction, or as a powder [19,20,21,25,30,32,34,45,51,52].

Several families, including Apiaceae, Chenopodiaceae, Moraceae, Oleaceae, Rosaceae, and Urticaceae, are represented by three species each. These families contain several species traditionally used by Moroccan diabetic patients. Plants from the poaceae family are represented by *Ammi majus*, *Anethum foeniculum*, and *Eryngium ilicifolium*. These plants are used by diabetic patients from the Central plateau, Taza and Chtouka Ait Baha and Tiznit regions, respectively. The whole plants of these species are used after decoction, infusion, or as a powder [25,33,49]. Aditionnally, *Hammada scoparia*, *Salsola tetragona*, and *Suaeda mollis* are described in the Chenopodiaceae family. The leaves or seeds of the first species are used after decoction, while the second one’s leaves and fruits are consumed as a powder [19,25,43,54]. Morover, the aerial parts of the third species are consumed in meals [43]. The Moraceae family includes *Ficus abelii*, *Ficus dottata*, and *Morus nigra*. Their leaves are prepared by decoction or infusion [35,45]. Plants from the Oleaceae family include *Fraxinus excelsior var. acuminata*, *Olea europaea* subsp. *maroccana*, and *Olea europea* subsp. *europaea var. sylvestris*. Their leaves, fruits, stems and barks are used mainly in decoctions [35,37]. *Fragaria vesca*, *Rubus fruticosus var. vulgaris*, and *Rubus fruticosus var. ulmifolius* are described in the Rosaceae family. The fruits and leaves of these species are are used after infusion, as a powder, or raw [21,32,33,34]. Three species, including *Urtica pilulifera*, *Urtica urens*, and *Urtica membranacea*, have also been reported in the Urticaceae family as antidiabetic species by Moroccan patients from different regions. Their leaves are prepared mainly by decoction [24,34,35,37].

Various families, such as Apocynaceae, Aristolochiaceae, Caryophyllaceae, Cyperaceae, Ephedraceae, and Thymelaeaceae, are represented by two species each. These families contain a range of species with unexplored antidiabetic potential but that are used in Moroccan traditional medicine. Plants of the Apocynaceae family include *Apteranthes europaea* and *Periploca laevigata* subsp. *Angustifolia*. The leaves, fruits and stems of these plants are mostly used after decoction [25,46]. *Aristolochia baetica* and *Aristolochia longa* subsp. *Fontanesii* are two species belong to the Aristolochiaceae family and known for their roots, resins and seeds, used as powders or after decoction [18,19,21,22,30,35,46]. Plants belonging to the Caryophyllaceae family include *Herniaria glabra var. hirsute* and *Silene vivianii*. The parts of these species that are used are aerial parts and stems, respectively prepared by decoction/powdering or consumed raw [19,35]. Plants belonging to the Cyperaceae family include *Bolboschoenus maritimus* and *Cyperus longus*. The seeds and roots are used after decoction and maceration, respectively, by diabetic patiens from the Al Haouz-Rhamna and High Atlas regions [21,34]. In the Ephedraceae family, the leafy stems of *Ephedra altissima* and *Ephedra fragilis* are used after decoction by Moroccan patients from the Beni-Mellal-Khenifra, Chtouka Ait Baha and Tiznit, and Taroudant regions [25,27,46]. Thymelaeaceae plants, such as *Thymelaea virgate* and *Aquilaria malaccensis*, are reported only in four regions (Al Haouz-Rhamna, Rabat, High Atlas Central, and Guelmim) for use as antidiabetic agents. Their leafy stems and barks are mostly used after decoction by diabetic patients [21,34,35,52].

Several other families contribute to traditional diabetes management in Morocco. These families are represented by one species each. People in Sahara (Tan-Tan) use the leaves of *Limonium sinuatum* (Plumbaginaceae) after decoction of the stems, whereas stems of *Cynomorium coccinum* (Cynomoriaceae) and roots of *Rubia tinctorum* (Rubiaceae) are used as a powder to treat diabetes [19]. *Opophytum theurkauffii* (Aizoaceae) and *Searsia albida* (Anacardiaceae) are also used, where the leaves and fruits are consumed as a powder or after decoction [19]. The leaves of *Maerua crassifolia* (Capparaceae) have been used by patients after decoction or as a powder [19].

The Alliaceae family includes *Allium ampeloprasum var. porrum*, the bulbs and stems of which are used raw, or ingested with water [28,32]. Moreover, diabetic patients from the Middle and High Atlas regions use *Asparagus albus* (Asparagaceae) young sprouts and roots after decoction, or raw [24,34]. *Berberis vulgaris* subsp. *Australis* (Berberidaceae) has also been described as used by Moroccan diabetic patients, especially in Al Haouz-Rhamna, High Atlas Central, Taza, Safi and Essaouira. Fruits, barks, and leafy stems of this species are used after decoction [21,33,34,35,51]. Plants from the Buxaceae family are also known as antidiabetic remedies, especially the *Buxus balearica* species. The leaves of these species are prepared by decoction [21,24]. The Cistaceae plants include *Cistus albidus*, and diabetic patients from the Middle Atlas region use the leaves of this plant after decoction [24]. Patients from this region also use *Juniperus thurifera* (Cupressaceae) leaves after decoction as an antidiabetic agent. Moreover, the bulbs of *Androcymbium gramineum* (Colchicaceae) are prepared by infusion by diabetic patients from Al Haouz-Rhamna [21].

Additionally, *Dracaena draco* subsp. *ajgal* is the only species of the Dracaenaceae family used in the treatment of diabetes by Moroccan patients from Chtouka Ait Baha and Tiznit. The stems and leaves are prepared by decoction to treat diabetes [25]. The family of Equisetaceae is represented by *Equisetum ramosissimum*, which has been used in the High Atlas Central region as an antidiabetic remedy. The patients use its stems after decoction [34]. Patients from this region also use *Pelargonium roseum* (Geraniaceae) leaves after infusion. The Myrtaceae family is represented by Jasminum fruticans species, where the flowers and leaves are prepared by infusion or macerations [34]. *Juncus maritimus* is the only species of the Juncaceae family that has been reportedly used by diabetic patients from Al Haouz-Rhamna and Tan-Tan in traditional medicine [19,21]. These studies describe how the stems and fruits of this species are prepared by decoction to treat diabetes. In the Papaveraceae family, *Papaver rhoeas* seeds are used as a powder by Moroccan diabetic patients from different regions [25,27,29,37,41,46]. *Globularia repens* (Plantaginaceae) species are used after decoction by patients from the Sidi Slimane region [26].

Recently, *Reseda lanceolata* (Resedaceae) has been reported for the first time to be used as an antidiabetic treatment by patients from the Safi and Essaouira regions. Its seeds and leaves are used as a powder, after infusion or via decoction [35]. *Citrus medica var. limon* belongs to the Rutaceae family. The leaves and fruits of these species are prepared by decoction, infusion, macerations, juicing or raw [21,32,34,35,51]. *Salix alba* (Salicaceae) has been reported to be used as a medicinal plant to treat diabetes. The leaves of this species are prepared by decoction [17,48,51]. Furthermore, *Taxus baccata* (Taxaceae) is a very important species known by people from the Al Haouz-Rhamna and High Atlas Central regions to be used as a traditional antidiabetic plant. People from these regions use the plant’s roots after decoction to treat diabetes [21,34]. The leaves of *Aloysia citriodora* (Verbenaceae) are commonly prepared via decoction or infusion [19,20,22,23,32,34]. Tubers of the *Asphodelus microcarpus* species are used after decoction or raw [19,21,34]. The Zygophyllaceae family, including *Tetraena gaetula*, have been used in different Moroccan regions by diabetic patients. The leaves, roots and seeds of this species are used by diabetic patients as a powder or after infusion or decoction [17,18,19,21,23,24,25].

These species reflect the rich cultural heritage of Moroccan herbal medicine, and may hold untapped potential for diabetes management. However, scientific research is required to confirm their efficacy and safety.

### 3.2. Overview of Diabetes in Morocco

In Morocco, diabetes continues to be a serious public health concern. An estimated 2.3 million individuals in the nation, aged 20 to 79, had diabetes as of 2024 [55]. This translates to an approximate 9.8% prevalence rate, with 40.2% of the population with diabetes being undiagnosed [55]. Regional variations in prevalence are notable, with higher rates observed in urban regions as a result of urbanization and lifestyle modifications.

Most cases of diabetes are type 2, which is closely linked to lifestyle factors and obesity. According to a recent study, 21.7% of Moroccans are obese, while 55.1% of them are overweight [56]. These, along with additional comorbidities including dyslipidemia and hypertension, greatly increase the burden of diabetes. There are about 43,000 instances of type 1 diabetes in children and adolescents (ages 0–19) [57]. The main causes of morbidity and death are still diabetes-related complications, such as retinopathy, neuropathy, nephropathy, and cardiovascular disorders. Diabetes is associated with high fatality rates; the condition is responsible for over 31,434 fatalities every year [58]. Among the 344 plants species used in diabetes management in Morocco during the last two decades, 49 were used for diabetes type 1, 79 plants were used for diabetes type 2, 12 plants were used for gestational diabetes mellitus, and 65 species were used for both types. Moreover, nine plants were used for diabetes type 1, diabetes type 2 and gestational diabetes mellitus, seven plants were used for both diabetes type 2 and gestational diabetes mellitus, and only one species was used for both diabetes type 1 and gestational diabetes mellitus (Table 3).

The Moroccan healthcare system continues to face challenges in managing this growing epidemic. Although there have been efforts to increase diabetes awareness and screening, a significant proportion of the population remains undiagnosed. The government has implemented various national plans to combat diabetes, including improving access to healthcare and promoting lifestyle changes [59,60,61]. However, access to insulin and other medications remains a challenge, particularly in rural areas. Moreover, the economic impact of diabetes is substantial, with a significant portion of healthcare expenditure dedicated to managing chronic non-communicable diseases like diabetes. The move towards universal health coverage aims to alleviate some of these burdens, but more comprehensive strategies are needed to address the underlying risk factors and ensure equitable access to care across the country.

### 3.3. Phytochemical Composition of Antidiabetic Medicinal Plants

Based on ethnobotanical survey carried out during the last two centuries, the medicinal species most widely recommended for use in diabetes management are *T. foenum-graecum* (19 regions), *N. oleander*, *R. officinalis*, *S. officinalis*, *O. europaea*, and *N. sativa* (18 regions), *A. cepa and A. herba-alba Asso* (17 regions), *A. sativum*, *M. vulgare*, *L. usitatissimum*, and *F. carica* (15 regions), *C. sativum*, *F. vulgare*, *A. absinthium*, *L. sativum*, *O. ficus indica*, *C. colocynthis*, and *P. granatum* (14 regions), *O. compactum*, *A. iva*, and *P. dulcis* (13 regions), *A. visnaga*, *C. sativus*, *T. articulata*, *G. max*, *M. communis*, *S. indicum*, *Z. lotus*, and *A. spinosa* (12 regions), *C. carvi*, *P. anisum*, *C. spinosa*, *C. siliqua*, *E. globulus*, and *G. alypum* (11 regions), *P. crispum*, *L. stoechas*, *M. pulegium*, *C. sinensis*, and *Z. officinale* (10 regions), and *C. europaea*, *C. cardunculus*, *B. oleracea*, *R. raphanistrum* subsp. *sativus*, *C. ambrosoides*, *L. albu*, *P. harmala*, *C. aurantium*, and *U. dioica* (9 regions). These findings corroborate with those reported in a previous review [62,63], which highlighted that *T. foenum-graecum* was the most useful plants species used in diabetes management in different Moroccan regions. This species is also most commonly recommended for use in other countries, such as southern Italy, India, Bangladesh and China [64,65,66,67].

**Figure 7:**
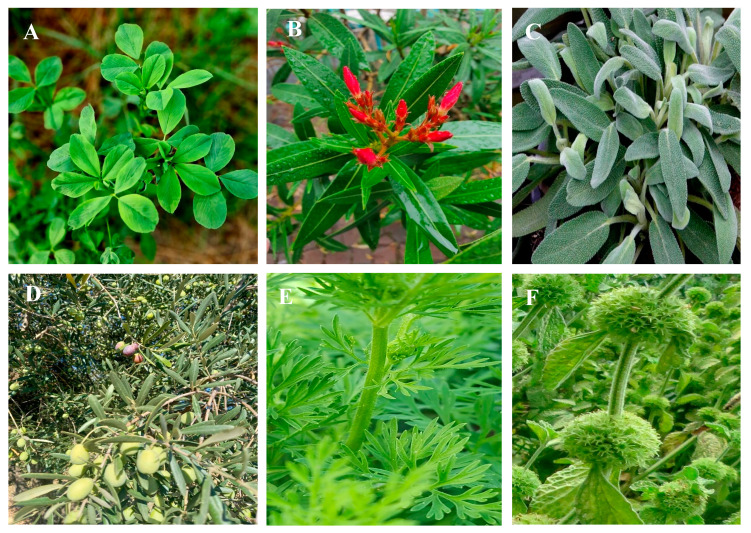
Most useful medicinal plants for diabetes management. (**A**) *T. foenum-graecum*, (**B**) *N. oleander*, (**C**) *S. officinalis*, (**D**) *O. europeae*, (**E**) *N. sativa*, and (**F**) *M. vulgare*.

Several studies have been conducted to find natural alternatives for the treatment of type 2 diabetes. The most effective potential medications are the secondary metabolites found in medicinal plants, such as terpenoids, flavonoids, phenolic acids, and alkaloids. In this section, the results of phytochemical, *in vivo* and *in vitro* studies are reported, but only for the most useful medicinal plants (first ten species) (Figure 7).

#### 3.3.1. *Trigonella foenum-graecum*

This is an age-old adaptable legume, with a long history spanning the Eastern Mediterranean and the Indian subcontinent. Originally grown as a forage crop, this aromatic herb has become a mainstay in many different cuisines around the world, valued for its usage in stews, curries, and syrups [68]. Fenugreek is known for its medicinal properties and has been used in traditional therapeutic techniques for ages, in addition to its culinary uses.

The total carbohydrates in dried fenugreek seeds range from 52% to 58% on average. This includes 24.6–47.6% total dietary fiber, 4.2% accessible carbohydrates, 3.7% starch, 23% crude protein, 8.8% moisture, 6.4% total lipids, and 3.4% ash [69,70]. On the other hand, fresh fenugreek leaves contain approximately 86% moisture, 6% carbohydrates, 4.4% proteins, 1.5% ash, 1.1% fiber, and 0.9% fat [71,72]. Fenugreek seeds have a high nutritional value, according to Bakhtiar et al. [68]. They contain 3.94% ash, 7.94% fat, 10.3% crude fiber, 35.41% protein, and 50.5% carbohydrates. According to Alu’datt et al. [73], fenugreek seed lipids are high in unsaturated fatty acids and antioxidants, such as tocopherols and phytosterols [74]. Their lipid content ranges from 4.5 to 15 g/100 g of seeds. Various phenolic chemicals have been identified in fenugreek leaves, seeds, stems, and flowers, such as total flavonoids (TF), phenolic acids, coumarins, stilbenoids, and tyrosol [75,76]. The total phenolic content (TP) varies between 6.5 and 80 mg GAE/g in the seeds; untreated seeds have lower TP and TF than leaves that have been air-dried [77,78]. The main constituents of fenugreek essential oil (EO) that contribute to its scent and medicinal qualities are neryl acetate, camphor, β-pinene, and α-selinene, among others [79,80].

#### 3.3.2. *Nerium oleander*

*N. oleander* is a popular ornamental plant found in parks, gardens, and roadside plantings. In colder climates, it is occasionally grown inside. Oleander is dangerous despite its attractiveness, since it might be accidentally consumed. A preliminary phytochemical screening showed the presence of alkaloids, carbohydrates, cardiac glycosides, phenolics, flavonoids, tannins, cardenolides, pregnanes, triterpenes, triterpenoids, saponins, and steroids [81,82,83]. The plant accumulates these compounds across its organs, with oleandrin being the most prominent, particularly in the roots (0.34 to 0.64 mg/g dry weight), leaves (0.18 to 0.31 mg/g dry weight), and stem (0.12–0.23 mg/g dry weight) [83]. These concentrations vary according to environmental and genetic factors. The leaves also contain other major products such as cardenolides, neriin, odoroside and gentiobiosyl. Approximately 1.5% of the cardenolides in the leaves is 0.1% oleandrin, or 3-o-α-Loleadrosyl-16-acetylgitoxigenin [84]. Glucosides such as oleandrine, adigoside, and odorosides are found in the seeds, while the bark contains glucosides like rosaginoside, corteneroside, and nerioside [85]. Additionally, a variety of other pharmacologically active compounds have been identified in the plant, including rutin, oleandomycin, folinerin and rosagenin [84].

The flowers contain 1.76% total oil, with 34 compounds identified. The major components include 22.56% neriine, 11.25% digitoxigénine, 8.11% amorphane, 6.58% 1.8-cineole, 5.54% α-pinene, 5.12% calarene, 5.01% limonene, 4.84% β-phellandrene, 3.98% terpinene-4-ol, 3.22% sabinene, 2.94% isoledene, 2.56% 3-carene, 2.29% humulene, 2.01% β-pinene and 1.67% cymen-8-ol [86]. Kaempferol, chlorogenic acid, and kaempferol 3-O-β-glucopyranoside were isolated from the ethyl acetate sub-extracts of flower ethanolic extract [87]. A polysaccharide fraction was isolated from the hot water extract of flowers using ethanol precipitation, cetyltrimethyl ammonium bromide complexing, anion exchange chromatography, and gel permeation chromatography [88].

Few studies have focused on the phenolic fraction. It has been revealed that a high quantity of polyphenols is present in the leaves, with cinnamic acid being the major component. Other components include catechin, epicatechin, and chlorogenic acid. The TP content in flowers was found to be 136.54 mg GAE/g of EO. The TP contents of methanol, water, methanol:water and acetone extracts of the leaves were 4.25, 4.54, 2.08 and 4.21, respectively, and in the flowers, they were 7.15, 7.52, 6.24 and 7.13 μg GAE per 100 μg extract, respectively [89].

#### 3.3.3. *Rosmarinus officinalis*

Growing widely, rosemary is a native of the Mediterranean. Both fresh and extracted leaves are used to flavor and preserve food [90]. Rosemary is characterized by its distinctive camphor scent. Its EO is primarily composed of 1,8-cineole (15–55%), αα-pinene (9.0–26%), camphor (5.0–21%), camphene (2.5–12%), beta-pinene (2.0–9.0%), borneol (1.5–5.0%), and limonene (1.5–5.0%), with the composition varying based on bioclimatic conditions and growth period [91]. The key phytochemicals in *R. officinalis* include rosmarin, caffeic acid, ursolic acid, carnosic acid, camphor, and carnosobetulinic acid [92]. Carnosic acid, which oxidizes into carnosol, is recognized for its photolabile, physicochemical, and thermal properties [93].

Significant rosemary chemotypes are dominated by αα-pinene, cineole, or camphor. The terpenes, including carnosol, ursolic acid, oleanolic acid, and epirosmanol, contribute to rosemary’s therapeutic potential [94]. In the EO, minor components like humulene, cedrene, and caryophyllene coexist with oxygenated compounds like caryophyllene oxide [95]. These terpenes are classified into mono-, di-, tri-, and sesquiterpenes, which are crucial for many bio-natural compounds.

The flavonoids and polyphenols in rosemary, such as luteolin, diosmin, apigenin, genkwanin, chlorogenic acid, caffeic acid, and rosmarinic acid, contribute to its antioxidant properties [96]. The rosmarinic acid, carnosol, and carnosic acid in rosemary extracts are significant antioxidants [97,98]. The extract predominantly contains carnosic acid, carnosol, ursolic acid, and rosmanol, though production levels vary [99]. The triterpenes in rosemary, such as botulin, betulinic acid, 23-hydroxybetulinic acid, ursolic acid, oleanolic acid, 3-epi-α-amyrin, and micromeric acids, are noted for their anti-inflammatory and tumor-inhibitory functions [100]. Key compounds extracted from rosemary also include diosmin, cirsimaritin, and genkwanin [101,102,103]. Rosemary’s diverse bioactive compounds underscore its value in therapeutic and medicinal applications.

#### 3.3.4. *Salvia officinalis*

The EO of *S. officinalis* is a complex mixture of active compounds, primarily consisting of monoterpenes such as α- and β-thujone, camphor, 1,8-cineole, and borneol, along with sesquiterpenes like α-humulene and β-caryophyllene [104,105]. Among these, α- and β-thujone are typically the predominant constituents, although there is considerable chemical variability in the EOs of this plant due to factors such as genetic background, locality, environmental conditions, and the plant’s physiological stage at harvest [106,107]. Research has focused extensively on the chemical composition of its EO across different regions. For instance, a study on 25 indigenous populations in Croatia identified the EO content (1.93–3.7%), with α- β thujone and camphor being the most abundant compounds. This study also revealed three main chemotypes, dominated by α- and β-thujone and camphor/β-pinene/borneol/bornyl acetate [108]. Similarly, an analysis of 12 indigenous populations from Montenegro identified 40 oil constituents as the major components, including α-thujone (16.98–40.35%), camphor (12.75–35.37%), and 1,8-cineole (6.40–12.06%) [109].

In addition to EOs, sage hydrosols and extracts have been extensively studied for their phenolic contents. In the hydrosol headspace, oxygenated monoterpenes such as 1,8-cineole (42.9%), α-thujone (24.3%), β-thujone (14.7%), and camphor (8.9%) predominate, along with monoterpene and sesquiterpene hydrocarbons like β-pinene and β-caryophyllene [110]. The aqueous extracts of *S. officinalis* are particularly rich in flavone glycosides, accounting for about 40% of the total phenolic compounds, with luteolin-O-glucuronide, apigenin-O-glucuronide, and scutellarein-O-glucuronide being the most prevalent [111].

Despite variations in compound concentrations across different studies, rosmarinic acid consistently emerges as a major phenolic in *S. officinalis* extracts. For example, superior levels of rosmarinic acid were found in one cultivar, with 52.7 μg/mg extract, compared to 28.3 μg/mg extract in another [112,113]. Additionally, Silva et al. [113] identified up to 24 phenolic compounds in sage extracts, with cis-rosmarinic acid and luteolin-7-O-glucuronide being the most abundant. These phenolics, along with salvianolic acid and lithospermic acid, were consistently found across various extracts, highlighting the significant role of rosmarinic acid and luteolin derivatives in *S. officinalis*. Further research into sage’s polyphenolic profile identified 18 compounds, primarily hydroxycinnamic acid, rosmarinic acid, and luteolin derivatives. These findings align with those of earlier studies that reported rosmarinic acid and luteolin-7-O-glucuronide as the compounds of highest concentration in sage extracts, underscoring their importance in the plant’s phytochemical profile [114,115].

#### 3.3.5. *Olea europaea*

Olive trees are primarily grown in Mediterranean regions, and the plant is renowned for its fruit, which holds significant economic, nutritional, and medicinal value [116,117]. The phytochemical analysis of *O. europaea* leaves has revealed the presence of a wide variety of compounds, including glycosides, alkaloids, phenolics, flavonoids, coumarins, anthocyanins, tannins, carbohydrates, amino acids, proteins, resins, and fats [118,119]. The leaves contain 49.8% moisture, 1.1% lipids, 7.6% protein, 37.1% carbohydrates, and 4.5% minerals [120,121]. The TP content of the leaves is 125.92 μg GAE/mg of dry extract, with TF at 18 μg CE/mg of dry extract [119]. Five subgroups of phenolics have been identified: flavones, flavonols, flavan-3-ols, oleuropeosides, and substituted phenols, with hydroxytyrosol and oleuropein being the predominant compounds [122].

The EO obtained via hydrodistillation contains several key components, including α-pinene (52.7%), β-pinene (2.46%), and other volatiles such as (E)-2-hexenol (1.26%) and (z)-3-hexanol (1.51%) [123]. Olive fruit consists of 50% moisture, 24.9% carbohydrates, 22% lipids, 1.6% protein, and 1.5% minerals [120,121]. Olive oil is enriched with polyunsaturated fatty acids, carotenoids, and tocopherols, which are essential for protecting against oxidative stress [124]. Additionally, olive oil contains volatile compounds such as isoprene, (E)-Hex-2-enal and α-copaene, and phenolic compounds including hydroxytyrosol, p-coumaric acid, quercetin, and luteolin [125]. Various studies have analyzed the TP contents of olive leaf extracts obtained using different solvents. For instance, the TP content derived using boiling water was found to be 13.39–16.51 mg caffeic acid/g dry matter, with oleuropein concentrations of 13,225–18,694 mg/kg dry matter [126]. The major phenolic compounds identified in 80% aqueous ethanolic olive leaf extracts include 919 mg/kg dry matter of hydroxytyrosol, 312 mg/kg tyrosol, 75 mg/kg caffeic acid, 524 mg/kg ferulic acid, 2406 mg/kg verbascoside, 4221 mg/kg rutin, 6003 mg/kg luteolin-7-O-glucoside, 22,708 mg/kg oleuropein, 6471 mg/kg luteolin-4-O-glucoside and 4537 mg/kg apigenin-7-O-glucoside [126]. The concentrations varied with different extraction methods, highlighting the impact of solvent choice on the yield of bioactive compounds.

The phytochemical diversity of *O. europaea* extends beyond the leaves. The stems and branches are rich in secondary metabolites, including triterpenoids like maslinic acid and erythrodiol, and phenolic substances like taxifolin, comselogoside, and oleuropein [127]. The fruit is notable for its valuable phenolic composition, characterized by flavonoids, secoiridoids, coumarins, phenolic acids, and triterpenoids [128,129,130]. Biophenol secoiridoids, including oleuropein, dimethyl-oleuropein, and ligstroside, along with their hydrolysis derivatives such as oleacein, oleocanthal, and hydroxytyrosol, have been isolated from olive leaves [131,132]. The leaves also contain triterpenes (e.g., maslinic acid, oleanolic acid), coumarins (e.g., scopoletin, aesculetin), alkaloids (e.g., cinchonidine, cinchonine), and chalcones (e.g., olivine-4′-O-diglucoside, olivine) [133]. The olive tree’s bioactive molecules exhibit a wide range of biological activities, including antidiabetic, antibacterial, antifungal, antioxidant, anti-inflammatory, and anticancer effects [134,135,136,137,138]. These activities are largely attributed to the high concentrations of phenolic compounds and triterpenoids found in various parts of the plant.

#### 3.3.6. *Nigella sativa*

*N. sativa*, commonly known as “black seeds”, is widely distributed across North Africa, the Middle East, Europe, and Asia [139]. It has been traditionally used for culinary and medicinal purposes for millennia, particularly in Arab countries, the Indian subcontinent, and Europe [140]. The chemical composition of *N. sativa* is well documented. Subsequent studies have identified that the medicinal value of *N. sativa* is primarily attributed to thymoquinone (TQ) [141]. Other significant components of *N. sativa* include carvacrol, p-cymene, thymohydroquinone (THQ), dihydrothymoquinone (DHTQ), thymol, α-thujene, α, β- pinene, t-anethole, and γ-terpinene [141]. The EO of *N. sativa* contains molecules such as monoterpenoid alcohols, monoterpenes, diterpenes, sesquiterpenes, and ketones, with TQ being a predominant compound [142,143]. *N. sativa* seeds also contain a variety of phenolic compounds, including ferulic acid, gallic acid, vanillic acid, chlorogenic acid, quercetin, p-coumaric acid, catechin, rutin, nigelflavonoside B, apigenin, and flavone [144,145]. Various alkaloids, such as nigellicine (composed of an indazole nucleus) [146], nigellimine (an isoquinoline molecule) [147], and nigellidine (another indazole compound) [148], have been isolated. Saponins, secondary metabolites in *N. sativa*, exhibit a notable affinity for cell membranes due to their amphiphilic nature [149]. In different studies, several saponins have been isolated and identified in the aerial parts of the plant [145], including Kaempferol 3-O-rutinoside, nigelloside, and Flaccidoside.

In various studies, *N. sativa* seeds were found to contain 28.5% fat, 26.7% protein, 24.9% carbohydrates, 8.4% crude fiber, and 4.8% total ash [150,151]. They are also rich in unsaturated fatty acids, primarily linoleic acid (50–60%), oleic acid (20%), dihomolinoleic acid (10%), and eicodadienoic acid (3%). Saturated fatty acids like palmitic and stearic acids make up about 30% or less of the seed’s composition [152,153,154]. NS seeds have also been reported to contain compounds such as avenasterol-5-ene, nigellone, avenasterol-7-ene, 24-methylenecycloartanol, cholesterol, campesterol, citrostadienol, gramisterol, cycloeucalenol, lophenol, stigmastanol, obtusifoliol, stigmasterol-7-ene, butyrospermol, β-amyrin, cycloartenol, and others [155,156]. These compounds contribute to the plant’s rich phytochemical composition, which includes more than 50% terpenoids and terpenes among the identified molecules [157]. *N. sativa* seed oil contains sterols, with β-sitosterol as the major component (48.35–51.92%), followed by 5-avenasterol, campesterol, and stigmasterol [158,159]. *N. sativa*’s extensive phytochemical profile includes a variety of polyphenols, such as kaempferol and quercetin, which contribute to its antioxidant properties. For example, *N. sativa* seeds contain 105.55 g of dry weight polyphenols, with kaempferol and quercetin being the most abundant [160,161].

#### 3.3.7. *Allium cepa*

*A. cepa*, commonly known as onion, is widely used as a vegetable, spice, and in traditional medicine [162]. The bulbs of onion are rich in secondary metabolites, including flavonoids, polyphenols, and steroids/triterpenoids. Notably, fifteen polyphenol compounds have been identified in bulbs, including quercetin derivatives like quercetin 3-glucoside, quercetin 4′-glucoside, and isorhamnetin derivatives [163,164,165,166]. Research has highlighted that onion extracts contain various bioactive compounds. For instance, hot 80% ethanol extraction has been reported to yield carbohydrates such as fructo-oligosaccharides [167]. Moreover, fresh leaf hydrodistillates contain allicin and various disulfides [168], while the 80% methanol extract of dry roots revealed the presence of steroid saponins such as alliospiroside A [169].

Onion skins, which are often discarded as waste, are particularly rich in carbohydrates (88.56%), and also contain protein (0.88%), ash (0.39%), and crude fiber (0.15%) [170]. The skins are a valuable source of phenolic compounds, including quercetin and its derivatives, along with flavonoids, flavanols, anthocyanins, vanillic acid, and ferulic acid. High-performance liquid chromatography has detected numerous polyphenolics in red onion skins, such as catechin, chlorogenic acid, and kaempferol, alongside anthocyanins like cyanidin 3-laminaribioside and cyanidin 3-(6″-malonylglucoside) [171].

Phenolic compounds, derived from cinnamic or benzoic acid, are responsible for the color, flavor, bitterness, and odor of plants. The concentration of these compounds varies between onion varieties, with red skins typically having the highest phenolic content (23.67 free, 12.50 esterified, and 25.45 mg GAE/g bound phenolics), followed by yellow skins (22.71 free, 10.75 esterified, and 17.96 mg GAE/g bound phenolics) [172]. Flavonoids, a significant subgroup of phenolics, are abundant in onions. These include flavonols such as quercetin and kaempferol, and anthocyanins, which contribute to the red or purple color of certain onion varieties. Quercetin derivatives, like quercetin 4′-O-glucoside and quercetin 3,4′-O-diglucoside, represent about 90% of the total flavonoid content in various Allium species, with red onions containing higher amounts than white ones. The flavonoid content in red onion skins ranges from 1.276 to 169 mg/g, compared to 0.08 mg/g in white onion skins [173,174,175]. Phenolic acids like benzoic and cinnamic acid derivatives, along with coumarins and lignans, have also been identified in onions. For example, six coumarins, including scopoletin and esculin, were reported in yellow onion bulbs, and lignans like syringaresinol have been found in onion skins [176,177].

Onion skins also contain organosulfur compounds and phenolic acids. For instance, the total organosulfur compound content in onions is 19%, with onion waste ranging from 15 to 35% [178]. Organosulfur compounds such as trans-(+)-S-1-propenyl-L-cysteine sulphoxide, and other sulfur-containing amino acids contribute to the onion’s characteristic odor and lachrymatory effect [179,180].

#### 3.3.8. *Artemisia herba-alba Asso*

*A. herba-alba*, locally known as “Shih”, is a greenish-silver perennial herb [181]. Renowned for its medicinal properties, this plant has been widely used in traditional medicine across various cultures since ancient times [181,182,183]. EO extraction revealed the presence of fifty-four compounds, representing 94.1% of the total composition [184]. The EO is primarily constituted by 80.3% oxygenated monoterpenes, followed by 10.8% monoterpene hydrocarbons, and 0.2% oxygenated sesquiterpenes. The major compounds include 48.0% α-thujone, 13.4% β-thujone, and 13.1% camphor, with minor components such as 3.6% camphene, 1.4% γ-terpinene, 1.3% borneol, and 1.0% p-cymene [184]. In total, 27 and 10 compounds were identified, representing 96.19% of *A. herba-alba* EO. The major constituents were terpinen-4-ol (37.25%) and ocimene (9.37%) [185]. Amor et al. [186] also reported that oxygenated monoterpenes predominated in *A. herba-alba* EO extracted by hydrodistillation from the Azzemour region, Southwest Morocco, with cis- and trans-thujone, vanillyl alcohol, and nor-davanone as principal constituents. Meanwhile, EO from the Er-rachidia province in south central Morocco was characterized by chrysanthenone and camphor as the main constituents [187]. In contrast, Benabdallah et al. [188] found different dominant compounds in Algerian *A. herba-alba*, including β-copaene (16.22%), limonene (14.56%), and eucalyptol (14.49%).

*A. herba-alba* extract revealed the presence of flavonoids, terpenoids, phenols, tannins, and reducing compounds, with no detection of alkaloids, free quinines, glycosides, or saponins [189]. The RP-HPLC analysis of the aqueous extract indicated the presence of compounds belonging to flavonoids (catechin, apigenin, luteolin) and phenolic acids, with a notable concentration of caffeic acid. Apigenin was also detected in *A. herba-alba* samples from Egypt and Tunisia [190]. The contents of phenolic compounds, flavonoids, and tannins varied between extracts, with the aqueous extract showing the highest concentrations [189]. The TP (263.93 mg GAE/g E), TF (40.94 mg QE/g E), and total tannins (35.99 mg GAE/g E) were significantly higher in the 80% aqueous ethanolic extract than in the methanolic and distilled water extracts. The ethyl acetate extract contained the lowest values of these bioactive compounds [191]. The quantitative and qualitative differences in polyphenol content are influenced by plant origin, solvent nature, and extraction methods [192,193]. Additionally, environmental stress, such as water deficit, can induce phenolic compound synthesis [194].

#### 3.3.9. *Allium sativum*

Garlic is one of the oldest horticultural crops and has been used since ancient times for both culinary and medicinal purposes [195]. Phytochemical analysis revealed that garlic bulbs are rich in sulfur-containing compounds [196], which constitute up to 82% of the total sulfur content [197]. Key compounds include thiosulfinates (e.g., allicin), sulfides (diallyl disulfide, diallyl trisulfide), vinyldithiins (2-vinyl-(4H)-1,3-dithiin, 3-vinyl-(4H)-1,2-dithiin), and ajoenes (E-ajoene, Z-ajoene) [197,198]. Allicin, derived from alliin via the allinase enzyme upon cutting or crushing garlic, is one of the main bioactive molecules, along with S-methyl cysteine-sulfoxide and S-propyl-cysteine-sulfoxide, which are responsible for garlic’s characteristic odor [198]. These sulfur compounds can further transform into other molecules such as allyl methane thiosulfinates and methyl methanethiosulfonate, depending on water content, temperature, and enzymatic activity [198].

Garlic formulations also contain other organosulfur compounds like N-acetylcysteine, S-allyl-cysteine, and S-ally-mercapto cysteine, all of which are derived from alliin [199,200]. In quantitative studies, garlic extracts have been reported to contain 65 µg/mL chlorogenic acid, 44 µg/mL p-coumaric acid, and 25 µg/mL 4-hydroxybenzoic acid [201]. The TP in garlic varies between 11.05 and 20.63 mg GAL/g DM, while TF ranges from 0.94 to 2.12 mg QE/g DM [202]. The allicin content in garlic ranges between 3.69 and 7.12 mg/g DM, and alliin ranges between 2.5 and 5.38 mg/g DM [202]. Garlic is also reported to contain a variety of other bioactive compounds, including saponins, steroids, flavonoids, phenols, tannins, and cardiac glycosides [203].

#### 3.3.10. *Marrubium vulgare*

*M. vulgare*, native to the region between the Mediterranean Sea and Central Asia, is now widespread across all continents [204]. The plant produces trace amounts of EO, primarily composed of monoterpenes such as camphene, fenchene, p-cymol, limonene, sabinene, α-pinene, and α-terpinolene [205]. Non-volatile monoterpene derivatives like marrubic acid and sacranoside A, along with sesquiterpene lactone vulgarin, β-sitosterol, lupeol, and triterpenoids such as oleanolic acid, have been identified in *M. vulgare* extracts [206,207,208,209]. Diterpenes of the labdane type, including 0.12–1% marrubiin, 0.13% pre-marrubiin, and other related compounds, are the principal bitter components [210,211,212].

In terms of phenolic compounds, *M. vulgare* is rich in phenolic acids, cinnamic acids, and flavonoids. The total cinnamic acid derivatives are estimated at 14.09 mg/100 mg of dry material, with condensed tannins at 16.55 mg catechin/100 g [213,214]. Specific compounds include gallic, gentisic, and syringic acids; trans-cinnamic, ferulic, and p-coumaric acids; and hydroxycinnamic acid derivatives such as acteoside [215,216,217]. Flavonoid fractions contain apigenin, luteolin, chrysoeriol, and diosmetin, among others [216]. *M. vulgare* also accumulates marrubiin in its leaves and trichomes, with levels influenced by the plant’s developmental stage. The central diterpenoid precursor, geranylgeranyl pyrophosphate, is crucial for the biosynthesis of marrubiin and related metabolites [218,219]. Studies on *M. vulgare* EO reveal significant variation across regions. Major components include germacrene D, β-caryophyllene, and bicyclogermacrene, with some studies also identifying E-caryophyllene and β-bisabolene as key constituents [220,221,222,223,224,225]. Additionally, horehound extracts are rich in polyphenols (55.72 mg gallic acid equivalent/mL), flavonoids (11.01 mg catechin equivalent/mL), phenolic acids (4.33 mg caffeic acid equivalent/mL), and condensed tannins (4.46 mg delphinidin equivalent/mL) [226,227].

**Figure 8:**
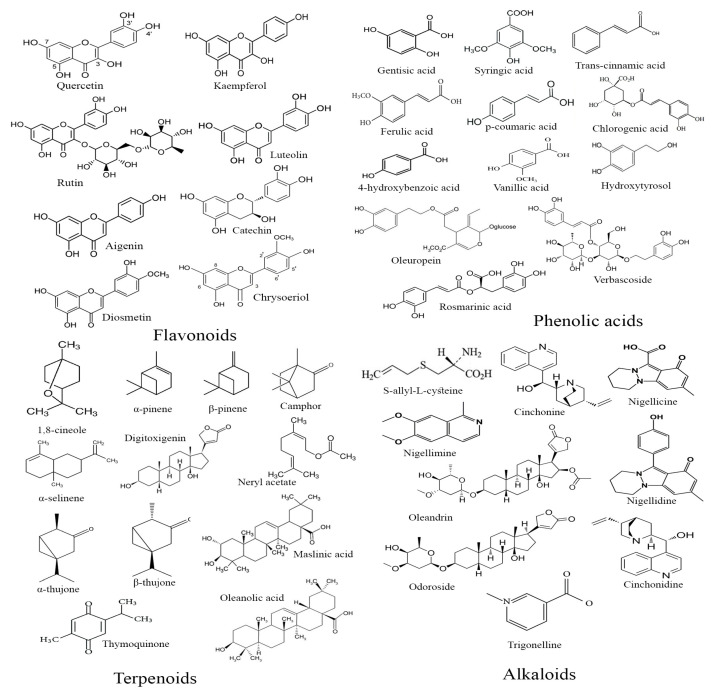
Chemical structures of the known natural compounds useful against diabetes.

Moroccan medicinal plants traditionally used for diabetes management, and studied herein, contain bioactive compounds with proven antidiabetic properties (Table 4, Figure 8). For example, we can list the following:

- **Flavonoids**. *T. foenum-graecum*, *O. europeae*, *N. sativa*, *A. sativum*, and *A. cepa* have been reported to be rich in flavonoids, including quercetin and kaempferol, which are known for their antioxidant and hypoglycemic effects;
- **Phenolic Acids**. *R. officinalis*, *S. officinalis*, *A. sativum*, and *M. vulgare* contain significant amounts of phenolic acids such as rosmarinic acid, which is linked to glucose metabolism regulation and insulin sensitivity;
- **Terpenoids**. Plants like *T. foenum-graecum*, *N. oleander*, *O. europeae*, *N. sativa*, *A. cepa*, *A. herba-alba Asso*, and *M. vulgare* have demonstrated a high content of terpenoids, which contribute to their antidiabetic and anti-inflammatory activities;
- **Alkaloids**. Alkaloids have been identified in *N. oleander*, *O. europeae*, and *N. sativa*, which are known to influence insulin release and glucose absorption pathways.

### 3.4. In Vivo and In Vitro Antidiabetic Effects of Moroccan Medicinal Plants

Diabetes mellitus, a global health challenge characterized by chronic hyperglycemia due to impaired insulin secretion, insulin action, or both, is often managed with synthetic drugs that can cause significant side effects. Consequently, there is growing interest in natural alternatives, including Moroccan medicinal plants, which have been extensively studied for their antidiabetic properties [228,229]. These plants have demonstrated *in vivo* potential to reduce blood glucose levels, enhance insulin secretion, protect pancreatic β-cells, and stimulate glycogen biosynthesis, as evidenced by 133 manuscripts investigating their effects.

Enzymes like α-amylase, α-glucosidase, and β-glucosidase control the degradation of carbohydrates in the intestine, which raises blood glucose levels. The inhibition of these enzymes is a key strategy for managing type 2 diabetes [230,231]. Although synthetic inhibitors like acarbose are effective, they are associated with adverse effects such as digestive disorders and increased liver enzyme levels [232,233,234]. As a result, research has focused on plant-derived alternatives, including Moroccan medicinal plants rich in secondary metabolites like alkaloids, phenolic acids, flavonoids, and terpenoids, which have shown significant *in vitro* antidiabetic effects [230,231]. Notably, the 10 Moroccan medicinal plants most widely used, belonging to six botanical families, have been tested for their *in vivo* antidiabetic activity against these enzymes, with some also showing *in vitro* efficacy (Table 5) [235,236,237,238,239,240,241,242,243,244,245,246,247,248,249,250,251,252,253,254,255,256,257,258,259,260,261,262,263,264,265,266,267,268,269,270,271,272,273,274,275,276,277,278,279,280,281,282,283,284,285,286,287,288,289,290,291,292,293,294,295,296,297,298,299,300,301,302,303,304,305,306,307,308,309,310,311,312,313,314,315,316,317,318,319,320,321,322,323,324,325,326,327,328,329,330,331,332,333,334,335,336,337,338,339,340,341,342,343,344,345,346,347,348,349,350,351,352,353,354,355,356,357,358,359,360,361,362,363].

**Table 4:** Chemical compounds of the most useful antidiabetic Moroccan medicinal plants.

Plant Species	Used Parts	Extract/EO	Groups	Compounds	References
***T. foenum-graecum***	Leaves/seeds/stems/flowers	Aqueous extract	Flavonoids	Quercetin/kaempferol	[75]
	Stems	Aqueous extract	Phenolic acids	Gallic acid/caffeic acid	[75,76]
	Seeds	EO	Terpenoids	Neryl acetate/camphor/β-pinene/α-selinene	[79,80]
	Seeds	4-hydroxyisoleucine	Alkaloids	Trigonelline	[243]
***N. oleander***	Seeds	Aqueous extract	Flavonoids	Rutin/kaempferol	[84,87]
	Flower/Leaves	Ethanolic extract	Phenolic acids	Cinnamic acid/chlorogenic acid	[89]
	Flowers	EO	Terpenoids	Neriine/digitoxigenin	[86]
	Leaves/Seeds	Aqueous extract	Alkaloids	Oleandrin/odoroside	[83,84]
***R. officinalis***	Aerial parts	Aqueous extract	Flavonoids	Luteolin/apigenin/diosmin	[96]
	Aerial parts	Aqueous extract	Phenolic acids	Rosmarinic acid/caffeic acid	[96]
	Aerial parts	EO	Terpenoids	1,8-cineole/α-pinene/camphor/carnosol/ursolic acid	[91,93]
***S. officinalis***	Aerial parts	Aqueous extract	Flavonoids	Luteolin/apigenin	[111]
	Powder	Aqueous extract	Phenolic acids	Rosmarinic acid/salvianolic acid	[113,114,115]
	Leaves	EO	Terpenoids	1,8-cineole/α-β thujone/camphor	[108,109]
***O. europaea***	Fruits	Oil	Flavonoids	Quercetin/luteolin/apigenin	[125]
	Leaves	Oil/Aqueous extract	Phenolic acids	Hydroxytyrosol/oleuropein/verbascoside	[126,312]
	Leaves/stems/branches	Aqueous extract	Terpenoids	Maslinic acid/oleanolic acid	[127,133]
	Leaves	Aqueous extract	Alkaloids	Cinchonidine/cinchonine	[133]
***N. sativa***	Seeds	Aqueous extract	Flavonoids	Quercetin/rutin/apigenin/catechin/nigelflavonoside B.	[144,145]
	Seeds	Aqueous extract	Phenolic acids	Ferulic acid/gallic acid/vanillic acid/chlorogenic acid/p-coumaric acid	[144,145]
	Seeds	EO	Terpenoids	Thymoquinone/THQ/DHTQ/α-thujene/β-pinene/γ-terpinene.	[141]
	Seeds	Ethanolic extract	Alkaloids	Nigellicine/nigellimine/nigellidine	[147,148]
***A. cepa***	Bulbs	Aqueous extract	Flavonoids	Quercetin 3-glucoside/quercetin 4′-glucoside/isorhamnetin	[163,164,165,166]
	Onion skins	Ethanolic extract	Phenolic acids	Chlorogenic acid/vanillic acid/ferulic acid	[171]
	Roots	Methanol extract	Terpenoids	Allicin/disulfides/steroid saponins (alliospiroside A)	[169]
***A. herba-alba***	Aerial parts	Aqueous extract	Flavonoids	Apigenin/catechin/luteolin.	[190]
	Leaves/Aerial parts	Aqueous extract	Phenolic acids	Caffeic acid/tannins	[189,190]
	Leaves	EO	Terpenoids	α- β-thujone/camphor/terpinen-4-ol/ocimene	[184]
***A. sativum***	Bulbs	Aqueous extract	Flavonoids	Quercetin (trace)	[203]
	Bulbs	Aqueous extract	Phenolic acids	Chlorogenic acid/p-coumaric acid/4-hydroxybenzoic acid	[201]
	Bulbs	EO	Terpenoids	Allicin, diallyl disulfide, diallyl trisulfide, ajoene.	[197,198]
	Bulbs	Aqueous extract	Alkaloids	S-allyl cysteine	[198]
***M. vulgare***	Aerial parts	Aqueous extract	Flavonoids	Apigenin/luteolin/chrysoeriol/diosmetin	[216]
	Aerial parts	Aqueous extract	Phenolic acids	Gallic acid/gentisic acid/syringic acid/cinnamic acid/ferulic acid/p-coumaric acid	[215,216,217]
	Flowers/Aerial parts/Leaves	EO	Terpenoids	Marrubic acid/marrubiin/germacrene D/β-caryophyllene/bicyclogermacrene.	[220,221,222,223,224,225]

**Table 5:** *In vitro* and *in vivo* studies of Moroccan medicinal plants used in diabetes management.

Family	Species	Extracts	Parts Used	Administrated Dose	Model/Experimental Methods	Key Results	References
Leguminosae	*Trigonella foenum-graecum*	Methanolic extract	Seeds	2 g/kg	Oral glucose tolerance test Normal albino rats	Reduction in blood glucose	[235]
		Hydroalcoholic extract	Seeds	100 μL of extract for α-amylase/60 μL of extract for α-glucosidase	α-amylase and α-glucosidase inhibition assay	High inhibitory activity of α-amylase and α-glucosidase	[236]
		Aqueous extract	Seeds	300 mg/kg	STZ-induced diabetic rats	IM6E demonstrated strong α-glucosidase activity and moderate α-amylase and invertase inhibition activities under *in vitro* conditions	[237]
		Ethanolic extract	Seeds	1 g/kg	Normal and alloxan-induced diabetic rats	Decreased blood glucose to 12.40% level in alloxan-induced rats No acute toxicity	[238]
		Aqueous extract	Seeds	0.44/0.87/1.74 g/kg for 6 weeks	STZ-induced diabetic rats	Increases body weight and decreases fasting blood glucose	[239]
		Aqueous extract	Seeds	2.5 g/kg	Normal and alloxan induced diabetic rabbits	Reduction in plasma glucose levels in the fenugreek-treated rabbits	[240]
		Ethanolic extract	Seeds	25 g seed mucilage/rat/day	STZ-induced diabetic rats	Amelioration of the diabetic state	[241]
		Aqueous extract	Seeds	100 mg/kg	STZ-induced diabetic rats	Reduced blood glucose levels Urea levels decreased following daily intraperitoneal injection	[242]
		Solution of 4-hydroxyisoleucine	Seeds	50 mg/kg	Single and repeated injection STZ-induced type I diabetic rats	Levels of insulin are reduced by 65%	[243]
		Hydroalcoholic extract	Seeds	400 mg/kg	STZ-induced diabetic rats	Decreased blood glucose levels	[244]
		Powder	Seeds	5 g of dry FSP mixed with 95 g of powdered rat feed) for 21 days	Alloxan induced diabetic rats	FSP treatment increased insulin levels in diabetic rats to nearly 80%	[245]
Apocynaceae	*Nerium oleander*	Aqueous extract	Leaves	Nd	a-amylase inhibition assay	Breakdown of starch to maltose, maltotriose, various oligoglucans is mediated by α-amylase enzyme followed by subsequent α-glucosidase activity to finally yield glucose	[246]
		Powder	Leaves	16 g dry leaves/kg	Normal rats	Inhibitory activity of α-glucosidase Reduced the blood glucose level in maltose- and sucrose-loaded rats at very high dose of 16 g/kg	[247]
		Methanolic extract	Leaves	200 mg/kg	Alloxan induced diabetic rats	Reduced blood glucose level by 73.79% OGTT revealed increase in glucose tolerance by 65.72% No mortality was observed in the experiment	[248]
		Methanolic extract	Flowers	Nd	Rats L6 myogenic cells	Decreasing the blood glucose level and inhibition of α-amylase	[249]
		Plant extract	Nd	250 mg/kg for 4 weeks	STZ-induced diabetic rats	Improvement in insulin and glucose levels	[250]
		Ethanolic extract	Flowers	225 mg/kg	STZ-induced diabetic rats	Decrease glucose level	[251]
		Powder	Shoots	375 μg/0.5 mL of distilled water for 12 weeks	High-fat-diet-fed STZ-induced diabetic rats	Reduced fasting blood glucose	[252]
		Chloroform and ethanolic extract	Leaves	50 mg to 5000 mg/kg	Alloxan-induced diabetic rats	Prevented body weight loss in diabetic rats No sub-acute glucose reduction	[253]
Lamiaceae	*Rosmarinus officinalis*	EO	Leaves	250 µl	α-amylase inhibition assay	Inhibitory activity of α-amylase	[254]
		Aqueous extract	Aerial parts	100 µg/20 µL distilled water	α-glucosidase inhibition assay	High inhibitory activity of α-glucosidase	[255]
		Ethanolic extract	Leaves	100 mg of RAE	α-amylase inhibition assay	Inhibited amylase activity by 85%	[256]
		Diethyl ether and n-butanol extract	Leaves	800 mg/kg	α-glucosidase assay Oral glucose tolerance test Normal and STZ-induced diabetic rats	Inhibitory activity of α-glucosidase Decrease glucose level Inhibited glucose intestinal transport	[257]
		Ethanolic extract	Leaves	20 mg/0.6 water	Normal and STZ-induced diabetic rats	Strong α-glucosidase inhibitory	[258]
		Powder	Leaves	12% for 6 weeks	Normal and STZ-induced diabetic rats	Reduced fasting blood glucose	[259]
		Ethylacetate extract	Nd	300 mg/kg	Normal and alloxan-induced diabetic rats	Reduced fasting blood glucose	[260]
		Aqueous extract	Leaves	200 mg/kg for 21 days	Normal and STZ-induced diabetic rats	Reduced the glucose level	[261]
		Aqueous extract	Leaves	1.11 gm/mL/day	Normal and STZ-induced diabetic rats	Reduced blood glucose level Reduced fasting plasma glucose	[262]
		Aqueous extract	Leaves	200 mg/kg for 21 days	Normal and STZ-induced diabetic rats	Reduced fasting plasma glucose	[263]
		Aqueous extract	Leaves	200 mg/kg for 21 days	Normal and STZ-induced diabetic rats	Reduced fasting plasma glucose	[264]
		Powder	Leaves	5 g/100 g diet	Normal and STZ-induced diabetic rats	Reduced blood glucose level	[265]
		Aqueous extract	Leaves	200 mg/kg for 21 days	Normal and STZ-induced diabetic rats	Increased serum insulin, C-peptide while decreased ALT and aspartate aminotransferase	[266]
		Aqueous extract	Leaves	200 mg/kg/day	STZ-induced diabetic rats	Increased serum insulin level Reduced fasting plasma glucose	[267]
		Aqueous extract	Leaves	200 mg/kg for 21 days	STZ-induced diabetic rats	Reduced blood glucose level Reduced antioxidant status of diabetic rats	[268]
		Rosmarinic acid	Leaves	120–200 mg/kg	STZ-induced type 1 diabetes rats or high-fat-diet (HFD)-induced type 2 diabetes rats	Decreased plasma glucose levels and improved insulin sensitivity	[269]
		Rosmarinic acid	Leaves	577 µg/mL	STZ-induced diabetic rats High-fat-diet-induced diabetic rats	Reduced fasting plasma glucose Increased insulin levels without affecting liver glycogen levels	[270]
		Ethanolic extract	Leaves	200 mg/kg for 7 days	Alloxan-induced diabetic rats	Reduced fasting plasma glucose and increased serum insulin	[271]
		Powder	Leaves	20% of powder for 45 days	Alloxan-induced diabetic rats	Reduced fasting plasma glucose	[272]
		Rosmarinic acid	Leaves	100–200 mg/kg for 8 weeks	Alloxan-induced diabetic rats	Inhibited glomerular hypertrophy, glomerular number loss and glomerulosclerosis	[273]
	*Salvia officinalis*	Aqueous extract	Aerial parts	Nd	α-amylase and α-glucosidase inhibition assay	Inhibitory activity of α-amylase and α-glucosidase	[274]
		EO	Leaves	5% to 75%	α-glucosidase inhibition assay	Inhibitory activity of α-glucosidase	[275]
		Aqueous extract	Aerial parts	50 µL	α-glucosidase inhibition assay	Inhibitory activity of α-glucosidase	[276]
		Ethanolic extract	Leaves	0–200 µg	α-glucosidase inhibition assay	Inhibitory activity of α-glucosidase	[112]
		Water and ethanolic extract	Nd	12%	α-glucosidase inhibition assay	Inhibitory activity of α-glucosidase	[277]
		Ethylacetate extract	Aerial parts	20–300 mg/mL	α-amylase and α-glucosidase inhibition assay	Inhibitory activity of α-amylase and α-glucosidase	[278]
		Methanolic extract	Leaves	250 and 500 mg/kg for 21 days	α-glucosidase inhibition assay Oral glucose tolerance test Normal and alloxan-induced diabetic rats	Inhibitory activity of α-glucosidase Reduced postprandial blood glucose	[279]
		Ethanolic extract	Leaves and flowers	300 mg/kg	Alloxan induced diabetic rats	Reduced blood glucose and cholesterol	[280]
		Ethanolic extract	Leaves	0.2 and 0.4 g/kg for 14 days	Normal and STZ-induced diabetic rats	Reduction in serum glucose and increased plasma insulin in	[281]
		Aqueous and ethanolic extracts	Leaves	100 mg/kg for 14 days	Normal and alloxan-induced diabetes in white rats	Reduced blood glucose	[282]
		Water ethanol extract	Leaves	500 mg/kg	Normal and alloxan-induced diabetic mice	Reduced blood glucose	[283]
		Aqueous extract	Leaves	300 mg/kg for 5 weeks	Normal and alloxan-induced diabetes rats	Reduced blood glucose	[284]
		Aqueous extract	Leaves	400 and 600 mg/kg for 7 days	Alloxan-induced diabetic mice	Reduced fasting blood glucose	[285]
		Methanolic extract	Leaves	100–500 mg/kg	STZ-induced diabetic rats	Decreased serum glucose after 3 h of administration	[286]
	*Marrubium vulgare*	Aqueous extract	Leaves	400 mg/kg	α-amylase inhibition assay Normal rats	Inhibitory activity of pancreatic α-amylase Reduced blood glucose	[287]
		Hydro-alcoholic extract	Leaves	Nd	α-amylase inhibition assay	Inhibitory activity of pancreatic α-amylase	[288]
		Methanolic extract	Aerial parts	500 mg/kg for 28 days	STZ-induced diabetic rats	Increased plasma insulin Reduced blood glucose	[289]
		Methanol, water and butanol extract	Whole plant	1 and 2 mg/mL for 28 days	Cyclosporine A and STZ-induced diabetic rats	Induced autoimmune diabetes mellitus-type1 induced by cyclosporine A and STZ in mice	[290]
		Aqueous extract	Aerial parts	100, 200 and 300 mg/kg	Normal and alloxan-induced diabetes rats	Increased plasma insulin and tissue glycogen	[214]
		Aqueous extract	Leaves	300 mg/kg	Normal and alloxan-induced diabetes rats	Increased plasma insulin Reduced blood glucose	[291]
		Ethanolic extract	Whole plant	100 mg/kg	Normo-glycemic rats	Increased plasma insulin Reduced blood glucose	[292]
Oleaceae	*Olea europaea*	Alcoholic extract	Leaves	0.1, 0.25 and 0.5 g/kg for 14 days	Normal and STZ-induced diabetic rats	Decreased the serum glucose Increased the serum insulin in diabetic rats	[293]
		Nd	Leaves	1 g/kg for 14 days	STZ-induced diabetic rats	Decreased blood glucose level	[294]
		Alcoholic extract	Leaves	1 g/kg	Single and repeated injection STZ-induced diabetic rats	Improved glucose homeostasis through the reduction of starch digestion and absorption	[295]
		Aqueous extract	Leaves	100 and 200 mg/kg	STZ-induced diabetic rats	Decreased serum glucose level	[296]
		Powder	Leaves	6.25%	STZ-induced diabetic rats	Decreased serum glucose level by 38%	[297]
		Ethanolic extract	Leaves	300 and 500 mg/kg/day	STZ-induced diabetic rats	Inhibited high-glucose-induced neural damage	[298]
		Ethanolic extract	Leaves	3 and 5 mg/kg	STZ-induced diabetic rats	Thymoquinone and oleuropein significantly decrease serum glucose levels	[299]
		Aqueous extract	Leaves and fruits	1 g/kg	Normal and STZ-induced diabetic rats	Decreased blood glucose level at 4th week compared to the diabetic control rats	[300]
		Powder	Leaves	17.8 mg/kg	STZ-induced diabetic rats	Reduced blood glucose tolerance curve	[301]
		Aqueous extract	Leaves	200 and 400 mg/kg	Normal and STZ-induced diabetic rats	Decreased serum insulin level	[302]
		Ethanolic extract	Leaves	200 and 400 mg/kg for 10 weeks	HFD STZ-induced diabetic rats	Increased serum insulin level	[303]
		Aqueous extract	Leaves	1% and 3%	STZ-induced diabetic rats	Exerted antihyperglycemic effects via AS160 inhibition	[304]
		Aqueous extract	Leaves	1 mg/mL 200 mg/kg	α-glucosidase inhibition assay Normal and STZ-induced diabetic rats	Strong α-glucosidase inhibitory activity Reduced blood glucose	[305]
		Ethanolic extract	Leaves	100 mg/kg	Normal and HFD rats	Reduced blood glucose and insulin levels	[306]
		Alcoholic extract	Leaves	8 and 16 mg/kg	Alloxan-induced diabetic rats	Decreased serum glucose level	[307]
		Aqueous extract	Leaves	3% and 6%	Alloxan-induced diabetes rats	Decreased blood glucose level	[308]
		Aqueous extract	Leaves	100–600 mg/kg	Normal and alloxan-induced diabetes rats	Decreased blood glucose level Increased plasma insulin level	[309]
		Hydroethanolic extract	Leaves	5–20 mg/kg for 40 days	Normal and alloxan-induced type 1 diabetic rats	Decreased blood glucose level	[310]
		Ethanolic extract	Leaves	600 mg/kg	Alloxan-induced diabetic rabbits	Reduced blood glucose level by 20%	[311]
		Aqueous extract	Leaves	20 mg/kg for 16 weeks	Normal and alloxan-induced diabetes rabbits	Decreased blood glucose level	[312]
		Ethanolic extract	Leaves	3.85 mg/ml	α-glucosidase inhibition assay	Inhibitory activity of α-glucosidase	[313]
		Hydro-alcoholic extract	Oil	500 to 31.25 mg/mL.	α-glucosidase and α-amylase inhibition assay	Inhibitory activity of α-glucosidase Less inhibitory activity of α-amylase	[314]
		Ethyl acetate extract	Stems	10 µL	α-amylase inhibition assay	Inhibitory activity of α-amylase	[315]
		Hydro-alcoholic extract	Leaves	100–600 µM	α-glucosidase and α-amylase inhibition assay	Inhibitory activity of α-glucosidase Less inhibitory activity of α-amylase	[134]
Ranunculaceae	*Nigella Sativa*	Aqueous extract	Seeds	10–50 μL	α-glucosidase inhibition assay	Inhibitory activity of α-glucosidase	[316]
		Ethanolic extract	Seeds	2 g/kg for 4 weeks	Oral glucose tolerance test	Hypoglycemic and hypolipidemic activity	[299]
		Aqueous extract	Seeds	2 g/kg	Oral glucose tolerance test	Improved glucose tolerance in rats	[317]
		Aqueous methanol Oil	Seeds	810 mg/kg for 25 days 2.5 mL/kg for 25 days	Normal and alloxan-induced diabetes rats	Administration of the crude methanolic extract and the oil decreased significantly the blood glucose after 10 days of treatment	[318]
		Methanolic extract/Oil	Seeds	2.5 mL/kg for 24 days	Normal and alloxan-induced diabetes rabbits	Decreased blood glucose level	[319]
		Ethanolic extract	Seeds	20 and 40% of pulverized extract (for 24 days)	Normal and alloxan-induced diabetes rats	Decreased blood glucose level	[320]
		Ethyl acetate fraction of Ethanolic extract	Seeds	200–1000 mg/kg	Alloxan-induced type 2 diabetes rats	Reduced blood glucose level	[321]
		Ethanolic extract	Seeds	100, 200, and 400 mg/kg for 6 weeks	STZ-induced diabetic rats	Decreased serum glucose level	[322]
		Methanolic extract	Seeds	500 mg/kg	STZ-induced types 2 diabetic rats	Reduced postprandial glucose, and improved glucose tolerance in rats	[323]
		Nd	Seeds	0.5–1.5 mL	STZ-induced diabetic rats	Reduced serum glucose level	[324]
		Ethanolic extract	Seeds	300 and 600 mg/kg for 7 days	HFD STZ-induced diabetic rats	Reduced blood glucose level	[325]
		Ethanolic extract	Seeds	100 mg/kg for 28 days	STZ-induced diabetic rats	Decreased blood glucose level	[326]
		Oil	Seeds	400 mg/kg for 4 weeks	STZ-induced diabetic hamsters	Decreased blood glucose level	[327]
		Oil	Seeds	2 mg/kg for 30 days	STZ-induced diabetic rats	Reduced fasting blood glucose and increased insulin levels	[328]
		Petroleum ether extract	Seeds	2 g/kg for 4 weeks	STZ-induced diabetic rats	The petroleum ether extract exerted an insulin-sensitizing action	[329]
		Ethanolic extract	Seeds Polys	35–140 mg/kg for 4 weeks	HFD STZ-induced types 2 diabetic rats	Reduced fasting plasma glucose and increased serum insulin	[330]
Alliaceae	*Allium cepa*	Ethyl alcohol extract Quercetin	Skin	1–3 mg/mL	α-amylase and α-glucosidase inhibition assay	Inhibitory activity of α-amylase and α-glucosidase	[331]
		Methanolic extract	Skin	Nd	α-glucosidase inhibition assay	Inhibitory activity of α-glucosidase	[332]
		Ethanolic extract	Skin	30 mg/mL 0.1–0.5 mg/mL	α-amylase inhibition assay α-glucosidase inhibition assay	Inhibitory activity of α-amylase α-glucosidase assay	[333]
		Aqueous extracts	Skin	0.01–10 mg/mL	α-amylase inhibition assay	Inhibitory activity of α-amylase	[334]
		Hydroethanolic extract	Skin	10 µg/mL	α-glucosidase inhibition assay	Inhibitory activity of α-glucosidase	[335]
		Hydromethanolic extract	Skin	Nd	α-glucosidase inhibition assay	Inhibitory activity of α-glucosidase	[336]
		EO	Bulbs	100 mg/kg for 21 days	STZ-induced diabetic rats	Deceased blood glucose and increase in serum insulin	[337]
		Ethanolic extract	Bulbs	150 and 300 mg/kg	Normal and STZ-induced diabetic rats	Decreased fasting blood glucose Increased serum insulin levels	[338]
		Ethanolic extract Quercetin	Bulbs	0.5 or 1% for 8 weeks 0.1% for 8 weeks	Oral glucose tolerance test Normal and HFD STZ-induced diabetic rats	Improves insulin sensitivity by upregulating expressions of insulin receptor and glucose transporter	[339]
		Powder	Bulbs	0.5 and 2% for 4 weeks	Normal and HFD STZ-induced diabetic rats	Serum insulin concentrations and insulin resistance were dose-dependently increased in the onion-fed groups	[340]
		Aqueous extract	Whole plant	200–300 mg/kg for 6 weeks	Alloxan-induced diabetic rats	Reduced fasting blood glucose level by 75.4% at 300 mg/kg	[341]
		Aqueous extract	Bulbs	1 mL for 4 weeks	Normal and alloxan-induced diabetic rats	Reduced their plasma glucose levels by 70%	[342]
		Powder	Bulbs	12.5% for 15 days	Normal and HFD alloxan-induced diabetic rats	Reduced fasting blood glucose level	[343]
	*Allium sativum*	Aqueous extract	Bulbs	1250 µg/mL	α-amylase inhibition assay	Inhibitory activity of α-amylase	[344]
		Oil	Bulbs	5–10%	α-amylase inhibition assay	Inhibitory activity of α-amylase	[346]
		Polysaccharide	Bulbs	0.5–4.0 mg/mL	α-amylase and α-glucosidase inhibition assay	Inhibitory activity of α-amylase and α-glucosidase	[347]
		Powder	Bulbs	Nd	Convective hot-air drying α-amylase and α-glucosidase inhibition assay	Inhibitory activity of α-amylase and α-glucosidase	[348]
		Allyl methyl sulfide	Bulbs	50–200 mg/kg for 30 days	STZ-induced diabetic rats	Reduced blood glucose level Regulate insulin production and sensitivity in pancreatic β-cells	[349]
		Ethanolic extract	Bulbs	0.1–0.5 g/kg for 14 days	Normal and STZ-induced diabetic rats	Decreased serum glucose level	[350]
		Aqueous extract	Bulbs	500 mg/kg for 3 weeks	STZ-induced diabetic rats	Decreased serum glucose level	[351]
		Polysaccharide	Bulbs	1.25–5.0 g/kg for 5 weeks	STZ-induced diabetic rats	Reduced fasting blood glucose	[352]
		Aqueous extract	Bulbs	300 μL 200–400 mg/kg for 4 weeks	α-amylase inhibition assay Oral glucose tolerance Alloxan-induced diabetic rats	Inhibitory activity of α-amylase Decreased serum blood glucose level Increased plasma insulin level	[345]
		Aqueous extract	Bulbs	0.4 g/100 g for 4 weeks	Normal and alloxan-induced diabetic rats	Reduced their plasma glucose levels by 68%	[342]
		Powder	Bulbs	12.5% for 15 days	Normal and HFD alloxan-induced diabetic rats	Reduced fasting blood glucose level	[343]
Asteraceae	*Artemisia herba-alba Asso*	EO	Whole plants	0.25–1 mg/mL	α-amylase and α-glucosidase inhibition assay	Inhibitory activity of α-amylase and α-glucosidase	[353]
		Ethyl alcohol extract	Whole plants	200 µL 500–4000 mg/kg	α-amylase inhibition assay Alloxan-induced diabetic rats	Inhibitory activity of α-amylase Decreased plasma glucose level	[354]
		Aqueous extract	Aerial parts	0.39 g/kg for 18 weeks	Alloxan-induced diabetic rats	Reduced blood glucose level	[355]
		Aqueous extract	Aerial parts	100–300 mg/kg for 15 days	Normal and alloxan-induced diabetic rats	Reduced blood glucose level	[356]
		Aqueous extract	Aerial parts	85 mg/kg	STZ-induced diabetic rabbits	Reduced blood glucose level	[357]
		Ethyl alcohol extract	Aerial parts	100–400 mg/kg for 14 weeks	STZ-induced diabetic rats	Reduced fasting blood glucose level Increased plasma insulin level	[358]
		Aqueous extract	Aerial parts	50 and 100 mg/kg	STZ-induced diabetic rabbits	Reduced blood glucose level	[359]
		Aqueous extract	Whole plants	50–100% for 10 days	Dexamethasone-induced diabetic rats	Decreased postprandial blood glucose	[360]
		Hydroethanolic extract	Aerial parts	2 g/kg 18 weeks	HFD-induced diabetic rats	Decreased the blood glucose level and serum insulin concentrations	[361]
		Aqueous extract	Aerial parts	0.39 g/kg for 14 weeks	Alloxan-induced diabetic rats	Reduced fasting serum glucose level	[362]
		Aqueous extract	Aerial parts	400 mg/kg for 3 weeks	Alloxan-induced diabetic rabbits	Reduced blood glucose level	[363]

#### 3.4.1. *Trigonella foenum-graecum*

Fenugreek is known to have various pharmacological effects, such as antibacterial, anticancer, antidiabetic, antioxidant, anticarcinogenic, gastric stimulant, lactation aid, and galactogogue activities. The antidiabetic effect of fenugreek was investigated widely by four studies *in vitro* [235,236,237,238] and eight *in vivo* [239,240,241,242,243,244,245,246]. An *in vitro* study using albino rats showed that fenugreek extract exhibited a maximum α-glucosidase-inhibitory activity at 100 μg/mL (IC_50_ = 57.25 μg/mL) compared to acarbose (STD). Additionally, at 320 μg/mL, the extract demonstrated dipeptidyl peptidase IV (DPP IV) inhibition (IC_50_ = 52.26 μg/mL) [235]. Recently, Neagu and his collaborators investigated the inhibitory effect of fenugreek seeds extract on the enzymatic activity of α-amylase and α-glucosidase. This extract showed the potent inhibition of both enzymes with IC_50_ = 3.22 ± 0.30 μg/mL and 11.14 ± 0.90 μg/mL, respectively [236]. Similar results were obtained in the study done by Laila et al. [237], who reported that the aqueous extract of 4^th^-day-germinated genotype fenugreek sprouts in the form of lyophilized powder (IM6E) also demonstrated strong α-glucosidase activity, and moderate α-amylase and invertase inhibition activities. Using the oral glucose tolerance test (OGTT), the ethanolic extract of fenugreek seeds administrated at 2 g/kg, caused a significant reduction in blood glucose levels of albino rats, which correlates with the α-glucosidase and DPP IV inhibition [239].

Fenugreek water seed extract was found to increase body weight and decrease fasting blood glucose in STZ-induced diabetic rats [239]. These findings are similar to those obtained by Abdelatif et al. [240], who observed weight gain in fenugreek-treated rabbits compared to the group that received only alloxan monohydrate. Plasma glucose levels were also reduced in the fenugreek-treated rabbits. Further, the same extract showed significant antidiabetic activity, with the most effective dose being 1 g/kg, and no acute toxicity was observed when the extract was administered orally at high doses [241]. In another study, fenugreek seed mucilage (FSM) showed antidiabetic actions in streptozotocin-induced diabetic rats (STZ), with FSM being more effective than other plants in ameliorating the diabetic state [242]. The aqueous extract of fenugreek seeds administered at 100 mg/kg significantly reduced blood glucose levels in a diabetic rat model induced by STZ. Urea levels decreased following daily intraperitoneal injection [242]. Fenugreek seed extract reduced blood glucose levels, potentially due to its high content of alkaloid trigonelline and steroidal saponins, particularly the 4-hydroxyisoleucine compound known to be insulinotropic [243]. The hydroalcoholic extract of fenugreek administered at 400 mg/kg body weight significantly decreased blood glucose levels compared to the standard drug glibenclamide [244]. Additionally, three weeks of treatment with insulin and fenugreek seed powder (FSP) separately resulted in a significant reduction in hyperglycemia in diabetic rats. FSP treatment increased insulin levels in diabetic rats to nearly 80% of the control levels [245].

#### 3.4.2. *Nerium oleander*

*N. oleander* has various biological activities, such as antidiabetic, antibacterial, anti-inflammatory, anticancer, antinociceptive, and central nervous system-depressant. The antidiabetic activity of *N. oleander* has been extensively studied across different parts of the plant [246,247,248,249,250,251,252,253]. The enzyme α-amylase is crucial in the breakdown of starch into maltose, maltotriose, and various oligoglucans, which are further converted to glucose by α-glucosidase [246]. *N. oleander* has demonstrated inhibitory activity against α-glucosidase, as shown by Ishikawa et al. [247], who also identified chlorogenic acid as an active isolate. Additionally, Dey et al. [248] investigated the effect of a standardized hydromethanolic extract of *N. oleander* leaves administrated at 200 mg/kg in alloxan-induced diabetic mice. This extract showed a high inhibitory activity against α-amylase (22.63 µg/mL) with an IC_50_ value of 703.01 ± 56.47 mg/mL, and demonstrated significant antihyperglycemic activity, reducing blood glucose levels by 73.79% after 20 days of treatment. The OGTT results reveal a 65.72% decrease in blood glucose levels three hours post-treatment [248]. Similarly, Magdalene et al. [249] reported the concentration-dependent inhibition of α-amylase, leading to decreased blood glucose levels.

The *in vivo* antidiabetic potential of *N. oleander* was also explored by Mwafy et al. [250], who compared the effects of the extract at 250 mg/kg for four weeks on insulin and glucose levels. The results show that the plant extract improved insulin and glucose levels in STZ-induced diabetic rats. Additionally, the ethanolic extract led to a significant decrease in glucose levels and an increase in insulin levels [251]. Furthermore, the administration of *N. oleander* distillate at 375 μg/0.5 mL for 12 weeks to high-fat-diet (HFD)-fed STZ-induced diabetic rats increased insulin sensitivity and the normalization of insulin resistance assessed by a homeostasis model [252]. Ishikawa et al. [247] observed that a very high dose of 16 g/kg lowered blood glucose levels in maltose and sucrose-loaded rats, although it had no effect on glucose loading. Another study confirmed the antihyperglycemic effect of N. oleander extract [248]. In contrast, Sikarwar et al. [253] reported no sub-acute glucose reduction using the *N. oleander* aqueous extract.

#### 3.4.3. *Rosmarinus officinalis*

Rosemary is well-known for its various pharmacological properties, including antidiabetic, anti-inflammatory, antidepressant, antinociceptive, antifungal, and antibacterial activities. Numerous studies have demonstrated the inhibitory effects of *R. officinalis* on key enzymes involved in carbohydrate metabolism, such as α-amylase and α-glucosidase. Numerous studies reported that rosemary EO or aqueous extract is a potent inhibitor of α-amylase (26.29%) and α-glucosidase (75%) [254,255]. Similarly, McCue et al. [256] demonstrated that pure rosmarinic acid extract inhibited α-amylase activity by 85%. Supporting these findings, Belmouhoub et al. [257] demonstrated that diethyl ether and n-butanol fractions of rosemary showed potent α-glucosidase inhibition, with maximum inhibition rates of 77% and 72% at 250 μg/mL, respectively. Further research by Koga et al. [258] identified a rosemary-distilled extract as a strong inhibitor of α-glucosidase, with an IC_50_ value between 683 and 711 μg/mL.

*In vivo* studies have confirmed rosemary’s antidiabetic potential through various models of diabetes. Kabubi et al. [259] demonstrated that a diet supplemented with 12% rosemary leaf powder significantly reduced fasting blood glucose (FBG) levels in diabetic animals, suggesting a hypoglycemic effect comparable to normal control groups. The study attributed this effect to the flavonoid content present in the extracts. Further evidence has been provided by Belmouhoub et al. [257], who evaluated the *in vivo* effects of rosemary fractions in STZ-induced diabetic rats. Their findings reveal that the n-butanol fraction significantly lowered postprandial hyperglycemia, reducing glucose levels by up to 40.77% and 28.2% with sucrose and maltose, respectively. Additionally, the OGTT revealed the maximum antihyperglycemic effect (51.65%) of the n-butanol fraction, which also significantly inhibited glucose intestinal transport.

Moreover, studies on rosemary’s hypoglycemic activity show that its extracts effectively lower glucose levels and improve insulin response. For instance, Benkhedir et al. [260] reported that an ethyl acetate extract of rosemary significantly increased serum glucose and decreased plasma insulin in diabetic control rats. Meanwhile, Khalil et al. [261] observed that the daily administration of aqueous rosmarinic acid at 200 mg/kg for three weeks reduced blood glucose levels. Similar effects were observed with aqueous rosemary extract (ARE), including significant reductions in the fasting plasma glucose (FPG) level in STZ-induced diabetic rats [262]. Supporting these findings, Alnahdi [263] demonstrated that ARE administered at 200 mg/kg/day two weeks before and three weeks after STZ injection significantly reduced FPG [264]. Furthermore, Soliman [265] showed that dried rosemary leaves (5 g/100 g diet) administered for six weeks decreased FPG level in a diabetic group. ARE also provided significant protection against pancreatic β-cell loss, leading to reduced blood glucose levels and increased insulin [266,267,268]. Further studies confirmed these findings by showing that rosmarinic acid dose-dependently decreased plasma glucose levels and improved insulin sensitivity in STZ- and HFD-induced diabetic rats [269,270]. Moreover, in alloxan-induced diabetic models, Bakırel et al. [271] and Kensara et al. [272] provided evidence of rosemary’s efficacy, demonstrating significant reductions in FPG and improvements in insulin levels, and providing renoprotective effects by inhibiting glomerular hypertrophy and glomerulosclerosis [273].

#### 3.4.4. *Salvia officinalis*

*S. officinalis* (sage) is widely recognized for its medicinal properties, including antioxidant, antibacterial, hypoglycemic, anti-inflammatory, fungistatic, and virustatic effects, among others, due to its rich phytochemical content [281,282]. The *in vitro* antidiabetic potential of sage has been demonstrated in various studies. For instance, the EO of sage was found to effectively inhibit the enzymatic activities of α-amylase and α-glucosidase. Al-Mijalli et al. [274] reported that EO exhibited important enzymes inhibitory of α-amylase (IC50 = 81.91 ± 0.03 μg/mL) and α-glucosidase (IC50 = 113.17 ± 0.02 μg/mL), compared to acarbose. Similarly, EO showed the potent inhibition of α-glucosidase in a concentration-dependent manner [275]. Moreover, the aqueous extract showed the inhibition of α-glucosidase (EC50 = 71.2 ± 5.0 µg/mL) at a level four times greater than acarbose [276]. In other studies, the hydroethanolic extracts strongly inhibited α-glucosidase [112,277], while the ethyl acetate fraction exhibited the strong inhibition of both α-amylase (IC50 = 46.52 ± 2.68 mg/mL) and α-glucosidase (104.58 ± 0.06 mg/mL) [278].

*In vivo* studies also support the antidiabetic potential of sage. Moradabadi et al. [279] found that the oral administration of a methanolic extract of sage leaves (500 mg/kg) to alloxan-induced diabetic rats significantly reduced postprandial blood glucose levels, similarly to acarbose. The study further highlighted the short-term blood glucose reduction effects of the extract. Similarly, several authors reported that ethanolic extracts of sage leaves led to significant reductions in blood glucose levels and increased plasma insulin in diabetic rats [280,281,282,283]. These authors confirmed the hypoglycemic effects of sage, which were attributed to its bioactive compounds such as polyphenols, flavonoids, tannins, and alkaloids. Moreover, Mbiti et al. [284] investigated the hypoglycemic effects of the aqueous extracts of sage leaves in alloxan-induced diabetic mice. The results show that the oral administration of this extract significantly lowered FBG levels [284,285]. It is also reported that sage leaves possess a hypoglycemic effect on STZ-induced diabetic rats [286]. Both *in vitro* and *in vivo* studies substantiate the antidiabetic properties of sage, emphasizing its role in inhibiting key digestive enzymes and reducing blood glucose levels in diabetic models.

#### 3.4.5. *Marrubium vulgare*

*M. vulgare* is known for its diverse medicinal properties, including hypoglycemic, vasorelaxant, analgesic, antioxidant, anti-inflammatory, vasodilator, and antihypertensive activities. The antihyperglycemic potential of *M. vulgare* has been well documented. Gourich et al. [287] demonstrated that the administration of *M. vulgare* extract effectively reduced elevated glucose levels, comparable to the effect of glibenclamide. The study also highlighted the extract’s significant inhibitory effect on pancreatic α-amylase activity, with an IC50 value of 0.081 ± 0.013 mg/mL, outperforming acarbose. This inhibition is likely due to the presence of bioactive compounds within the extract. Similar results have been observed by Aazza et al. [288], who reported that the hydro-alcoholic extract exhibited the most potent α-amylase inhibition among six studied plants.

A series of *in vivo* experiment were conducted on different models to determine the antidiabetic effects of *M. vulgare*. In studies on STZ-induced diabetic rats, the methanolic extract of the aerial parts was shown to have a beneficial effect on diabetes and its complications. Moreover, a daily oral dose of 500 mg/kg for 28 days resulted in a significant reduction in blood glucose from the second week, along with increased plasma insulin and tissue glycogen levels [289]. The study suggests that the extract’s antidiabetic effects may be linked to the stimulation of insulin release from the remaining pancreatic beta cells. Another study explored the effects of the methanol, water and butanol extracts of the whole plant on autoimmune diabetes mellitus type 1 induced by cyclosporine A and STZ in mice, demonstrating its potential therapeutic benefits [290]. In an alloxan-induced diabetic rats model, Boudjelal et al. [214] reported that aqueous extracts from the aerial parts (100, 200, and 300 mg/kg) resulted in a dose-dependent reduction in blood glucose levels—up to a 60% decrease at higher doses. Similarly, the aqueous extract of the leaf infusion improved blood glucose levels, indicating its protective effects against diabetes-related complications [291]. Vergara-Galicia et al. [292] investigated the antidiabetic activity of various ethanolic extracts of the whole plant on normoglycemic rats. The intragastric administration of the whole plant extract (100 mg/kg) significantly reduced blood glucose levels and suppressed any elevation in plasma glucose.

#### 3.4.6. *Olea europaea*

*O. europaea* has a wide range of medicinal properties and traditional uses, including antihypertensive, antidiabetic, antioxidant, and anti-inflammatory activities. Several studies have demonstrated the antidiabetic effects of olive extracts in different models. In STZ-induced diabetic rats, alcohol extracts significantly decreased blood glucose levels at doses of 0.1, 0.25, and 0.5 g/kg administrated over 14 days, showing greater efficacy than glibenclamide [293,294]. This effect is consistent across various studies [295,296,297,298,299,300,301,302,303,304,305], suggesting a strong hypoglycemic potential. Mansour et al. [305] reported that the administration of olive extract combined with metformin significantly reduced blood glucose levels to near-normal levels, indicating its potential as an adjuvant therapy. Similarly, Wainstein et al. [295] demonstrated improved glucose homeostasis with repeated administration. Furthermore, Shudiefat et al. [304] suggested that olive extract exerted antihyperglycemic effects through AS160 inhibition, offering an alternative to metformin treatment. The antidiabetic potential of oleanolic acid, isolated from olive leaves, was also confirmed, showing a reduction in blood glucose and insulin levels in HFD mice [306]. The antidiabetic effects extend to alloxan-induced diabetic models as well. Olive leaf extracts have shown significant reductions in blood glucose in rats [307,308,309,310] and rabbits [311,312]. Al-Azzawie et al. [312] studied the hypoglycemic activity of hydroxytyrosol from olive leaves in diabetic rabbits, and found that oleuropein had significant hypoglycemic activity due to its antioxidant potential. Farah et al. [311] investigated the effects of ethanolic olive leaf extracts, with the maximum hypoglycemic activity observed at a dose of 600 mg/kg. The hypoglycemic effect of olive leaf extracts is extensively related to improvements in oxidative stress markers, further supporting its potential as a natural antidiabetic treatment [307,309,310,312].

Recent studies have focused on the α-glucosidase-inhibitory effects of olive leaf extracts (OLEs), which could help explain how they lower blood sugar and create safer, more natural antidiabetic supplement alternatives. Mansour et al. [305] reported strong α-glucosidase inhibitory activity in all studies on OLEs, with inhibition increasing with concentration. AlShaal et al. [313] observed that olive leaf extracts inhibited α-glucosidase by 81.34% at 3.85 mg/mL, with an IC50 of 0.34 ± 0.12 mg/mL. The hydroxytyrosol and oleuropein in olive leaves showed potent α-glucosidase-inhibitory effects compared to α-amylase, as demonstrated by Hadrich [134], with IC50 values of 150 µM and 400 µM, respectively. The role of phenolic compounds in OLEs was highlighted by Loizzo et al. [314], who showed that olive oil extracts were weaker inhibitors of α-amylase compared to α-glucosidase (IC50 = 258 and 184 mg/mL, respectively). Khlif et al. [315] further showed that oleanolic acid and its dimethyl derivative from olive stems were active against α-amylase enzyme, with IC50 values of 1.18 and 1.03 mg/mL, respectively. Numerous *in vitro* studies, such as those by Mansour [315], suggest that plant polyphenols in OLEs could inhibit carbohydrate hydrolytic enzymes by binding to the proteins, thus delaying the hydrolysis and absorption of monosaccharides.

#### 3.4.7. *Nigella sativa*

*N. sativa* seeds and their oil possess various medicinal properties, including potent antidiabetic activity. Several studies have demonstrated the hypoglycemic effects of *N. sativa* in different models of diabetes. For instance, Alhodieb et al. [316] found that black seed extract inhibited α-glucosidase in a dose-dependent manner, which can be attributed to the presence of compounds like ferulic acid, rutin, and catechin. Using the oral glucose tolerance test, the aqueous and ethanolic extracts of *N. sativa* seeds demonstrated significant hypoglycemic and hypolipidemic effects, all without any observed toxicity [228,317].

Research on alloxan-induced diabetic rats also underscores the antidiabetic potential of *N. sativa*. The administration of methanolic crude extract and commercial oil of *N. sativa* seeds resulted in significant blood glucose reductions [318,319,320]. Similarly, Sutrisna et al. [321] found that the ethyl acetate fraction of ethanolic extract reduced blood glucose levels. In STZ-induced diabetic rats treated with *N. sativa* seed extract, serum glucose levels decreased considerably compared to diabetic controls [322,323,324,325,326]. Treatment for six weeks resulted in hypoglycemic effects and improved cardiovascular complications associated with diabetes (Abbasnezhad, 2019). For instance, Fararh et al. [327] and Abdelrazek et al. [328] showed that the oral administration of *N. sativa* oil led to a significant, consistent, and time-dependent decrease in blood glucose levels in STZ-induced diabetic hamsters. Additionally, Le et al. [329] showed that petroleum ether extract enhanced insulin signaling pathways in STZ-induced diabetic rats. In another study conducted by Dong et al. [330], they found that *N. sativa* seed polysaccharides significantly reduced FBG levels and increased insulin levels. These studies reveal that NS in various forms—oil, water extracts, dried seeds—exhibits substantial hypoglycemic potential, particularly in forms based on aqueous extraction.

#### 3.4.8. *Allium cepa*

Recent studies have highlighted the diverse biological properties of onion, including its antihypertensive, antioxidant, antimicrobial, anti-inflammatory, and antidiabetic effects. In particular, the antidiabetic potential of onion and its extracts has been extensively investigated through both *in vitro* and *in vivo* studies. The *in vitro* antidiabetic potential of onion skin (OS) extract has been well documented. For instance, the extract showed significant inhibitory activity against α-glucosidase and α-amylase, with IC_50_ values of 1.27 mg/mL and >3.00 mg/mL, respectively [331]. Methyl alcohol extracts have also been reported to inhibit yeast α-glucosidase with an IC_50_ value of 0.159 mg/mL [332]. Quercetin, a key compound in onion extract, exhibited potent sucrose-inhibitory activity (IC_50_ = 0.11 mg/mL), suggesting its role as an active component [331]. Quercetin’s inhibition of α-glucosidase helps delay glucose absorption, aiding in the control of blood glucose levels. The ethanolic extract has also shown promising antidiabetic effects by inhibiting α-amylase and α-glucosidase activities, with inhibition increasing with concentration. At 30 µL, both the extract and the standard drug demonstrated a 75% inhibition rate, which increased to 80% at 50 µL [333]. Further research by Gois Ruivo da Silva et al. [334] revealed that 50% and 100% ethanol extracts, and 100% methanol extracts, of OS, at concentrations ranging from 0.01 to 10 mg/mL, effectively decreased α-amylase activity. Interestingly, OS extract exhibited higher inhibition than the quercetin standard, indicating that additional substances in OS may synergistically contribute to this effect. Both yellow and red OS extracts (ethanolic and aqueous) also demonstrated dose-dependent inhibitory activity against α-glucosidase (IC_50_ = 3.90–8.99 μg/mL) [335]. Nile et al. [336] confirmed that various extracts of red OS waste displayed enzyme-inhibitory effects against α-glucosidase (IC_50_ = 42.8–73.2 μg/mL), with methanol and ethanol extracts being the most effective. The study also noted that flavonoid glucosides extracted from red OS could be used to treat diabetes mellitus, hyperuricemia, and skin pigmentation disorders.

The antidiabetic effects of onion have also been observed in *in vivo* studies. El-Soud and Khalil [337] reported that onion EO treatment led to significant decreases in blood glucose and increases in serum insulin in STZ-induced diabetic albino rats. Similarly, red onion extract reduced FBG levels and increased serum insulin levels [338]. Jung et al. [339] explored the effects of OS extract on hyperglycemia and insulin sensitivity in HFD/STZ-induced diabetic rats. The administration of 1% OS led to a significant decrease in the incremental area under the curve and improved insulin sensitivity. The study found that 1% OS had a stronger hypoglycemic effect than pure quercetin, likely due to the presence of over 20 other flavonoids. Similarly, Islam et al. [340] demonstrated that serum insulin concentrations and insulin resistance were dose-dependently increased in onion-fed groups compared to diabetic control groups. The hypoglycemic effects of onion were further confirmed in alloxan-induced diabetic rat models, where aqueous extracts reduced FBG levels by 75.4% at 300 mg/kg [341]. Another study reported significant antihyperglycemic effects following 4 weeks of onion juice treatment [342]. Gholamali et al. [343] observed that onion consumption led to significant reductions in FBG, aligning with findings by Abouzed et al. [338] and Ozougwu et al. [341] that suggest onion acts as a hypoglycemic agent. Collectively, these studies underscore the antidiabetic potential of onion and its extracts, with phenolic compounds like quercetin and other flavonoids playing a crucial role in their efficacy.

#### 3.4.9. *Allium sativum*

Commonly known as garlic, this plant is widely recognized not only as a food flavor-enhancer, but also for its medicinal properties, including its use in managing diabetes. Several studies have highlighted the significant inhibitory effects of garlic extracts on enzymes such as α-amylase and α-glucosidase, which are crucial in carbohydrate digestion. For instance, an ethanolic extract of garlic bulbs exhibited an 81.86% inhibition of α-amylase at 1250 µg/mL [344]. The inhibitory effect of garlic extract on α-amylase was also shown to be highly effective, with an IC_50_ of 680.54 ± 0.58 μg/mL—significantly more potent than the standard drug acarbose [345]. Moreover, a further study demonstrated that oil extracted from garlic bulbs had a stronger inhibitory activity on α-amylase than other species of the Allium genus, with an IC_50_ value of 3.0 ± 0.02% [346]. Yan et al. [347] also observed that polysaccharides extracted from garlic bulbs significantly inhibited both α-amylase and α-glucosidase in a dose-dependent manner, with the strongest inhibition attributed to a high uronic acid content and low molecular weight fractions. Additionally, another study investigated the effects of a convective hot-air drying method on garlic’s enzyme-inhibitory α-amylase and α-glucosidase properties [348]. These authors found that garlic’s extracted compounds could serve as functional ingredients in dietary treatments for early-stage hyperglycemia.

*In vivo* studies further support these findings. Sujithra et al. [349] demonstrated that doses of 50, 100, and 200 mg/kg of garlic effectively reduced blood glucose levels and regulated insulin production and sensitivity in STZ-induced diabetic rats. Similarly, the oral administration of garlic extract normalized serum glucose and insulin levels in both normal and diabetic rats, with effects that were even more notable than glibenclamide [350,351]. Moreover, the FBG in the high-dose polysaccharide group was 42% lower than in the diabetic model group, demonstrating its hypoglycemic effect [352]. Gholamali et al. [343] and El-Demerdash et al. [342] also reported that garlic consumption significantly decreased FBS in HFD alloxan-induced diabetic rats, possibly due to the actions of compounds like allyl propyl disulfide or diallyl disulfide. The aqueous extract of garlic bulbs (200 and 400 mg/kg) has been shown to increase plasma insulin. Notably, these extracts significantly reduced blood glucose levels during the OGTT, outperforming the acarbose molecule in reducing postprandial glycemia [345]. These studies suggest that garlic, due to its enzyme-inhibitory properties and hypoglycemic effects, is a promising agent for managing diabetes, particularly in the early stages of hyperglycemia.

#### 3.4.10. *Artemisia herba-alba Asso*

Numerous studies have demonstrated that *A. herba-alba* (AHA) exhibits a wide range of biological and pharmacological effects, particularly regarding its antibacterial, antispasmodic, antidiabetic, antioxidant, leishmanicidal, and antifungal properties. Regarding its antidiabetic potential, the EO of AHA has shown strong inhibitory activity against α-amylase and α-glucosidase enzymes, with IC50 values of 1.946 and 1.754 mg/mL, respectively [353]. Similarly, Awad et al. [354] emphasized the hypoglycemic activity of AHA *in vitro*, noting that the 70% ethyl alcohol extract and its mucilage inhibited α-amylase activity by 11% and 2%, respectively.

Further supporting these findings, Taştekin et al. [355] observed that the aqueous extract of AHA significantly reduced blood glucose concentrations in alloxan-induced diabetic rats, an effect comparable to that of insulin and repaglinide. This hypoglycemic effect was further confirmed by Boudjelal et al. [356], who found that the oral administration (300 mg/kg) of AHA aqueous infusions resulted in a significant reduction in blood glucose levels, demonstrating more efficacy than glibenclamide [354]. These results underscore the plant’s traditional use as an antidiabetic remedy. In another study, Iriadam et al. [357] demonstrated that the oral administration of AHA aqueous extract significantly reduced blood sugar levels in both normal and diabetic rabbits, indicating its potential for broad-spectrum hypoglycemic activity. Abdallah et al. [358] also reported that ethyl alcohol extracts of AHA at various concentrations significantly decreased FBG and homocysteine levels, while enhancing plasma insulin in STZ-treated rats, with similar effects observed in studies by El-Marasy et al. [359]. Ahmad et al. [360] further corroborated these findings, showing that AHA’s aqueous extract has potent hypoglycemic effects in experimentally induced hyperglycemic rats. Complementing this, Hamza et al. [361] demonstrated that a dose of hydro-alcoholic extracts of AHA (2 g/kg), administered orally for 18 weeks, significantly lowered blood glucose levels and serum insulin concentrations in male mice fed a high-fat diet. These results align with those of previous studies on the hypoglycemic effects of AHA in diabetic rats [355,362], rabbits [363] and normal mice [361].

Based on the phytochemical and pharmacological literature reviewed in this study, the most promising antidiabetic plants include *T. foenum-graecum*, *O. europaea*, *N. Sativa*, *A. herba-alba*, and *S. officinalis*. These species demonstrate strong *in vivo* and *in vitro* antidiabetic effects, often attributed to their high contents of bioactive compounds such as flavonoids, terpenoids, and phenolic acids.

- *T. foenum-graecum*: Numerous studies have demonstrated its hypoglycemic potential, attributed to its saponins, alkaloids, and flavonoids. Clinical trials also show its promise in improving glucose tolerance.
- *O. europaea*: The leaves contain high levels of oleuropein and hydroxytyrosol, known for their antidiabetic properties. These compounds have shown potent effects in animal models of diabetes.
- *N. sativa*: Thymoquinone and other phenolics demonstrate strong insulinotropic and glucose-lowering effects *in vivo*.
- *A. herba-alba*: The plant is rich in terpenoids, particularly thujone and camphor, which have shown antidiabetic effects in animal models. Its use in North Africa is well-established, and its traditional use is supported by modern pharmacological studies.
- *S. officinalis*: This plant is widely recognized for its high levels of rosmarinic acid and flavonoids, which exhibit both hypoglycemic and antioxidant properties. *In vivo* studies confirm its potential as an adjunct in diabetes management.

These species should be prioritized in future research, focusing on their mechanisms of action, dosage optimization, and potential synergistic effects when combined with conventional treatments.

### 3.5. Current Therapeutic Trajectory of Diabetes Management in Morocco

The current landscape of diabetes management in Morocco predominantly involves conventional pharmacological treatments, such as insulin and oral hypoglycemic agents (e.g., metformin, sulfonylureas), commonly prescribed for type 2 diabetes [364]. These therapies, while effective, can have significant side effects and limitations, including hypoglycemia, weight gain, and long-term cardiovascular risks [365]. As a result, the World Health Organization (WHO) has long advocated for integrating Traditional Medicine (TM) into modern healthcare, offering a more holistic, sustainable, and culturally acceptable approach to manage chronic diseases like diabetes [366].

In Morocco, traditional medicinal plants are increasingly being explored for their potential to complement standard therapies. Several plant species, including *T. foenum-graecum*, *N. sativa*, *R. officinalis*, and *O. europaea*, have demonstrated significant hypoglycemic effects in both *in vitro* and *in vivo* studies. These plants often enhance or mimic the effects of conventional treatments. For instance, *T. foenum-graecum* improves insulin sensitivity and secretion, while *R. officinalis* exhibits strong antioxidant properties that may help mitigate oxidative stress associated with diabetes.

Given the WHO’s recommendation to integrate TM into modern healthcare, these plants offer a cost-effective and culturally appropriate complement to pharmaceutical drugs. In rural Moroccan communities, patients frequently use these medicinal plants alongside conventional treatments, further underscoring their practical potential in bridging traditional knowledge with modern medicine [367]. However, structured clinical trials are essential to evaluate the safety, dosage, and interactions with modern hypoglycemic drugs of these plants, so as to ensure their safe integration into diabetes management.

### 3.6. Comparison with Plant-Based Management of Diabetes in the Maghreb Region

In the Maghreb region, including Algeria, Tunisia, and Libya, plant-based diabetes management shows many similarities with that in Morocco, largely due to the shared ecological and cultural contexts. Common medicinal plants used across these countries include *T. foenum-graecum*, *N. sativa*, *R. officinalis*, *O. europeae* and *A. herba-alba.* Despite these commonalities, local traditions and the availability of specific plants introduce variations in usage. For example, *A. herba-alba* is more widely studied in Morocco, while combinations of plants are frequently used in Tunisia and Algeria [368,369]. Nevertheless, Libya shows a limited number of studies compared to Morocco, but ethnobotanical research suggests that *T. foenum-graecum*, *O. europeae*, *M. vulgare*, *S. officinalis* and *A. herba-alba* are common across the Maghreb for their antidiabetic properties [370,371].

Fenugreek’s antidiabetic properties are well documented throughout the Maghreb. In Morocco, fenugreek has been used traditionally for its hypoglycemic effects, supported by modern research showing its ability to improve insulin sensitivity and lower blood sugar levels [235,236,237,238,239,240,241,242,243,244,245]. In Algeria, similar studies demonstrate its potential in enhancing glucose tolerance and exerting insulinotropic effects in diabetic rats [372]. In Tunisia, a study by Hachouf et al. [373] corroborated these findings, showing that fenugreek enhances insulin secretion, aligning with Moroccan and Algerian results. Fenugreek seeds contain alkaloids and flavonoids, which contribute to its hypoglycemic action across the region. *N. sativa*, is another plant extensively used in Maghreb traditional medicine for diabetes management. In Algeria, Houcher et al. [318] conducted *in vivo* studies that showed its significant hypoglycemic and insulin-sensitizing effects. Tunisian research by Ghlissi et al. [374] confirmed these results, noting that black seed not only regulates glucose metabolism, but also exerts antioxidant effects. These findings align with *N. sativa*’s traditional use in Morocco, and support its importance across the region in managing diabetes. Rosemary is widely used for its antidiabetic and antioxidant properties across the Maghreb. In Algeria, Benkhedir et al. [260] highlighted its significant ability to reduce hyperglycemia and improve insulin sensitivity in diabetic rats. Rosemary’s bioactive compounds, including flavonoids and phenolic acids, have been reported to lower blood glucose by stimulating insulin secretion from pancreatic cells [375]. These findings align closely with the traditional use of rosemary in Morocco for managing diabetes. Likewise, *S. officinalis* is used in Tunisian folk medicine, often in combination with other herbs for diabetes treatment, which reflects a region-specific approach to herbal synergy that differs from Moroccan practices [376].

The olive tree holds a significant place in the cultural and medicinal landscape of the Maghreb. In Algeria, studies show that olive leaf extracts exhibit strong hypoglycemic and antioxidant effects in diabetic rats [368]. Similar findings are reported in Tunisia, where Wannes and Marzouk [369] highlighted the ability of olive leaves to lower blood glucose levels. These effects are primarily attributed to the presence of oleuropein and other polyphenols that promote insulin sensitivity. In Morocco, olive leaves are used similarly, and the plant is widely recognized for its antidiabetic properties in traditional medicine. *A. herba-alba* is also well-known for its antidiabetic properties across the region. In Algeria, aqueous extracts of this plant have been shown to reduce hyperglycemia and provide antioxidant effects in diabetic rats [368]. Tunisian studies also confirm the plant’s hypoglycemic efficacy, aligning with findings in Morocco [369]. However, its use is somewhat less prominent in Tunisia and Algeria compared to Morocco, where it has been extensively studied and forms a key component of traditional diabetes treatments.

In summary, there is substantial overlap in the use of medicinal plants for diabetes management across the Maghreb, with shared reliance on species like *T. foenum-graecum*, *N. sativa*, *R. officinalis*, *O. europaea*, and *A. herba-alba*. The ecological similarities of these countries contribute to the commonality of plant species, while local traditions and plant availability account for regional variations. Tunisia and Algeria, for instance, use more combinations of plants, while Morocco tends to focus on singular applications of these herbs. Despite these differences, the shared ethnobotanical knowledge highlights the collective cultural importance of plant-based diabetes treatments in the Maghreb.

## 4. Future Directions and Research Opportunities

Future research on the antidiabetic effects of Moroccan medicinal plants should prioritize the standardization of extracts and dosages to ensure consistency in bioactive compound concentrations. Advanced techniques could elucidate the molecular mechanisms through which compounds like saponins and flavonoids exert their antidiabetic effects. Additionally, well-designed clinical trials are critical to evaluate the efficacy and safety of these plants in humans, considering various comorbidities. Investigating the synergistic effects of polyherbal formulations and potential drug–herb interactions is also essential for their safe and effective use.

Comprehensive safety profiling and toxicological assessments are necessary, especially for plants with known risks, such as *N. oleander*. Ethnopharmacological studies should continue to explore new species with antidiabetic potential, ensuring that sustainable practices are employed to conserve these valuable medicinal resources. Further research on isolating and characterizing specific bioactive compounds could lead to the development of novel pharmaceuticals. By addressing these research opportunities, the therapeutic potential of Moroccan medicinal plants for diabetes management could be fully realized, leading to the development of natural-based treatments for this widespread condition.

## 5. Conclusions and Implications for Healthcare Practice

The extensive use of Moroccan medicinal plants in the management of diabetes highlights their potential as alternative or complementary therapies for blood sugar regulation. This review has documented 344 medicinal plant species from 79 different families, with plants from the Compositae family being the most frequently used. Among these, ten of the most effective plants have been identified and reviewed for their *in vitro* and *in vivo* antidiabetic properties. However, while these plants show potential, their effectiveness and safety must be validated through standardized clinical trials. The variability in plant composition, potential toxicity, and interactions with conventional medications necessitate a cautious and well-informed approach in integrating these plants into mainstream healthcare.

For healthcare practitioners, understanding the benefits and risks associated with these medicinal plants is crucial for advising patients, especially those who may seek complementary therapies for diabetes management. Educating patients on the importance of evidence-based use and potential interactions with prescribed medications is essential to prevent adverse effects. Furthermore, ongoing research and collaboration between traditional healers and modern healthcare providers could facilitate the safe and effective incorporation of these plants into treatment regimens, offering patients more holistic and personalized care options.

## Figures and Tables

**Table 2:** The origins of Moroccan medicinal plants used in the treatment of diabetes.

Family Name	Scientific Name	Origin
Aizoaceae	*Opophytum theurkauffii* Maire L.	Spontaneous
Alliaceae	*Allium cepa* L.	Cultivated
	*Allium sativum* L.	Cultivated
	*Allium ampeloprasum var. porrum*	Cultivated
Aloeaceae	*Aloe vera* (L.) *Burm.f.*	Cultivated
Amaranthaceae	*Anabasis aretioides Moq. & Coss*. ex Bunge	Spontaneous
	*Beta vulgaris* L.	Cultivated
	*Spinacia oleracea* L.	Cultivated
Anacardiaceae	*Pistacia atlantica Desf.*	Spontaneous
	*Pistacia lentiscus* L.	Spontaneous
	*Searsia albida* (*Schousb.*) *Moffett*	Spontaneous
Apiaceae	*Ammodaucus leucotrichus Coss.*	Spontaneous
	*Ammi majus* L.	Spontaneous
	*Ammi visnaga* (L.) *Lam.*	Spontaneous
	*Anethum foeniculum* L.	Cultivated
	*Apium graveolens* L.	Cultivated
	*Carum carvi* L.	Cultivated
	*Coriandrum sativum* L.	Cultivated
	*Cuminum cyminum* L.	Cultivated
	*Daucus carota* L.	Cultivated
	*Eryngium ilicifolium Lam.*	Spontaneous
	*Ferula communis* L.	Spontaneous
	*Foeniculum vulgare Mill.*	Cultivated
	*Pastinaca sativa* L.	Cultivated
	*Petroselinum crispum* (Mill.) Fuss	Cultivated
	*Petroselinum sativum Hoffm*	Cultivated
	*Pimpinella anisum* L.	Cultivated
	*Ptychotis verticillata* Duby	Cultivated
	*Ridolfia segetum* (L.) Moris	Spontaneous
Apocynaceae	*Apteranthes europaea* (Guss.) Murb.	Spontaneous
	*Calotropis procera* (Aiton) Dryand.	Spontaneous
	*Caralluma europaea* (Guss.) N.E.Br.	Spontaneous
	*Nerium oleander* L.	Spontaneous
	*Periploca laevigata* subsp. *Angustifolia* (Labill.) Markgr.	Spontaneous
Arecaceae	*Chamaerops humilis* L.	Spontaneous
	*Hyphaene thebaica* (L.) Mart.	Spontaneous
	*Phoenix dactylifera* L.	Cultivated
Aristolochiaceae	*Aristolochia baetica* L.	Spontaneous
	*Aristolochia longa* subsp. *Fontanesii* Boiss. & Reut.	Spontaneous
Asparagaceae	*Agave americana* L.	Cultivated
	*Asparagus albus* L.	Spontaneous
	*Asparagus officinalis* L.	Cultivated
Asteraceae	*Achillea odorata* L.	Spontaneous
	*Achillea santolinoides Lag.*	Spontaneous
	*Anacyclus pyrethrum* (L.) Lag.	Spontaneous
	*Antennaria dioica* (L.) Gaertn	Spontaneous
	*Anvillea garcinii* subsp. *Radiata* (Coss. & Durieu) Anderb.	Spontaneous
	*Artemisia abrotanum* L.	Cultivated
	*Artemisia absinthium* L.	Cultivated
	*Artemisia arborescens* (*Vaill.*) L.	Spontaneous
	*Artemisia atlantica* Coss. & Durieu	Spontaneous
	*Artemisia campestris* L.	Spontaneous
	*Artemisia herba-alba* Asso	Spontaneous
	*Artemisia herba alba Assac.*	Spontaneous
	*Artemisia mesatlantica Maire*	Endemic
	*Artemisia reptans* C. Sm. ex Link	Spontaneous
	*Atractylis gummifera Salzm. ex* L.	Spontaneous
	*Calendula arvensis* Bieb.,	Spontaneous
	*Centaurea maroccana* Bal	Spontaneous
	*Chamaemelum mixtum* (L.) Alloni	Spontaneous
	*Chamaemelum nobile* (L.) All.	Spontaneous
	*Chrysanthemum coronarium* L.	Spontaneous
	*Cichorium intybus* L.	Cultivated
	*Cladanthus arabicus* (L.) Cass.	Spontaneous
	*Cladanthus scariosus* (Ball) Oberpr. & Vogt	Spontaneous
	*Cynara cardunculus* L.	Cultivated
	*Cynara cardunculus* subsp. *scolymus* (L.)	Cultivated
	*Cynara humilis* L.	Spontaneous
	*Dittrichia viscosa* (L.) Greuter	Spontaneous
	*Echinops spinosissimus Turra*	Spontaneous
	*Helianthus annuus* L.	Cultivated
	*Inula conyza* (*Griess.*) *DC.*	Spontaneous
	*Inula helenium* L.	Cultivated
	*Lactuca sativa* L.	Cultivated
	*Launaea arborescens* (Batt.) Murb.	Spontaneous
	*Matricaria chamomilla* L.	Spontaneous
	*Pallenis spinosa* (L.) Cass.	Spontaneous
	*Saussurea costus* (*Falc.*) *Lipschitz*	Spontaneous
	*Scolymus hispanicus* L.	Spontaneous
	*Scorzonera undulata* Vahl	Spontaneous
	*Seriphidium herba-alba*	Spontaneous
	*Sonchus arvensis* L.	Spontaneous
	*Sonchus asper* (L.) *Hill*	Spontaneous
	*Sonchus tenerrimus* L.	Spontaneous
	*Stevia rebaudiana Willd.*	Cultivated
	*Silybum marianum* L.	Spontaneous
	*Tanacetum vulgare* L.	Spontaneous
	*Taraxacum campylodes* G.E. Haglund	Spontaneous
	*Warionia saharae Benthem* ex Benth. & Coss.	Spontaneous
Berberidaceae	*Berberis vulgaris* subsp. *Australis* (Boiss.) Heywood	Spontaneous
Brassicaceae	*Anastatica hierochuntica* L.	Spontaneous
	*Brassica napus* L.	Cultivated
	*Brassica nigra* (L.) K. Koch	Cultivated
	*Brassica oleracea* L.	Cultivated
	*Brassica rapa* L.	Cultivated
	*Diplotaxis pitardiana* Maire	Spontaneous
	*Eruca vesicaria* (L.) Cav.	Spontaneous
	*Lepidium sativum* L.	Cultivated
	*Nasturtium officinale* R.Br.	Spontaneous
	*Ptilotrichum spinosum* (L.) Boiss.	Spontaneous
	*Raphanus raphanistrum* subsp. *sativus* (L.)	Cultivated
Burseraceae	*Boswellia sacra* Flueck.	Imported
	*Commiphora myrrha* (*Nees*) *Engl.*	Cultivated
Buxaceae	*Buxus balearica Lam.*	Cultivated
	*Buxus sempervirens* L.	Cultivated
Cactaceae	*Opuntia ficus indica* (L.) *Mill.*	Spontaneous/Cultivated
Capparaceae	*Capparis decidua* (*Forssk.*) *Edgew.*	Cultivated
	*Capparis spinosa* L.	Spontaneous
	*Maerua crassifolia Forssk.*	Cultivated
Caryophyllaceae	*Herniaria glabra var. hirsuta* (L.) *Kuntze*	Spontaneous
	*Paronychia argentea* Lam.	Spontaneous
	*Silene vivianii* Steud.	Spontaneous
	*Corrigiola telephiifolia Pourr.*	Spontaneous
Cannabaceae	*Cannabis sativa* L.	Spontaneous/Cultivated
Cistaceae	*Cistus albidus* L.	Spontaneous
	*Cistus creticus* L.	Spontaneous
	*Cistus laurifolius* L.	Spontaneous
	*Cistus salviifolius* L.	Spontaneous
	*Cistus ladanifer* L.	Spontaneous
Chenopodiaceae	*Atriplex halimus* L.	Spontaneous
	*Chenopodium ambrosioides* L.	Spontaneous
	*Hammada scoparia* (Pomel) Iljin	Spontaneous
	*Salsola tetragona* Delile	Spontaneous
	*Suaeda mollis* Dest.,	Spontaneous
Colchicaceae	*Androcymbium gramineum* (Cav.) J.F. Macbr.	Spontaneous
Convolvulaceae	*Ipomoea batatas* (L.)	Cultivated
Cucurbitaceae	*Bryonia dioica Jacq.*	Spontaneous
	*Citrullus colocynthis* (L.) Schrad.	Spontaneous
	*Citrullus vulgaris Schard.*	Cultivated
	*Cucumis sativus* L.	Cultivated
	*Cucumis melo var. flexuosus* L.	Cultivated
	*Cucurbita maxima Duchesne*	Cultivated
	*Cucurbita pepo* L.	Cultivated
Cupressaceae	*Juniperus phoenicea* L.	Imported
	*Juniperus thurifera L*	Spontaneous
	*Juniperus oxycedrus* L.	Imported
	*Tetraclinis articulata* (Vahl) Mast.	Spontaneous
Cynomoriaceae	*Cynomorium coccineum* L.	Spontaneous
Cyperaceae	*Bolboschoenus maritimus* (L.) *Palla*	Spontaneous
	*Cyperus longus* L.	Imported
	*Cyperus rotundus* L.	Spontaneous
Dracaenaceae	*Dracaena draco* subsp. *ajgal* Benabid *&* Cuzin	Cultivated
Ephedraceae	*Ephedra alata* Decne.	Spontaneous
	*Ephedra altissima Desf.*	Spontaneous
	*Ephedra fragilis* Desf.	Spontaneous
Equisetaceae	*Equisetum ramosissimum Desf*	Spontaneous
Ericaceae	*Arbutus unedo* L.	Spontaneous
	*Vaccinium myrtillus* L.	Cultivated
Euphorbiaceae	*Euphorbia officinarum* subsp. *echinus* (Hook. f. & Coss.) Vindt	Spontaneous
	*Euphorbia officinarum* L.	Spontaneous
	*Euphorbia peplis* L.	Spontaneous
	*Euphorbia resinifera* O. Berg	Endemic
	*Mercurialis annua* L.	Spontaneous
	*Ricinus communis* L.	Spontaneous
Fagaceae	*Quercus coccifera* L.	Spontaneous
	*Quercus suber* L.	Spontaneous
	*Quercus ilex* L.	Imported
Gentianaceae	*Centaurium erythraea* Rafn	Spontaneous
	*Centaurium spicatum* (L.) *Fritsch*	Cultivated
Geraniaceae	*Pelargonium odoratissimum*	Cultivated
	*Pelargonium roseum Willd.*	Cultivated
Iridaceae	*Crocus sativus* L.	Cultivated
Juglandaceae	*Juglans regia* L.	Cultivated
Juncaceae	*Juncus maritimus* Lam.	Cultivated
Lamiaceae	*Ajuga iva* (L.) *Schreb.*	Spontaneous
	*Ballota hirsuta Benth*	Spontaneous
	*Calamintha officinalis Moench.*	Spontaneous
	*Calamintha nepeta* subsp. *Spruneri* (*Boiss.*) *Nyman*	Spontaneous
	*Calamintha alpina* L.	Spontaneous
	*Clinopodium alpinum* (L.) *Kuntze*	Spontaneous
	*Clinopodium nepeta* subsp. *glandulosum* (*Req.*) *Govaerts*	Spontaneous
	*Lavandula angustifolia Mill*	Spontaneous
	*Lavandula dentata* L.	Spontaneous
	*Lavandula maroccana Murb.*	Endemic
	*Lavandula multifida* L.	Spontaneous
	*Lavandula stoechas* L.	Spontaneous
	*Marrubium vulgare* L.	Spontaneous
	*Mentha pulegium* L.	Spontaneous
	*Melissa officinalis* L.	Spontaneous
	*Mentha spicata* L.	Spontaneous
	*Mentha piperita* L.	Cultivated
	*Mentha suaveolens Ehrh.*	Spontaneous
	*Ocimum basilicum* L.	Cultivated
	*Origanum compactum Benth.*	Spontaneous
	*Origanum elongatum* (*Bonnet*) *Emb. & Maire*	Spontaneous
	*Origanum majorana* L.	Spontaneous
	*Origanum vulgare* L.	Spontaneous
	*Rosmarinus officinalis* L.	Imported
	*Salvia officinalis* L.	Cultivated
	*Salvia hispanica* L.	Cultivated
	*Teucrium polium* L.	Spontaneous
	*Thymus broussonetii Boiss.*	Endemic
	*Thymus algeriensis Boiss. & Reut.*	Spontaneous
	*Thymus maroccanus Ball.*	Endemic
	*Thymus munbyanus Boiss. & Reut*	Spontaneous
	*Thymus satureioides Coss.*	Endemic
	*Thymus vulgaris* L.	Spontaneous
	*Thymus zygis* L.	Spontaneous
Lauraceae	*Cinnamomum cassia* (L.) *J. Presl*	Imported
	*Cinnamomum verum J. Presl*	Cultivated
	*Laurus nobilis* L.	Spontaneous
	*Persea americana Mill.*	Cultivated
Leguminosae	*Acacia gummifera Willd.*	Endemic
	*Acacia nilotica* (L.) Delile	Cultivated
	*Acacia senegal* (L.) Willd.	Cultivated
	*Acacia tortilis* (*Forssk.*) *Hayne*	Spontaneous
	*Acacia albida Delile*	Cultivated
	*Anagyris foetida* L.	Cultivated
	*Arachis hypogaea* L.	Cultivated
	*Cassia absus* L.	Imported
	*Cassia fistula* L.	Cultivated
	*Ceratonia siliqua* L.	Imported
	*Cicer arietinum* L.	Cultivated
	*Cytisus battandieri Maire*	Cultivated
	*Glycine max* (L.) *Merr.*	Cultivated
	*Glycyrrhiza glabra* L.	Imported
	*Lupinus albus* L.	Spontaneous
	*Lupinus angustifolius* L.	Spontaneous
	*Lupinus luteus* L.	Spontaneous
	*Lupinus pilosus* L.	Spontaneous
	*Medicago sativa* L.	Cultivated
	*Ononis natrix* L.	Spontaneous
	*Ononis tournefortii Coss.*	Spontaneous
	*Phaseolus aureus Roxb.*	Cultivated
	*Phaseolus vulgaris* L.	Cultivated
	*Retama monosperma* (L.) *Boiss.*	Spontaneous
	*Retama raetam* (*Forssk.*) *Webb*	Spontaneous
	*Retama sphaerocarpa* (L.) *Boiss.*	Spontaneous
	*Senna alexandrina Mill.*	Cultivated
	*Trigonella foenum-graecum* L.	Spontaneous
	*Vicia faba* L.	Spontaneous
	*Vicia sativa* L.	Spontaneous
	*Vigna radiata* (L.) *R. Wilczek*	Cultivated
	*Vigna unguiculata* (L.) *Walp*	Cultivated
	*Urginea maritima* (L.) *Baker*	Cultivated
Linaceae	*Linum usitatissimum* L.	Cultivated
Lythraceae	*Lawsonia inermis* L.	Spontaneous
	*Punica granatum* L.	Cultivated
Malvaceae	*Abelmoschus esculentus* (L.) *Moench*	Cultivated
	*Hibiscus sabdariffa* L.	Spontaneous
Moraceae	*Ficus abelii Miq*	Cultivated
	*Ficus carica* L.	Spontaneous/Cultivated
	*Ficus dottata Gasp.*	Cultivated
	*Morus alba* L.	Spontaneous
	*Morus nigra* L.	Spontaneous
Moringaceae	*Moringa oleifera Lam.*	Cultivated
Musaceae	*Musa paradisiaca* L.	Cultivated
Myristicaceae	*Myristica fragrans Houtt.*	Cultivated
Myrtaceae	*Eucalyptus camaldulensis Dehnh.*	Cultivated
	*Eucalyptus globulus Labill.*	Imported
	*Eugenia caryophyllata Thunb*	Cultivated
	*Jasminum fruticans* L.	Spontaneous
	*Myrtus communis* L.	Imported
	*Syzygium aromaticum* (L.) *Merr. &* L.* M. Perry*	Cultivated
Nitrariaceae	*Peganum harmala* L.	Spontaneous
Oleaceae	*Fraxinus angustifolia Vahl*	Spontaneous
	*Fraxinus excelsior var. acuminata Schur*	Cultivated
	*Olea europaea* L.	Spontaneous/Cultivated
	*Olea europaea* subsp. *maroccana* (*Greuter & Burdet*)	Spontaneous/Cultivated
	*Olea europea* L. *subsp. europaea var. sylvestris* (*Mill*) *Lehr*,	Cultivated
	*Olea oleaster Hoffm. & Link.*	Spontaneous
Papaveraceae	*Fumaria officinalis* L.	Spontaneous
	*Papaver rhoeas* L.	Spontaneous
	*Plantago ovata Forssk.*	Spontaneous
Pedaliaceae	*Sesamum indicum* L.	Imported
Plantaginaceae	*Globularia alypum* L.	Spontaneous
	*Globularia repens Lam.*	Spontaneous
Plumbaginaceae	*Limonium sinuatum* (L.) *Mill.*	Spontaneous
Poaceae	*Avena sativa* L.	Cultivated
	*Avena sterilis* L.	Cultivated
	*Castellia tuberculosa* (*Moris*) *Bor*	Spontaneous
	*Cynodon dactylon* (L.) *Pers.*	Spontaneous
	*Hordeum vulgare* L.	Cultivated
	*Lolium perenne* L.	Cultivated
	*Lolium multiflorum Lam.*	Spontaneous
	*Lolium rigidum Gaudin*	Spontaneous
	*Panicum miliaceum* L.	Spontaneous
	*Panicum turgidum Forssk.*	Spontaneous
	*Pennisetum glaucum* (L.) *R.Br.*	Spontaneous
	*Phalaris canariensis* L.	Spontaneous
	*Phalaris paradoxa* L.	Spontaneous
	*Polypogon monspeliensis* (L.) *Desf*	Spontaneous
	*Sorghum bicolor* (L.) *Moench*	Spontaneous
	*Triticum durum Desf.*	Cultivated
	*Triticum aestivum* L.	Cultivated
	*Triticum turgidum* L.	Spontaneous
	*Zea mays* L.	Cultivated
Polygonaceae	*Emex spinosa* (L.) *Campd.*	Spontaneous
	*Portulaca oleracea* L.	Spontaneous
Ranunculaceae	*Nigella Sativa* L.	Spontaneous
Resedaceae	*Reseda lanceolata Lag.*	Spontaneous
Rhamnaceae	*Ziziphus lotus* (L.) *Lam.*	Spontaneous
	*Ziziphus jujube Mill*	Spontaneous
Rosaceae	*Cydonia oblonga Mill.*	Cultivated
	*Chaenomeles sinensis* (*Dum.Cours.*) *Koehne*	Cultivated
	*Crataegus monogyna Jacq.*	Cultivated
	*Eriobotrya japonica* (*Thunb.*) *Lindl.*	Cultivated
	*Fragaria vesca* L.	Cultivated
	*Malus communis* (L.) *Poir.*	Cultivated
	*Prunus armeniaca* L.	Cultivated
	*Prunus dulcis* (*Mill.*) *D.A. Webb*	Spontaneous
	*Prunus cerasus* L.	Cultivated
	*Rubus fruticosus var. vulgaris* (*Weihe & Nees*	Spontaneous
	*Rubus fruticosus var. ulmifolius*, (*Schott*)	Spontaneous
Rubiaceae	*Rubia tinctorum* L.	Spontaneous
	*Coffea arabica* L.	Cultivated
Rutaceae	*Citrus medica var. limon* L.	Cultivated
	*Citrus paradisi Macfad.*	Cultivated
	*Citrus sinensis* (L.) *Osbeck*	Cultivated
	*Citrus aurantium* L.	Imported
	*Ruta graveolens* L.	Spontaneous
	*Ruta chalepensis* L.	Spontaneous
	*Ruta montana* L.	Spontaneous
Salicaceae	*Salix alba* L.	Cultivated
Salvadoraceae	*Salvadora persica* L.	Cultivated
Santalaceae	*Viscum album L*	Spontaneous
Sapotaceae	*Argania spinosa* (L.) *Skeels*	Cultivated
Schisandraceae	*Illicium verum Hook.f.*	Cultivated
Solanaceae	*Capsicum annuum* L.	Cultivated
	*Datura stramonium* L.	Spontaneous/Cultivated
	*Lycopersicon esculentum Mill.*	Cultivated
	*Nicotiana tabacum* L.	Cultivated
	*Solanum americanum Mill.*	Spontaneous/Cultivated
	*Solanum melongena* L.	Cultivated
	*Withania frutescens* (L.) *Pauquy*	Cultivated
Taxaceae	*Taxus baccata* L.	Spontaneous
Theaceae	*Camellia sinensis* (L.) *Kuntze*	Imported
Thymelaeaceae	*Thymelaea hirsuta* (L.) *Endl.*	Spontaneous
	*Thymelaea tartonraira* (L.) *All.*	Spontaneous
	*Thymelaea virgata* (*Desf.*) *Endl.*	Endemic
	*Aquilaria malaccensis Lam*	Cultivated
Urticaceae	*Urtica dioica* L.	Spontaneous
	*Urtica pilulifera* L.	Spontaneous
	*Urtica urens* L.	Spontaneous
	*Urtica membranacea Poir. ex Savigny*	Spontaneous
Valerianaceae	*Nardostachys jatamansi* (*D. Don*) *DC.*	Imported
Verbenaceae	*Aloysia citriodora Palau*	Cultivated
	*Verbena officinalis* L.	Spontaneous/Cultivated
Vitaceae	*Vitis vinifera* L.	Spontaneous/Cultivated
Xanthorrhoeaceae	*Asphodelus microcarpus Salzm. & Viv.*	Spontaneous
	*Asphodelus tenuifolius Cav.*	Spontaneous
Zingiberaceae	*Zingiber officinale Roscoe.*	Cultivated
	*Curcuma longa* L.	Cultivated
Zygophyllaceae	*Tetraena gaetula* (*Emb. & Maire*) *Beier & Thulin*	Endemic
	*Zygophyllum gaetulum Emb. & Maire*	Spontaneous

**Table 3:** Plants used by Moroccan diabetic patients for type 1, type 2, or gestational diabetes mellitus.

Scientific Name	Type 1 Diabetes	Type 2 Diabetes	Gestational Diabetes Mellitus
*Allium cepa* L.	+	+	-
*Allium sativum* L.	+	+	-
*Allium ampeloprasum var. porrum*	-	+	-
*Aloe vera* (L.) *Burm.f.*	-	+	-
*Beta vulgaris* L.	-	+	-
*Pistacia atlantica Desf.*	-	+	-
*Pistacia lentiscus* L.	+	-	-
*Ammi visnaga* (L.) *Lam.*	+	+	-
*Anethum foeniculum* L.	-	+	-
*Apium graveolens* L.	+	+	-
*Carum carvi* L.	-	+	+
*Coriandrum sativum* L.	+	+	-
*Cuminum cyminum* L.	-	+	-
*Foeniculum vulgare Mill.*	+	+	-
*Petroselinum crispum* (Mill.) Fuss	+	+	-
*Pimpinella anisum* L.	-	+	+
*Ridolfia segetum* (L.) Moris	+	-	-
*Caralluma europaea* (Guss.) N.E.Br.	+	+	-
*Nerium oleander* L.	+	+	-
*Chamaerops humilis* L.	-	+	-
*Phoenix dactylifera* L.	-	-	+
*Asparagus albus* L.	+	-	-
*Achillea odorata* L.	+	-	-
*Achillea santolinoides Lag.*	-	+	-
*Artemisia absinthium* L.	+	+	-
*Artemisia campestris* L.	-	+	-
*Artemisia herba-alba* Asso	+	+	+
*Artemisia mesatlantica Maire*	-	+	-
*Chamaemelum mixtum* (L.) Alloni	-	+	-
*Chamaemelum nobile* (L.) All.	+	+	-
*Chrysanthemum coronarium* L.	+	-	-
*Cladanthus arabicus* (L.) Cass.	+	-	-
*Cynara cardunculus* L.	+	+	-
*Cynara cardunculus* subsp. *scolymus* (L.)	+	+	-
*Dittrichia viscosa* (L.) Greuter	+	-	-
*Lactuca sativa* L.	-	+	-
*Matricaria chamomilla* L.	-	-	+
*Pallenis spinosa* (L.) Cass.	+	-	-
*Saussurea costus* (*Falc.*) *Lipschitz*	-	+	-
*Scolymus hispanicus* L.	-	+	-
*Sonchus asper* (L.) *Hill*	-	+	-
*Sonchus tenerrimus* L.	-	+	-
*Silybum marianum* L.	-	+	-
*Tanacetum vulgare* L.	+	-	-
*Berberis vulgaris* subsp. *Australis* (Boiss.) Heywood	-	+	-
*Anastatica hierochuntica* L.	-	+	+
*Brassica oleracea* L.	-	+	+
*Brassica rapa* L.	+	-	-
*Eruca vesicaria* (L.) Cav.	+	-	-
*Lepidium sativum* L.	+	+	+
*Raphanus raphanistrum* subsp. *sativus* (L.)	+	+	+
*Boswellia sacra* Flueck.	+	+	-
*Opuntia ficus indica* (L.) *Mill.*	-	+	-
*Capparis spinosa* L.	+	+	-
*Cistus laurifolius* L.	+	-	-
*Cistus ladanifer* L.	+	-	-
*Atriplex halimus* L.	-	+	-
*Chenopodium ambrosioides* L.,	+	+	-
*Ipomoea batatas* (L.)	-	+	-
*Bryonia dioica Jacq.*	-	+	-
*Citrullus colocynthis* (L.) Schrad.	+	+	-
*Citrullus vulgaris Schard.*	+	+	-
*Cucumis sativus* L.	-	+	-
*Cucurbita maxima Duchesne*	+	-	-
*Cucurbita pepo* L.	-	+	-
*Juniperus phoenicea* L.	+	+	-
*Juniperus oxycedrus* L.	+	+	-
*Tetraclinis articulata* (Vahl) Mast.	+	+	-
*Cyperus longus* L.	+	-	-
*Cyperus rotundus* L.	-	+	-
*Equisetum ramosissimum Desf*	+	-	-
*Arbutus unedo* L.	+	-	-
*Euphorbia officinarum subsp.echinus*	-	+	-
*Euphorbia officinarum* L.	+	+	-
*Euphorbia peplis* L.	-	-	+
*Euphorbia resinifera* O. Berg	+	+	-
*Mercurialis annua* L.	+	+	-
*Quercus suber* L.	-	+	-
*Quercus ilex* L.	+	-	-
*Centaurium spicatum* (L.) *Fritsch*	+	-	-
*Pelargonium roseum Willd.*	+	-	-
*Crocus sativus* L.	-	+	+
*Juglans regia* L.	+	+	-
*Ajuga iva* (L.) *Schreb.*	-	-	+
*Ballota hirsuta Benth*	+	-	-
*Calamintha officinalis Moench.*	+	-	-
*Calamintha alpina L*	-	+	-
*Lavandula angustifolia Mill*	-	+	-
*Lavandula dentata* L.	-	+	-
*Lavandula maroccana Murb.*	+	-	-
*Lavandula multifida* L.	+	+	-
*Lavandula stoechas* L.	-	-	+
*Marrubium vulgare* L.	+	+	+
*Mentha pulegium* L.	+	+	-
*Melissa officinalis* L.	+	-	-
*Mentha spicata* L.	+	+	-
*Mentha suaveolens Ehrh.*	+	+	-
*Ocimum basilicum* L.	-	-	+
*Origanum compactum Benth.*	-	+	-
*Origanum elongatum* (*Bonnet*)	+	+	-
*Origanum majorana* L.	+	+	-
*Origanum vulgare L*	+	+	-
*Rosmarinus officinalis* L.	+	+	+
*Salvia officinalis* L.	+	+	-
*Teucrium polium* L.	+	+	-
*Thymus broussonetii Boiss.*	+	-	-
*Thymus maroccanus Ball.*	+	+	-
*Thymus satureioides Coss.*	+	+	-
*Thymus vulgaris* L.	-	+	-
*Cinnamomum cassia* (L.) *J. Presl*	+	-	-
*Cinnamomum verum J. Presl*	+	+	-
*Laurus nobilis* L.	-	+	-
*Persea americana Mill.*	+	+	-
*Acacia gummifera Willd.*	-	+	-
*Acacia nilotica* (L.) Delile	-	+	-
*Acacia senegal* (L.) Willd.	-	+	+
*Acacia tortilis* (*Forssk.*) *Hayne*	-	+	-
*Acacia albida Delile*	-	+	-
*Anagyris foetida* L.	-	+	-
*Arachis hypogaea* L.	-	+	-
*Cassia absus* L.	+	-	-
*Cassia fistula* L.	-	+	-
*Ceratonia siliqua* L.	+	+	-
*Cicer arietinum* L.	+	-	-
*Glycine max* (L.) *Merr.*	+	-	-
*Glycyrrhiza glabra L*	+	+	-
*Lupinus albus* L.	+	+	-
*Lupinus angustifolius* L.	-	+	-
*Lupinus luteus* L.	-	+	-
*Medicago sativa* L.	+	-	-
*Ononis natrix* L.	-	+	-
*Phaseolus aureus Roxb.*	-	+	-
*Phaseolus vulgaris* L.	+	+	-
*Retama monosperma* (L.) *Boiss.*	-	+	-
*Retama raetam* (*Forssk.*) *Webb*	-	+	-
*Trigonella foenum-graecum* L.	+	+	+
*Vicia faba* L.	+	-	-
*Vigna radiata* (L.) *R. Wilczek*	-	+	-
*Linum usitatissimum* L.	+	+	-
*Punica granatum* L.	-	+	-
*Abelmoschus esculentus* (L.) *Moench*	+	+	-
*Hibiscus sabdariffa* L.	+	-	-
*Ficus carica* L.	+	+	-
*Ficus dottata Gasp.*	-	-	+
*Morus alba* L.	-	+	-
*Morus nigra* L.	-	+	-
*Myristica fragrans Houtt.*	-	+	-
*Eucalyptus camaldulensis Dehnh.*	-	+	-
*Eucalyptus globulus Labill.*	+	-	-
*Eugenia caryophyllata Thunb*	+	-	-
*Jasminum fruticans* L.	+	+	-
*Myrtus communis* L.	-	+	-
*Syzygium aromaticum* L.	+	+	-
*Peganum harmala* L.	-	+	-
*Olea europaea* L.	+	+	+
*Olea europaea* subsp. *maroccana*	+	+	-
*O. europea* L. *subsp. europaea var. sylvestris*	+	+	-
*O. oleaster Hoffm. & Link.*	+	-	-
*Fumaria officinalis* L.	+	-	-
*Plantago ovata Forssk.*	-	+	-
*Sesamum indicum* L.	+	+	+
*Globularia alypum* L.	+	-	-
*Avena sativa* L.	+	+	-
*Avena sterilis* L.	+	-	-
*Castellia tuberculosa Moris*	+	-	-
*Hordeum vulgare* L.	+	+	-
*Lolium perenne* L.	-	-	+
*Lolium rigidum Gaudin*	-	+	-
*Panicum miliaceum* L.	-	+	-
*Pennisetum glaucum* L.	-	+	-
*Phalaris canariensis* L.	-	+	-
*Sorghum bicolor* L.	-	+	-
*Triticum durum Desf.*	+	+	-
*Triticum aestivum* L.	-	+	-
*Triticum turgidum* L.	-	+	-
*Portulaca oleracea* L.	+	-	-
*Nigella Sativa* L.	+	+	-
*Ziziphus lotus* L.	-	+	-
*Ziziphus jujube Mill*	-	+	-
*Chaenomeles sinensis Dum.Cours.*	-	+	-
*Eriobotrya japonica Thunb.*	-	+	-
*Malus communis* L.	+	-	-
*Prunus armeniaca* L.	+	-	-
*Prunus dulcis Mill.*	-	+	-
*Rubus fruticosus var. vulgaris*	-	+	-
*Rubus fruticosus var. ulmifolius*, (*Schott*)	-	-	+
*Rubia tinctorum* L.	-	+	-
*Coffea arabica* L.	+	+	-
*Citrus medica var. limon* L.	+	+	+
*Citrus paradisi Macfad.*	+	+	-
*Citrus sinensis* L.	-	+	-
*Citrus aurantium* L.	+	+	-
*Ruta graveolens* L.	+	-	-
*Ruta chalepensis* L.	-	+	-
*Ruta montana* L.	+	+	-
*Salvadora persica* L.	-	+	-
*Viscum album* L.	-	+	-
*Argania spinosa* L.	+	+	-
*Illicium verum Hook.f.*	-	+	-
*Capsicum annuum* L.	-	+	-
*Lycopersicon esculentum Mill.*	+	+	-
*Solanum melongena* L.	+	+	-
*Withania frutescens* L.	+	-	-
*Taxus baccata* L.	+	-	-
*Camellia sinensis* L.	+	+	-
*Thymelaea hirsuta* L.	+	+	-
*Thymelaea tartonraira* L.	-	+	-
*Thymelaea virgata Desf.*	+	-	-
*Aquilaria malaccensis Lam*	+	+	-
*Urtica urens* L.	+	-	-
*Nardostachys jatamansi D. Don*	+	-	-
*Aloysia citriodora Palau*	-	+	+
*Verbena officinalis* L.	+	-	-
*Vitis vinifera* L.	-	+	-
*Aloe succotrina Lam.*	+	-	+
*Asphodelus microcarpus Salzm. & Viv.*	-	-	+
*Asphodelus tenuifolius Cav.*	-	-	+
*Zingiber officinale Roscoe.*	+	+	-
*Curcuma longa* L.	-	+	-
*Tetraena gaetula Emb. & Maire*	-	-	+
*Zygophyllum gaetulum Emb. &Maire*	+	+	-
